# Urothelial cancer gene regulatory networks inferred from large-scale RNAseq, Bead and Oligo gene expression data

**DOI:** 10.1186/s12918-015-0165-z

**Published:** 2015-05-14

**Authors:** Ricardo de Matos Simoes, Sabine Dalleau, Kate E Williamson, Frank Emmert-Streib

**Affiliations:** Centre for Cancer Research and Cell Biology (CCRCB), Queens University Belfast, 97 Lisburn Road, Belfast, County Antrim, Northern Ireland UK; Computational Medicine and Statistical Learning Laboratory, Department of Signal Processing, Tampere University of Technology, Tampere, 33720 Finland; Institute of Biosciences and Medical Technology, Tampere, 33520 Finland

**Keywords:** Gene regulatory network, BC3Net, Urothelial cancer, Computational genomics

## Abstract

**Background:**

Urothelial pathogenesis is a complex process driven by an underlying network of interconnected genes. The identification of novel genomic target regions and gene targets that drive urothelial carcinogenesis is crucial in order to improve our current limited understanding of urothelial cancer (UC) on the molecular level. The inference of genome-wide gene regulatory networks (GRN) from large-scale gene expression data provides a promising approach for a detailed investigation of the underlying network structure associated to urothelial carcinogenesis.

**Methods:**

In our study we inferred and compared three GRNs by the application of the BC3Net inference algorithm to large-scale transitional cell carcinoma gene expression data sets from Illumina RNAseq (179 samples), Illumina Bead arrays (165 samples) and Affymetrix Oligo microarrays (188 samples). We investigated the structural and functional properties of GRNs for the identification of molecular targets associated to urothelial cancer.

**Results:**

We found that the urothelial cancer (UC) GRNs show a significant enrichment of subnetworks that are associated with known cancer hallmarks including cell cycle, immune response, signaling, differentiation and translation. Interestingly, the most prominent subnetworks of co-located genes were found on chromosome regions 5q31.3 (RNAseq), 8q24.3 (Oligo) and 1q23.3 (Bead), which all represent known genomic regions frequently deregulated or aberated in urothelial cancer and other cancer types. Furthermore, the identified hub genes of the individual GRNs, e.g., HID1/DMC1 (tumor development), RNF17/TDRD4 (cancer antigen) and CYP4A11 (angiogenesis/ metastasis) are known cancer associated markers. The GRNs were highly dataset specific on the interaction level between individual genes, but showed large similarities on the biological function level represented by subnetworks. Remarkably, the RNAseq UC GRN showed twice the proportion of significant functional subnetworks. Based on our analysis of inferential and experimental networks the Bead UC GRN showed the lowest performance compared to the RNAseq and Oligo UC GRNs.

**Conclusion:**

To our knowledge, this is the first study investigating genome-scale UC GRNs. RNAseq based gene expression data is the data platform of choice for a GRN inference. Our study offers new avenues for the identification of novel putative diagnostic targets for subsequent studies in bladder tumors.

**Electronic supplementary material:**

The online version of this article (doi:10.1186/s12918-015-0165-z) contains supplementary material, which is available to authorized users.

## Background

Urothelial cancer (UC) is a heterogeneous disease with risk factors that include smoking, contact to chemicals and age [1]. Urothelial tumors originate from the epithelial lining of the bladder and can progress from non-invasive to more aggressive muscle-invasive subtypes which penetrate the deeper tissue layers of the bladder. The non-invasive tumor stages can be treated by transurethral resection, chemo- and intravesical therapy, whereas for invasive stages cystectomy, radiotherapy and chemotherapy are preferred [1,2]. Monitoring of UC is very expensive as its recurrence rate is high [3]. An understanding of the mechanistic interplay between individual genes and proteins that drive the development and progression of UC is therefore a high priority. System-based approaches allow us to investigate the underlying network structure associated with carcinogenesis and thus facilitate a novel perspective for the identification of molecular targets that drive urothelial carcinogenesis. The inference of gene regulatory networks (GRN) from large-scale gene expression data of tumor samples from various grades and stages is a promising approach for the identification of novel putative targets in cancer [4-6].

A GRN is a mathematical description of the dependencies within a gene expression dataset. Currently, a large arsenal of gene regulatory network inference methods have been developed [7-9]. The most popular methods are based on mutual information which is a dependency measure that can be estimated for all pairs of genes in a gene expression dataset. In this study we infer GRNs by the application of the BC3Net algorithm which is based on the aggregation of an ensemble of C3Net gene regulatory networks [4]. The C3Net algorithm selects a maximum of one gene neighbor for each gene on the basis of the strongest mutual dependency that is statistically significant. For a gene expression dataset with *n* genes we thus obtain a sparse network with at most *n* interactions. The BC3Net generates an ensemble of C3Net networks from bootstrap datasets, i.e., by sampling a dataset with replacement, that are subsequently aggregated to a weighted network. We have reported that BC3Net was shown to produce biological meaningful results [4-6,10,11]. The hub genes of GRNs that were inferred from large-scale cancer gene expression data were observed to provide promising putative novel target genes for cancer such as G-protein coupled receptors and transmembrane proteins [10,12].

## Methods

### Preprocessing and sample information for the Illumina RNAseq, Bead array and Affymetrix Oligo microarray gene expression dataset

We preprocessed three large-scale urothelial cancer gene expression datasets from a) Illumina RNAseq (179 samples) [13], b) Illumina Bead array (165 samples) GSE13507 [14,15] and c) Affymetrix oligo microarray (188 samples) platform [16-19]. An overview of the tumor stage information for the three datasets is shown in Table 1. In this table we distinguish 6 tumor stages, namely, pTcis, pTa, pT1, pT2, pT3 and pT4. For each of these stages we list the number of available samples provided by the three platforms.

**Table 1:** **Tumor stages across the three datasets**

**Tumor stage**	**RNAseq**	**Bead**	**Oligo**
	**(# samples)**	**(# samples)**	**(# samples)**
pTcis			5
pTa	1	24	35
pT1	1	80	16
pT2	57	31	22
pT3	91	19	51
pT4	29	11	29
pT2-4			30
Total	179	165	188
NMI	2	104	56
MI	177	61	132
Genes	20,161	18,956	12,495

The RNAseq dataset consists mainly of muscle-invasive UC tumor samples. 30 samples of the Oligo dataset corresponded to the muscle invasive stages pT2 to pT4 and were not assigned a specific stage.

#### RNAseq gene expression dataset from TCGA

The RNAseq gene expression dataset was retrieved from *The Cancer Genome Atlas* bladder cancer TCGA project [http://cancergenome.nih.gov/] [13]. We used the preprocessed *RNAseqV2* normalized count expression values based on *RSEM* (RNA-Seq by Expectation-Maximization) [20,21] as provided by TCGA and clinical information such as the TCGA *barcode identifier*, *sample type* and *tumor histology* by the *bcr_aliquot_uuid* identifiers. We extracted gene expression data of primary solid tumors for a total of 179 samples with histology stage information (*march 2014*). A total of 177 of the 179 selected tumor samples represent muscle invasive carcinoma stage *pT2* or above. We performed a log-transformation *log*_*e*_(1+*p*) on the count expression values. The resulting gene expression matrix consisted of 20,161 entrez genes and 179 samples. Genes with a zero standard deviation were removed from the dataset.

#### Illumina Bead array gene expression dataset

We used the processed matrix series Illumina Bead gene expression data from GEO GSE13507 [14,15]. The dataset comprises 257 samples from the *Illumina human-6 v2.0 expression Beadchip* microarray platform. The dataset consists of tumor samples from 62 muscle invasive, 104 non-muscle invasive, 23 recurrent non-invasive bladder cancer and samples that we excluded for the network inference representing 58 mucosae surrounding cancer and 10 normal mucosae. We assigned Illumina identifiers to entrez gene id and gene symbols using the *illuminaHumanv2.db* annotation bioconductor package. From the 43,148 Illumina identifiers for a total of 20,481 an entrez identifier was available. The remaining 22,667 features were not considered for the analysis. In total, we selected 165 primary bladder cancer samples (Table 1).

#### Affymetrix Oligo microarray gene expression dataset

We used a third UC dataset from Affymetrix gene expression data comprising 183 samples from 4 different datasets. We extracted 93 (U133plus2) samples from GSE31684 [16], 46 (U133A) samples from GSE3167 [17], 30 (U133A) samples from GSE5287 [18] and 19 (U133A) samples from GSE37317 [19]. We considered only probe sets that were present in both array types *U133a* and *U133plus2*. We combined the *U133plus2* samples and the *U133a* samples using the *matchprobes* bioconductor package [22]. We normalized the microarray samples using RMA and quantile normalization [23] using *log*_2_ expression intensities for each probe set. As a summary statistic for multiple probesets that match to the same entrez gene identifiers we used the median expression value. Entrez gene ID to Affymetrix probe set annotation was obtained from the *hgu133plus2.db* and *hgu133a.db* R package. We excluded all probe sets from our analysis that remained unmapped to entrez identifiers. The resulting expression dataset consisted of 12,495 genes and 188 samples.

### BC3Net gene regulatory network inference

We inferred our bladder cancer GRN using C3Net and the “B”agging version of the C3Net [24,25] algorithm called BC3Net [4]. The BC3Net infers an ensemble of C3Net gene regulatory networks from bootstrap generated datasets that are subsequently aggregated to a weighted GRN. We defined an ensemble of *B*=100 independent bootstrap datasets $\{{D^{b}_{k}}\}_{k=1}^{B}$ that were generated from a given gene expression dataset *D*. For each bootstrap data set ${D_{k}^{b}}$ a GRN ${G^{b}_{k}}$ was inferred using C3Net [24,25]. Edges with non-significant mutual information values were subsequently rejected using a non-parametric test with a Bonferroni multiple testing correction for a significance level *α*=0.05. The null distribution of mutual information is generated from sample-gene label permutations of the original gene expression matrix. For the network inference we used a Pearson Estimator [8,26]

(1) $$ I(X,Y) = -\frac{1}{2} log(1 - \rho^{2}),  $$

where *ρ* denotes the Pearson correlation coefficient. The inferred ensemble of GRNs $\{{G^{b}_{k}}\}_{k=1}^{B}$ was aggregated into a weighted network ${G_{w}^{b}}$. The weights of the inferred interactions give the frequency how often an interaction was observed in the C3Net network ensemble and are denoted as ensemble consensus rate (ECR). For each inferred weighted edge in the network the statistical significance was estimated by a Binomial test. For multiple testing correction Bonferroni was used with a significance level *α*=0.05.

### Relevance networks

For the inference of relevance networks [27] we used the WGCNA R Package [28] and the CLR [29] implementation provided in the minet R-Package [30]. Interactions were defined for WGCNA by *hard* thresholds on the absolute Pearson correlation matrix and for CLR by *hard* thresholds on the z-score transformed mutual information matrix that was estimated using a Pearson Estimator [8,26].

### Cancer census genes

The Cancer Gene Census (CGC) [31] (version download *10-01-2014*) [http://www.sanger.ac.uk/genetics/CGP/Census/] provides information about genes with somatic mutations that are associated to different types of cancer. We used the entrez identifiers of the defined cancer census genes.

### Gene ontology gene sets

For our analysis, we obtained the Gene Ontology [32] annotation for entrez gene IDs from Bioconductor [22] annotation packages *org.Hs.eg.db* and *GO.db*.

### Gene family gene sets

We retrieved gene family protein tag information and entrez identifiers of the genes in the HGNC database [http://www.genenames.org/genefamilies]. We defined gene family gene sets for groups of genes that shared the same HGNC protein family tag. From the HGNC database we gathered a total of 587 gene family gene sets comprising a total of 16,722 entrez genes.

### Gene sets of co-localized adjacent genes

For the identification of genomic regions with enriched subnetworks of interacting genes we defined gene sets of genes that were adjacently located within a chromosomal region (co-located) from overlaping sliding windows along the human chromosomes. We defined gene sets from 1*Mb* (mega bases) sliding windows along the human chromosomes with a 500*Kb* (kilo bases) overlap between adjacent windows. The gene sets of co-located genes were defined for chromosome regions of 1*Mb* with 500*Kb* overlap to mimic the extend for co-expressed gene clusters [33].

### Gene pair enrichment analysis (GPEA)

The GPEA facilitates the identification and ranking of significant subnetworks of defined gene sets for a given network. For *p* genes there is a total of *N*=*p*(*p*−1)/2 different gene pairs. If there are *p*_*S*_ genes for a particular gene set (S) then the total number of gene pairs for this gene set is *m*_*S*_=*p*_*S*_(*p*_*S*_−1)/2. When a network *G* contains *n* interactions, of which *k* interactions are among genes from the given gene set *S*, then a p-value for the enrichment of gene pairs of this gene set *S* can be calculated from the following hypergeometric distribution

(2) $$ p(k|S) = \sum\limits_{i=k}^{m_{S}} P(X=i | S) = \sum\limits_{i=k}^{m_{S}} \frac{{m_{S} \choose {i}} {N-m_{S} \choose {n-i}}}{{N \choose {n}}}   $$

This p-value gives an estimate for the probability to observe *k* or more interactions between genes from a given gene set *S*.

We performed a GPEA analysis for the inferred GRNs for ∼ 8,000 gene sets of GO biological process (≥3 and <1000 genes), ∼ 500 gene sets of gene families (≥3 genes) and ∼ 4,000 gene sets of co-located genes (≥3 genes). For the analysis the inferred networks are expected to show a strong association to gene sets of a biological functional and spatial context. Therefore, we considered a more stringent significance level of *α*=0.001 (10^−^3) relative to the number of performed test in the range of 10^3^. Further, we considered a Bonferroni multiple testing correction.

### Network centrality measures

For the network analysis we measured the degree centrality and edge density [34]. The degree centrality was defined as the total number of direct neighbors of a gene *g*_*i*_ of an undirected gene regulatory network. The edge density of a network was the number of edges divided by the maximal number of possible edges. For an undirected network this number was given by *n*(*n*−1)/2, whereas *n* is the total number of genes.

### Protein interaction databases

We gathered and processed interactions from Biogrid [35] (15,337 genes, 135,732 interactions; version *biogrid.3.2.11*), Intact [36] (10,029 genes, 63,968 interactions; version *intact.230314*), Mint [37] (7,106 genes, 26,834 interactions; version *mint.2013-03-26*), Hprd [38] (9,672 genes, 39,233; version *hprd.072010*), String [39] (20,770 genes, 4,850,628 interactions, *version 9.1*). Further, we considered the largest manually curated human signaling network [40] (6,306 genes and 57,090 interactions, *version 6*) which we denote in the text as SingNet (http://www.cancer-systemsbiology.org/dataandsoftware.htm), a pathway protein interaction network extracted from the bioconductor package graphite [41] (6,243 genes, 78,201 interactions; *KEGG, Reactome, NCI and Spike*) and the integrative network from ConsensusPathDB (CPDB) [42] (16,619 genes, 485,277 interactions; *version Dec 2014*). We assigned their entrez gene identifiers mapping when available from the interaction database or converted the identifiers (e.g. uniprot identifiers) to entrez identifiers using the annotation from the bioconductor package *org.Hs.eg.db* and uniprot database [43].

### Quantitative comparison of experimental interactions in RNAseq, Bead and Oligo UC GRN

We used the interactions from the Biogrid, Intact, Mint, Hprd, CPDB, SingNet, graphite and String database seperately as global reference networks for the GRN and measured the number of true, false positive (TP, FP), true, false negatives (TN, FN) and F-score to compare the performance of the three inferred gene regulatory networks. The F-score measure $F=2\frac {PR}{P+R}$ gives a weighted average of the precision $P= \frac {TP}{TP+FP}$ and recall $R=\frac {TP}{TP+FN}$.

For the local subnetwork based network inference performance comparisons we used the String network as a reference network. We compared the cumulative log transformed F-score distribution separately for commonly significant GO Biological Process, genomic co-located genes and gene family subnetworks between the Oligo, Bead and RNAseq UC GRNs.

For all subnetworks and pairwise network comparisons we performed a hypergeometric test for the number of shared interactions between two networks is not larger than expected by random chance. For the subnetwork analysis we consider FDR multiple testing correction [44].

## Results

### Urothelial cancer (UC) gene regulatory networks (GRN)

**Figure 1:**
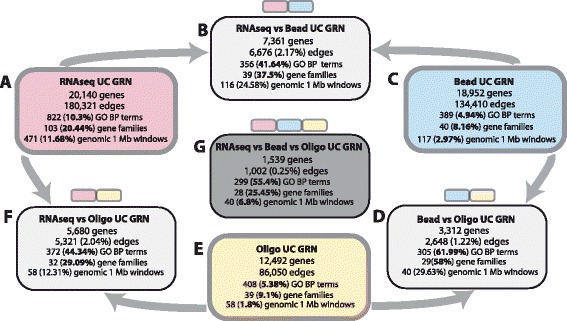
Overview of the comparisons between the inferred RNAseq **(A)**, Bead **(C)** and Oligo **(E)** UC GRN. The pairwise comparisons of the three networks are shown in **(B, D, F)** and triple comparison in **(G)**. We compared the networks on the gene interaction level and gene set subnetwork level for Gene Ontology biological processes, gene families and co-located gene sets of 1 Mb genomic regions.

For the identification of molecular targets for UC from a network-based perspective we inferred BC3net GRNs from RNAseq, Bead and Oligo UC gene expression datasets. The giant connected component of the inferred RNAseq UC GRN consisted of 18,952 genes, the Bead UC GRN of 20,140 genes and the Oligo UC consisted of 12,492 genes (Figure 1A). In the following we compared the global network and local structural properties between the three networks.

The global network properties among the three networks were highly similar. The edge density (*d*∼0.001) of the Oligo has a slightly higher edge density compared to the RNAseq and Bead UC GRN. The degree distribution of the UC GRN follow a power law distribution with exponents *α*_RNAseq_=4.09, *α*_Bead_=4.16*α*_Oligo_=3.73. The average shortest path length for all three networks was *p*_RNAseq_=4.17, *p*_Bead_=4.45, *p*_Oligo_=4.43 genes (measured with the Dijkstra distance [45]).

On the local structural level of individual interactions we observed that the three networks are highly dissimilar. We performed a pairwise comparison and joint comparison of all three networks to quantify the number of shared interactions. The percentages of shared interactions were quantified from the union of all interactions of two networks and for the joint comparison from the union of all interactions of the three networks. The GRN networks shared only a total of 6,676 (2.17*%*, RNAseq/Bead), 5,321 (2.04*%*, RNAseq/Oligo) and 2,648 (1.22*%*, Bead/Oligo) interactions which corresponded to subnetworks among 3,312 to 7,361 genes. In total, we found that only 1,002 (0.25*%*) interactions were shared across the three GRNs and corresponded to a subnetwork among 1,539 genes. An overview of our gene expression data and inferred gene regulatory networks on the interaction level is shown in Figure 1.

### Functional analysis of the inferred Oligo, Bead and RNAseq UC GRN

In this section we highlighted the key biological processes of the three UC GRNs and their association to known cancer genes and performed a comparative analysis between inferred GRN, relevance and PPI networks. We identified the most prominent subnetworks for known biological processes of the inferred Oligo, Bead and RNAseq UC GRN by a functional enrichment analysis for gene pairs (GPEA). The association of the identified biological processes to known cancer hallmarks was quantified by a subsequent enrichment analysis of cancer census genes.

The GPEA analysis was performed for Gene Onotology (GO) biological process for all terms with ≥3 and <1000 genes. For the RNAseq UC GRN we observed a total of 10.3*%* significant GO terms. In contrast, for the Bead and Oligo UC GRN only 4.94*%* and 5.38*%* of all tested GO terms were significant Figure 1. For all GRN networks we observed that 50% of the identified significant GO terms were also enriched by cancer census genes. A total of 91% (RNAseq), 88% (Bead) and 93% (Oligo) of the cancer genes were present in the selected set of significant Gene Ontology biological processes.

From all significant GO terms that we identified 299 (∼55.4*%*) GO terms were common across the three UC GRNs. We observed a wide variety of common biological processes with a pronounced representation of immune related processes, cell cycle, catabolic processes such as proteolysis, chromatin organization, metabolism, cell adhesion, cell migration, cell differentiation and development including keratinization and angiogenesis. A complete list of the significant terms for the individual analyses is given in the Additional file 1: Tables S1, S2 and S3. An overview of the functional landscape of the common significant terms among the GRN networks is shown in Additional file 1: Figure S1.

**Figure 2:**
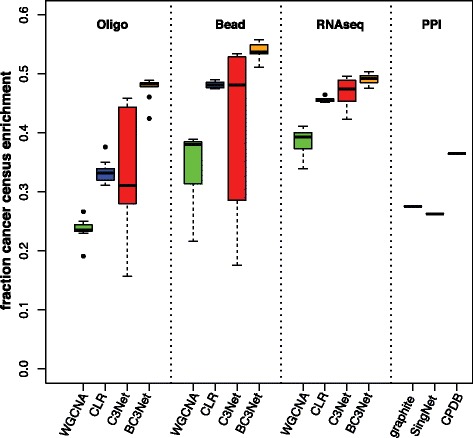
GPEA analysis of GO biological process gene sets for relevance networks, C3Net and BC3Net GRNs. Shown are the fraction of significant GO biological process terms with significant enrichment of cancer census genes.

In order to evaluate our results we compared the fraction of cancer associated biological processes between the BC3Net and C3Net GRN, WGCNA and CLR relevance networks and PPI networks from graphite, SingNet and CPDB. The analysis was performed separately for the Oligo, Bead and RNAseq gene expression data. For the analysis we generated relevance networks by hard thresholds for 0.1 to 0.9 percentiles of the absolute correlation matrix (WGCNA) and the z-score transformed matrix (CLR). C3Net inferred interactions were weighted by the respective mutual information value and for BC3Net by the ensemble consensus rate (ECR). For C3Net and BC3Net the analysis was performed on the entire network and for an ensemble of hard thresholds ranging from 0.1 to 0.9 percentiles. Figure 2 shows the fraction of biological processes that were identified from the GPEA analysis (*α*=0.001, Bonferroni) with a significant enrichment of cancer census genes. For all 3 datasets the BC3Net showed the largest fraction of cancer associated significant biological processes (∼50*%*). CLR and WGCNA showed a low performance on the Oligo gene expression dataset (25*%* to 35*%*) that is comparable to PPI networks. We also observed that CLR shows a prominently improved performance compared to WGCNA.

### Gene pair enrichment analysis of gene subnetworks of co-located genes

Gene expression profiles that are influenced by genomic and epigenomic alterations can elucidate dependency structures of co-located genes and link to novel genomic target regions which are specific to urothelial cancer. In order to identify genomic cancer target regions with significant subnetworks in the RNAseq, Bead and Oligo GRNs we perfromed an enrichment analysis for gene pairs in gene sets from genome-wide 1 Mb genomic regions of co-located genes.

**Figure 3:**
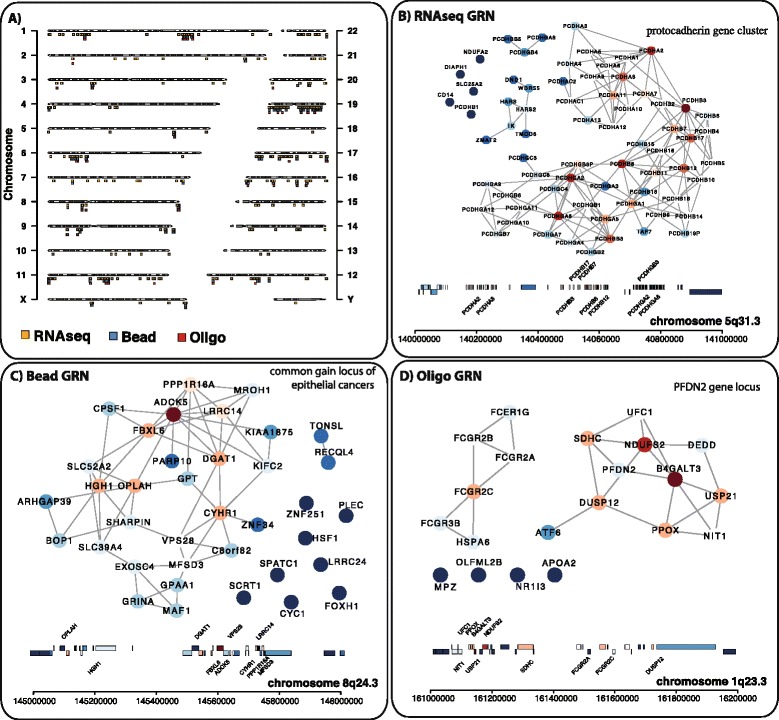
Significant genomic UC GRN subnetworks defined from co-located genes of 1 Mb genomic regions.**(A)** Overview of the GPEA analysis showing all significant genomic subnetworks for the RNAseq (yellow), Bead (blue) and Oligo (red) UC GRNs. **(B-D)** Shown are the most prominent genomic subnetworks derived from the RNAseq **(B)**, Bead **(C)** and Oligo **(D)** UC GRNs.

We observed 11.68*%* significantly co-located gene subnetworks for the RNAseq UC GRN. In contrast, for the Bead and Oligo UC GRN we identified only 2−3*%*. Figure 3 shows the most prominent co-located gene subnetwork for the GRNs of the RNAseq, Bead array and Oligo UC dataset. For the three GRNs the top 50 chromosomal regions with a significant GRN subnetwork are shown in Tables 2, 3 and 4 (for full tables see Additional file 1: Tables S4, S5 and S6). We reviewed the literature for the most prominent identified genomic region and their association to UC for each GRN. For the RNA-seq UC GRN the most prominent gene subnetwork was located on chromosome locus 5*q*31.3 and represents a protocadherin gene cluster. In UC, the loci 5*q*31.2−*q*32 has been associated with losses in a low fraction of UC tumors [46,47]. In [48,49] an epigenetic analysis was performed on free DNA derived from blood serum samples from UC patients of the protocadherin PCDH10 an PCDH8. The studies of [48,49] showed that the methylation patterns of *PCDH10* and *PCDH8* were significantly associated with stage, grade, recurrence and tumor size. The 5*q*31.3 locus was also described in Wilms tumor to be epigentically silenced [50]. For the Bead UC GRN the most prominent co-located gene subnetwork was located on chromosome locus 8*q*24.3. The 8*q*24.3 locus is a common gain loci in multiple cancers and was also confirmed in multiple UC cell lines [51]. For the Oligo UC GRN the chromosome locus 1*q*23.3 contained the most prominent co-located gene subnetwork. In [52] a gain of 1*q*23.3 was identified from free DNA in urine samples from UC patients. Further, [52] validated a selected candidate PFND2 that is located in 1*q*23.3 in an independent set of urothelial cancer tumors. PFND2 was significantly amplified and overexpressed and showed association to increasing stage and tumor grade.

**Table 2:** **GPEA analysis of 1 Mb genomic regions gene sets for the RNAseq UC GRN**

**chr**	**Locus**	**Start**	**Size**	**Edges**	**p-value**	**Census**
chr5	q31.3	140000001	74	159	3.6673e-222	
chr17	q21.2	39000001	61	136	7.6570e-204	
chr17	q21.2	38500001	49	119	1.1284e-194	RARA, SMARCE1
chr6	p22.2	25500001	50	105	1.0881e-163	
chr6	p22.2/p22.1	26000001	48	98	2.9016e-153	
chr5	q31.3	140500001	54	103	1.6926e-152	
chr19	q13.43	57500001	41	90	3.9091e-150	
chr8	q24.3	145000001	43	81	1.5314e-127	RECQL4
chr19	q13.43	58000001	46	75	8.4630e-111	
chr16	p11.2	30000001	54	80	4.0300e-109	
chr21	q22.11	31500001	38	67	5.2390e-107	
chr1	q21.3	152500001	43	69	2.0553e-103	
chr9	q34.3	139500001	67	83	7.6570e-99	
chr17	q25.3	79500001	47	67	3.9091e-94	ASPSCR1
chr19	q13.31	44000001	37	60	4.0300e-94	
chr16	p11.2	30500001	49	68	1.7329e-93	FUS
chr8	q24.3	145500001	37	58	4.8360e-90	RECQL4
chr11	q13.1/q13.2	65000001	46	64	8.8660e-90	
chr3	p25.3	9500001	34	55	3.1837e-88	FANCD2, VHL
chr5	q31.3	139500001	42	60	3.1434e-87	
chr6	p22.1	27000001	32	51	8.8660e-83	HIST1H4I
chr1	q21.3	152000001	34	52	5.2390e-82	
chr16	p11.2	29500001	45	59	1.1687e-81	
chrX	p11.23	48500001	50	62	1.8941e-81	GATA1, TFE3, WAS
chr6	p21.33/p21.32	31500001	79	78	1.2896e-79	
chr19	p13.3	500001	51	60	7.6570e-77	FSTL3, STK11
chr1	p36.33	1000001	48	58	1.7732e-76	
chr19	p13.3	1000001	43	54	1.1687e-74	STK11, TCF3
chr1	q21.3	150500001	35	48	1.5314e-72	MLLT11, ARNT
chr1	q22	155000001	42	51	3.3852e-70	MUC1
chr8	q24.3	144500001	39	49	9.2690e-70	
chr19	q13.41/q13.42	53000001	31	44	1.6926e-69	
chr19	p13.11/p12	19000001	33	45	5.2390e-69	
chr16	p13.3	1	52	56	5.6420e-69	AXIN1
chrX	p11.23/p11.22	49000001	44	51	4.4330e-68	
chr11	q13.1/q13.2	65500001	46	52	7.6570e-68	
chr6	p22.1	27500001	36	46	2.1359e-67	
chr19	q13.33	49500001	70	64	2.9822e-66	
chr1	q23.3	161000001	36	45	1.8941e-65	FCGR2B, SDHC
chr19	q13.31/q13.32	44500001	36	45	1.8941e-65	CBLC, BCL3
chr3	p21.31	48500001	42	48	1.1284e-64	NCKIPSD
chr19	q13.43	58500001	30	41	1.4911e-64	
chr16	p13.3	1500001	59	57	2.1762e-64	TSC2, TRAF7
chr1	q21.3	151000001	37	45	2.4583e-64	MLLT11
chr11	p15.5	1	50	52	5.2390e-64	HRAS
chr1	q21.3/q22	154500001	42	47	7.6570e-63	MUC1
chr19	q13.12	36000001	48	50	1.9747e-62	
chr11	q13.1	64500001	56	54	4.4330e-62	MEN1
chr19	p13.2	11500001	30	39	1.9747e-60	
chr11	q13.2	67000001	36	42	1.1284e-59	

For each significant genomic region the chromosome (chr), chromosome band (locus), nucleotide base start site of the genomic region (start), number of genes of the gene set (size), number of edges of the significant subnetwork (edges), Bonferroni adjusted p-value of the subnetwork (p-value) and a list of genes in the significant subnetwork that are present in the cancer census (census).

**Table 3:** **GPEA analysis of 1 Mb genomic regions gene sets for the Bead UC GRN**

**chr**	**Locus**	**Start**	**Size**	**Edges**	**p-value**	**Census**
chr8	q24.3	145000001	43	60	2.08131e-90	RECQL4
chr6	p22.2/p22.1	26000001	48	54	2.23839e-73	
chr6	p22.2	25500001	50	54	2.00277e-71	
chr1	q21.3	152500001	43	46	1.76715e-63	
chr17	q25.3	79500001	47	41	4.31970e-51	ASPSCR1
chr6	p22.1	27000001	32	34	7.06860e-51	HIST1H4I
chr8	q24.3	145500001	37	35	2.51328e-48	RECQL4
chr8	q24.3	144500001	39	30	1.02102e-37	
chr8	p11.23/p11.22	37500001	20	21	3.49503e-35	WHSC1L1
chr1	q23.3	161000001	36	27	2.04204e-34	FCGR2B, SDHC
chr11	q13.2	67000001	36	25	7.06860e-31	
chr17	q21.32	46000001	31	23	1.80642e-30	
chr17	q21.32/q21.33	46500001	31	23	1.80642e-30	
chr1	q21.3	153000001	43	27	3.29868e-30	
chr11	p15.5	1	50	29	1.06029e-29	HRAS
chr16	p13.3	1	52	29	1.02102e-28	AXIN1
chr19	q13.43	57500001	41	25	5.10510e-28	
chr1	q23.3	160500001	34	22	7.85400e-27	SDHC
chr1	p34.3/p34.2	40000001	23	18	2.74890e-26	MYCL1
chr6	p22.1	27500001	36	22	1.02102e-25	
chr1	p34.2	40500001	22	17	5.89050e-25	
chr1	q21.3	150500001	35	20	7.46130e-23	MLLT11, ARNT
chr1	q21.3/q22	154500001	42	22	9.42480e-23	MUC1
chr6	p21.32/p21.31	32500001	42	22	9.42480e-23	DAXX
chr9	q34.3	139500001	67	28	2.98452e-21	
chr19	q13.43	58000001	46	22	4.71240e-21	
chr1	q21.3	151000001	37	19	2.82744e-20	MLLT11
chr16	p13.3	500001	48	22	3.10233e-20	
chr3	p21.31	49500001	38	19	7.85400e-20	
chr1	q22	155000001	42	20	1.09956e-19	MUC1
chr11	q13.2	66500001	35	18	1.49226e-19	
chr5	q31.3	140000001	74	28	5.89050e-19	
chr22	q13.33	50000001	30	16	1.68861e-18	
chr4	q13.2/q13.3	69500001	9	9	2.67036e-17	
chr6	p21.1	42500001	33	16	3.69138e-17	
chr12	q15	69000001	15	11	3.76992e-17	MDM2
chr12	q13.3/q14.1	57500001	37	17	3.92700e-17	CDK4, DDIT3
chr17	q11.2	26500001	49	20	4.71240e-17	
chr19	p13.2	7500001	42	18	1.02102e-16	
chr9	q34.3	140000001	35	16	2.43474e-16	
chr20	q13.12	43500001	39	17	2.43474e-16	SDC4
chr22	q13.33	50500001	32	15	5.89050e-16	
chr5	q31.3	140500001	54	20	2.04204e-15	
chr1	q21.3	152000001	34	15	3.76992e-15	
chr7	p15.2/p15.1	27000001	22	12	3.76992e-15	JAZF1, HOXA11, HOXA13
chr16	q12.2/q13/q21	56500001	34	15	3.76992e-15	HERPUD1
chr2	q35	219500001	47	18	5.49780e-15	FEV
chr17	p13.1	7000001	68	23	5.89050e-15	TP53
chr11	q13.1	64500001	56	20	8.24670e-15	MEN1
chr8	p11.21	41500001	12	9	1.02102e-14	KAT6A

For each significant genomic region the chromosome (chr), chromosome band (locus), nucleotide base start site of the genomic region (start), number of genes of the gene set (size), number of edges of the significant subnetwork (edges), Bonferroni adjusted p-value of the subnetwork (p-value) and a list of genes in the significant subnetwork that are present in the cancer census (census).

**Table 4:** **GPEA analysis of 1 Mb genomic regions gene sets for the Oligo urothelial cancer GRN**

**chr**	**Locus**	**Start**	**Size**	**Edges**	**p-value**	**Census**
chr1	q23.3	161000001	36	27	4.82850e-30	FCGR2B, SDHC
chr17	q25.3	79500001	47	30	5.15040e-28	ASPSCR1
chr1	q21.3	150500001	35	25	1.80264e-27	MLLT11, ARNT
chr11	p15.5	1	50	25	8.36940e-20	HRAS
chr8	q24.3	145000001	43	22	8.04750e-19	RECQL4
chr1	p34.3/p34.2	40000001	23	15	6.11610e-18	MYCL1
chr1	q23.3	160500001	34	18	3.86280e-17	SDHC
chr19	q13.2/q13.31	43000001	20	13	4.18470e-16	
chr9	p21.1/p13.3	32500001	17	12	4.50660e-16	
chr16	p13.2	8000001	7	8	1.41636e-15	
chr6	p21.1	42500001	33	16	1.25541e-14	
chr1	q21.2/q21.3	150000001	27	14	3.54090e-14	ARNT
chr8	p11.21	42000001	17	11	4.18470e-14	
chr16	p13.2	8500001	9	8	2.06016e-13	
chr16	q21/q22.1	66500001	42	17	1.31979e-12	CBFB
chr17	q12/q21.1/q21.2	37500001	31	14	1.67388e-12	ERBB2, CDK12, RARA
chr4	q13.3	74500001	14	9	5.47230e-12	
chr6	p21.1	43000001	26	12	1.80264e-11	
chr1	q21.3/q22	154500001	42	16	2.44644e-11	MUC1
chr4	q13.3	74000001	16	9	7.40370e-11	
chr8	p11.23/p11.22	37500001	20	10	9.65700e-11	FGFR1, WHSC1L1
chr11	q13.2	67000001	36	14	1.03008e-10	
chr9	p21.3	20500001	24	11	1.06227e-10	MLLT3
chr1	p34.2	40500001	22	10	6.75990e-10	
chr11	p11.2	47000001	22	10	6.75990e-10	DDB2
chr13	q34	113500001	18	9	6.75990e-10	
chr2	p22.1	39000001	11	7	1.22322e-09	
chr13	q14.2	48500001	11	7	1.22322e-09	RB1
chr19	p13.12	15000001	27	11	1.41636e-09	BRD4
chr17	q25.3	80000001	23	10	1.67388e-09	
chr17	p13.3	500001	19	9	1.83483e-09	YWHAE
chr1	p34.3	37500001	20	9	4.82850e-09	
chr1	q22	155000001	42	14	6.75990e-09	MUC1
chr9	p21.3	21000001	25	10	9.01320e-09	CDKN2A
chr3	p25.3	9500001	34	12	1.06227e-08	VHL
chr16	q12.2/q13/q21	56500001	34	12	1.06227e-08	HERPUD1
chr19	q13.33/q13.41	51000001	49	15	3.21900e-08	KLK2
chr1	p34.3	38000001	18	8	3.86280e-08	
chr11	q13.1/q13.2	65000001	46	14	7.40370e-08	
chr11	q13.1/q13.2	65500001	46	14	7.40370e-08	
chr19	q13.31	44000001	37	12	7.72560e-08	
chr20	p13	3000001	28	10	8.69130e-08	
chr6	p21.32/p21.31	32500001	42	13	9.97890e-08	DAXX
chr12	q15	69000001	15	7	1.31979e-07	MDM2
chr20	q11.22/q11.23	33500001	24	9	1.35198e-07	
chr1	q21.3	153000001	43	13	1.80264e-07	
chr9	p21.1/p13.3	33000001	20	8	2.12454e-07	
chr22	q11.21	20500001	20	8	2.12454e-07	
chr8	q24.3	144500001	39	12	2.60739e-07	
chr11	q13.2	66500001	35	11	3.86280e-07	

For each significant genomic region the chromosome (chr), chromosome band (locus), nucleotide base start site of the genomic region (start), number of genes of the gene set (size), number of edges of the significant subnetwork (edges), Bonferroni adjusted p-value of the subnetwork (p-value) and a list of genes in the significant subnetwork that are present in the cancer census (census).

### Gene pair enrichment analysis of gene family subnetworks

A gene family is a group of duplicated genes with similar biological functions or biochemical activities which often form gene clusters of genes with chromosomal co-location. In this section we performed a GPEA for gene family gene sets and compared the results between the UC RNAseq, Bead and Oligo GRNs.

We found a total of 20.44*%* (103) significant gene family subnetworks in the RNAseq GRN (Additional file 1: Table S7), but only 8.2*%* (40) for the Bead UC GRN (Additional file 1: Table S8) and 9.1*%* (39) for the Oligo UC GRN were significantly enriched (Additional file 1: Table S9). However, we found a high agreement of the gene family subnetworks between our GRNs with a total pairwise overlap of 30−60*%* and among the three networks 25*%* of all identified gene family subnetworks (Figure 1D). It is noteworthy that 97.5*%* of the gene families identified by the Bead and 82*%* of the gene families identified by the Oligo network were also significantly enriched in the RNAseq GRN. In total 28 gene families were identified across all three UC GRNs which described CD molecules, keratin proteins (KRT), protocadherins (PCDHC), kalikrein proteins (KLK), zinc-finger transcription factors, metallothioneins and the immunoglobulin superfamily. An overview of the significant gene families that were common across all three GRNs are shown in Table 5.

**Table 5:** **GPEA analysis of gene family gene sets for the UC RNAseq, Bead and Oligo GRN**

**Tag**	**Name**	**RNAseq**	**Bead**	**Oligo**
CD	CD molecules	380/591/0	365/319/1.1e-140	351/319/8.9e-106
ZKRAB	Zinc fingers, C2H2-type with KRAB domain	338/764/0	306/289/5.3e-155	143/108/5.6e-64
RPL	L ribosomal proteins	59/119/1.2e-175	55/49/2.2e-59	44/72/3.4e-102
ZNF	Zinc fingers, C2H2-type	697/1307/0	641/515/4e-114	362/242/2.3e-53
HIST	Histones/Replication-dependent	67/236/0	61/154/5.7e-252	26/32/1.3e-48
HLA	Histocompatibility complex	24/46/1.1e-85	25/33/2.9e-57	19/22/9.1e-36
C1SET	Immunoglobulin superfamily/C1-set domain containing	38/51/1e-75	38/34/1.5e-46	37/26/1.1e-28
KLK	Kallikreins	17/26/1.2e-49	16/15/2.8e-26	14/14/1.7e-23
MT	Metallothioneins	14/12/3.5e-20	12/12/7.1e-23	10/10/3.5e-18
PCDHC	Cadherins/Protocadherins : Clustered	57/150/7.8e-242	56/24/6.6e-21	22/14/1.3e-17
IGD	Immunoglobulin superfamily/Immunoglobulin-like domain containing	233/175/3.6e-85	221/77/5.1e-22	177/67/2.6e-17
KRT	Keratins	55/87/1.4e-121	51/20/2.8e-17	35/20/1.5e-20
HOXL	Homeoboxes/ANTP class : HOXL subclass	52/54/1.6e-66	50/39/1.8e-46	43/20/5.2e-17
COLLAGEN	Collagens	46/37/3.4e-43	43/21/1.1e-21	33/17/5.5e-17
RPS	S ribosomal proteins	34/40/7.2e-59	32/14/3.1e-15	29/28/6.1e-38
PSM	Proteasome (prosome, macropain) subunits	45/25/4.1e-25	43/15/5e-13	42/26/7.7e-26
RBM	RNA binding motif (RRM) containing	209/114/2.6e-46	186/50/1.6e-12	151/72/2.3e-28
ENDOLIG	Endogenous ligands	230/77/7.9e-16	221/62/3.3e-13	192/62/2.8e-11
VSET	Immunoglobulin superfamily/V-set domain containing	161/95/1.4e-50	150/42/5.2e-14	110/33/9e-11
comI	Mitochondrial respiratory chain complex/Complex I	38/14/3.9e-12	38/12/2.6e-10	31/15/7.3e-15
UGT	UDP glucuronosyltransferases	20/15/4.8e-22	20/17/2.3e-27	7/5/1.4e-08
IFF2	Intermediate filaments type II, keratins (basic)	26/23/2.6e-33	24/8/3e-08	15/7/1.8e-08
comIV	Mitochondrial respiratory chain complex/Complex IV	16/13/8.5e-21	15/6/1.3e-07	12/7/6.3e-10
S100	S100 calcium binding proteins	21/12/1.1e-15	21/7/1.8e-07	17/7/1.1e-07
LNCRNA	Long non-coding RNAs	548/407/1.2e-78	468/168/2e-14	107/25/5.4e-06
complement	Complement system	35/18/3.8e-19	33/7/1e-04	30/9/9.6e-07
CYP	Cytochrome P450s	62/37/1.1e-33	56/10/0.00019	48/22/1.2e-17
SERPIN	Serine (or cysteine) peptidase inhibitors	36/21/2.1e-23	35/8/1.2e-05	30/7/0.00031

Shown are the commonly significant gene family subnetworks of the RNAseq, Bead and Oligo UC GRN, number of genes of the gene family subnetwork, the number of interactions and the Bonferroni adjusted P-value (Genes/Interactions/P-value).

### Bladder cancer GRN degree centrality and hub genes

The identification of highly interactive central genes, i.e., hub genes of inferred and experimental network can provide promising targets for urothelial cancer. In this section we described individual hub genes of the gene regulatory network and review their functional role and relevance for the study of UC.

In order to compare the global structural properties of individual genes we performed a pairwise comparison of the degree centrality for 11,700 genes that are present among the three networks. The pairwise comparisons of the gene degree centrality across the three networks showed only a weak correlation. The degree ranks showed a slightly higher correlation between the RNAseq and Bead GRN of *r*=0.22 (RNAseq-Bead, *p*≤2.2*e*−16) compared to *r*=0.16 (Bead-Oligo, *p*≤2.2*e*−16) and *r*=0.16 (RNAseq-Oligo, *p*≤2.2*e*−16).

Hub genes of gene regulatory networks were observed to be highly dataset specific. Table 6 A, B, C shows the six most frequently observed hub genes for each of the inferred UC GRNs. In the following we describe the hub genes for which there is strong evidence for their relevance to cancer related properties. For example, the transmembrane protein *HID1* that was observed as a major hubgene in the RNAseq GRN is reported to be downregulated in multiple cancers [53]; *FER1L4* is a lncRNA reported to be prominently downregulated in gastric cancer [54], *TTLL3* is described as a candidate cancer gene [55], RIF1 has been described to have anti-apoptotic properties in DNA repair [56] and *SBNO1* (strawberry notch homolog 1) was reported in lung cancer [57]. For the UC Bead GRN, *RNF17 (TDRD4)* is a potential liver cancer CT antigen [58] and *TMED7* was observed to be upregulated in a nasopharyngeal carcinoma cell line and described to act as a major *immune system switch* [59]. The Oligo GRN hubgene *CYP4A11* was shown to promote angiogenesis and metastasis in lung cancer [60] and *SLC38A3 (SNAT3)* is a glutamine transporter and has been described as a marker for malignant glioma [61].

**Table 6:** **The six major hub genes for the RNAseq, Bead and Oligo UC GRN**

**A) RNAseq UC GRN**						
**Gene**	**Degree**	**Locus**	**Description/GO**	**Cancer association**	**Cancer**	**Ref**
HID1 (DMC1)	139	chr17q25.1	Transmembrane	Downregulated	Breast, cervix, liver, lung,	[53]
					thyroid, stomach, kidney	
FER1L4	135	20q11.22	lncRNA	Downregulated	Stomach	[54,81]
TTLL3	128	3p25.3	Tubulin-tyrosine	Downregulated	Colon	[55,82]
			ligase activity			
RIF1	126	2q23.3	DNA repair	Anti-apoptotic	Breast	[56]
KLHDC7A (FLJ38753)	116	1p36.13	Transmembrane	-	-	-
SBNO1	110	12q24.31	DNA binding	Proliferation	Lung	[57]
**B) Bead UC GRN**						
**Gene**	**Degree**	**Locus**	**Description/GO**	**Cancer association**	**Cancer**	**Ref**
MGC15885	119	15q22.2	ncRNA	-	-	-
RNF17 (TDRD4)	107	13q12.12	Spermatid development	Potential cancer CT antigen	Liver	[58]
OR6S1	105	14q11.2	G-protein coupled	-	-	-
			receptor signaling pathway			
ACTR3BP5	104	10p11.1	Pseudogene	-	-	-
TPTE2P3	98	13q14.3	Pseudogene	-	-	-
TMED7	90	5q22.3	Protein transport	Upregulated	Nasopharynx carcinoma	[59]
					cell line	
**C) Oligo UC GRN**						
**Gene**	**Degree**	**Locus**	**Description/GO**	**Cancer association**	**Cancer**	**Ref**
CYP4A11	184	1p33	Monooxygenase activity	Promotes angiogenesis	Lung	[60]
				and metastasis		
GJC2	162	1q42.13	Gap junction channel	-	-	-
			activity			
GPATCH4	147	1q23.1	Nucleic acid binding	-	-	-
ADAM5	141	8p11.22	Metalloendopeptidase	-	-	-
			activity, pseudogene			
DKFZP434A062	120	9q34.3	Uncharacterized protein	-	-	-
SLC38A3 (SNAT3)	118	3p21.31	Symporter activity	malignancy marker	Glioma	[61]

Shown are the gene symbols of the hub genes of the (A) RNAseq, (B) Bead and (C) Oligo UC GRN, their number of interactors (Degree), chromosomal location (Locus), functional description when available from GO or gene description, literature-based evidence or property for a cancer association, cancer types (Cancer) and literature citation (Ref) when available.

Overlapped only for a single term, i.e., for the *regulation of neuron differentiation (GO:0045664)*. For the RNAseq and Oligo GRN we observed nine terms in agreement, e.g., *G-protein coupled receptor signaling pathway (GO:0007186)*, *ion transport (GO:0006811)* and *sensory perception (GO:0007600)*. We observed that the average degree centrality of the networks with randomized gene labels was similar across the gene sets and independent of the number of genes of a gene set. However, our RNAseq dataset mostly considered muscle-invasive UCs and shows terms associated to invasiveness. The Bead data comprised terms that were nuclear while the terms predicted from the Oligo data were predominantly extracellular membrane associated.

### Quantitative comparison of experimental interactions in RNAseq, Bead and Oligo UC GRN

In this section we quantified the extend of local and globally shared interactions between the inferred GRNs and ppi networks. We assessed the global extent of interactions from PPI databases that were present in the UC GRNs by comparing the entire GRNs networks to String, Biogrid, Hprd, Intact, Mint, Graphite, CPDB and SingNet (Additional file 1: Table S10).

As observed on the subnetwork level using all String interactions the F-scores between the RNAseq and the Oligo UC GRN were similar. In contrast the Bead UC GRN showed a lower F-score for all PPI databases compared to the RNA-seq GRN and Bead GRN (Additional file 1: Table S10).

**Figure 4:**
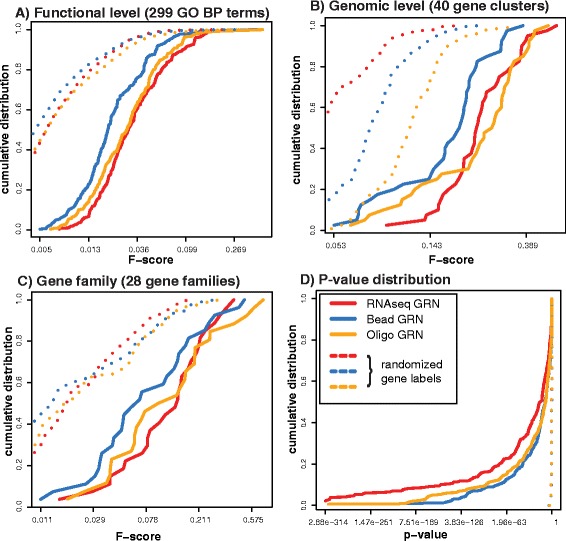
Network inference performance using interactions from the STRING database. Shown are the F-score distributions for commonly significant GRN subnetworks of the GPEA analysis for **A)** 299 Gene Ontology Biological Process terms; **B)** co-located genes of 1 Mb genomic regions; **C)** 28 gene families; and **D)** p-value distribution (FDR) estimated for all gene sets.

Further, we compared the relative quantity of PPI interactions for the identified significant subnetworks of the gene sets for biological processes, genomic co-located genes and gene families. To avoid the comparison to subnetworks with no known protein-protein associations we used String as reference as it was the largest collection of PPI interactions that we considered in our study. For each gene set we computed F-scores by comparing the corresponding subnetwork of the GRN to the subnetwork of the String network reference. Figure 4 shows the cumulative F-score distributions between the RNAseq, Bead and Oligo GRN separately for the 299 GO terms, 28 gene family subnetworks and 40 chromosomal 1 Mb regions. In addition, we repeated the analysis for each GRN 25 times using a reference subnetwork where the gene labels were randomized (Figure 4).

The average F-scores were highest for the subnetworks of genomic co-located genes (*F*_*RNAseq*_=0.26, *F*_*Bead*_=0.18 and *F*_*Oligo*_=0.26) and for the gene family gene sets (*F*_*RNAseq*_=0.15, *F*_*Bead*_=0.12, *F*_*Oligo*_=0.15). The Gene Ontology gene sets had the lowest average F-scores compared to the genomic and gene family subnetworks (*F*_*RNAseq*_=0.044, *F*_*Bead*_=0.029, *F*_*Oligo*_=0.039) (Figure 4 and Additional file 1: Table S11). The observations are in agreement with the global analysis for RNAseq and Oligo GRN, where the Bead UC GRN has the tendency to perform worse. However, the RNAseq UC GRN shows the tendency for smaller p-values over the Bead and Oligo UC GRN (Figure 4D).

The reference network with randomized gene labels were significantly lower compared to the GRN for all comparisons. The Bead GRN had the tendency to show a significantly lower mean F-score compared to the RNAseq and Oligo GRN for the Gene Ontology (t-test, *p*_*RNAseq*−*Bead*_=0.000007, *p*_*Bead*−*Oligo*_=0.002390) and the genomic 1Mb window subnetworks (*p*_*RNAseq*−*Bead*_=0.000675, *p*_*Bead*−*Oligo*_=0.001697). For the gene family subnetworks we did not observe a significant difference among the three GRN.

## Discussion

In this paper, we have presented novel perspectives and applications for the identification of UC molecular targets using GRNs. Specifically, we performed a structural, functional and comparative analysis across three UC GRNs that were independently inferred from three large-scale RNAseq, Bead and Oligo gene expression datasets. Our objective was to identify putative prognostic UC targets for a subsequent investigation in UC tumors on the basis of their enrichment in functional subnetworks and hub gene analysis. Our results demonstrate that GRNs are highly dataset specific on the gene interaction level and showed large similarities across the functional subnetwork levels. The RNAseq based GRN showed the most prominent functional enrichment and is thus the data type of choice for a network inference. The RNAseq and Oligo GRN showed a similar inference performance based on public interaction databases and outperformed the Bead based GRN.

On the structural level, the three inferred GRNs were observed to follow a power law distribution [62] that is common for inferred and experimental biological networks [63-65]. Our results demonstrated that the network structure at the gene level of GRNs are highly dependent on the individual gene expression dataset. On the gene interaction level the pairwise comparison between the networks showed only an overlap of 2*%* and only 0.25*%* of all interactions are common among the three networks (Figure 1). There are three main explanations for this observation. The first reason is that the BC3Net algorithm considers only the strongest interaction neighbors for each gene and is thus highly dependent on the search space of the genes that are included in the dataset. The second reason is that the variations caused by concordance differences of the expression are dependent on technical properties of the individual gene expression platforms and platform dependent data processing procedures. The third reason is that the datasets represent varying proportions of different tumor grades and stages from individual patients that represent a complex condition phenotype. Further, gene expression profiles of tumor tissues are highly heterogeneous on the molecular and tissue level, i.e., tumor clonal variation within and between different patient samples [66].

In [67] a guilt-by-association approach was developped to predict molecular roles of genes with unknown functions. The “guilt-by-association” property of genes that are connected in a defined network can also be used for a functional enrichment analysis for gene pairs which have known functions and are involved in the same biological processes. We identified significant functional GRN subnetworks by performing a gene pair enrichment analysis (GPEA) for defined gene sets. We used the terminology gene pair enrichment analysis to distinguish the latter from the terminology for a gene-based enrichment analysis which has no structural constraint. The concept for the analysis was introduced from graph theory [68] and has been developped and applied for the identification of significant protein complex and ontology gene sets in PPI and inferred networks [69-72].

A total of 5 to 10*%* of all tested Gene Ontology Biological process terms, 2 to 10*%* of gene sets of co-located genes and 9 to 20*%* gene family gene sets showed a significant subnetworks by the enrichment of inferred interactions (Figure 1). RNAseq based network showed more than twice the proportion of significant subnetworks compared to the Bead and Oligo microarray based GRN. Our results showed in a quantative manner that RNAseq is beneficial for GRN inference compared to Bead and Oligo microarray based data. The major advantages of RNAseq are more accurate measurement of the dynamic range of low and highly expressed genes [73] and thus gives a better resolution of the underlying functional dependency structure between the genes.

In contrast to the low similarity that was observed between the GRNs on the structural interaction level, we observed high similarities on the functional subnetwork level (Figure 1). The fraction of significant biological process Gene Ontology terms that were common across the three UC GRN was above 55*%*. For the gene family subnetworks we observed a similarity for 25.45*%* and the lowest percentage for genomic co-located gene subnetworks 6.8*%* (Figure 1).

The networks described a prominent enrichment for known cancer hallmarks [74] with significant GO subnetworks related to immune response, cell cycle, signal transduction, DNA repair, translation, proteolysis, metabolic terms such as respiration and cell morphogenesis, adhesion and migration. Further, over 50*%* of the significant GO subnetworks were highly enriched by known cancer genes defined by the cosmic cancer census [31] across the three UC GRNs. We observed that the fraction of cancer associated subnetworks is prominently lower in relevance network inference methods. This may result from low dependency measures of relevant interactions of genes in a more complex context being excluded from a relevance network by a global threshold. For other GRN inference methods we expect similar results to the results presented by the BC3Net that is based on the C3Net. A C3Net infers a core structure of a GRN and thus infers only a subnetwork of other GRN inference methods based on mutual information [10]. For each gene in a C3Net at most one gene neighbor with strongest mutual dependency is selected, which results in a highly reduced time complexity for multiple testing of mutual information. The C3Net and BC3Net GRNs inference method is therefore less time consuming which makes the inference of very large GRNs (>20*K* genes) feasible in a reasonable time.

On the genomic level, the GRNs were investigated for genomic UC targets, where we identified genomic regions with known diagnostic and prognostic properties for urothelial cancer such as 1*q*23.3 [52] (Oligo GRN), 8*q*24.3 [51] (Bead GRN) and 5*q*31.3 [48-50] (RNAseq GRN). The identified genomic regions can link to chromosomal aberrations, histone modifications, changes in epigenetic regulation (methylation), regulatory elements and spatial chromosome organization in the nucleus. These processes are commonly deregulated in cancer. For example the impairment of DNA repair mechanisms leads to an accumulation of chromosomal aberrations that are frequently observed in the progression of UC [75]. The identification of subnetworks of genomic regions from co-located genes therefore provided a powerful tool to identify putative novel genomic targets from cancer gene expression datasets.

In the analysis of gene families we found CD molecules as the most prominent gene family. CD molecules are promising targets for novel cancer immunotherapies such as CD47 [76]. Some popular UC biomarkers target proteins of an entire gene family and not a single gene product such as Keratins [77] and Kalikrein proteins [78] which are popular tumor markers for UC. Gene families are crucial in cancer research [79] because they represent groups of genes that are functionally highly redundant and represent potential targets of the underlying molecular heterogeneity that is observed for malignant processes. We showed that the identification and ranking of functional and co-located gene sets and gene families using our GPEA on GRNs is a versatile approach for the generation of novel targets and molecular understanding of the properties of urothelial cancer from the perspective of large-scale tumor tissue gene expression data.

Hub genes of GRNs reflect the most prominent dependencies of the expression profile to a large number of genes. We identified hub genes such as *HID1* [53] (RNAseq) *RNF17 (TDRD4)* [58] (Bead), *CYP4A11* [60] (Oligo) for the individual GRNs which show in the literature strong evidence for cancer related diagnostic and prognostic properties. Further, we performed a degree centrality analysis of the GRNs that showed that the degree centrality of the genes allow to target promising mediators of cancer related cellular activities and signaling processes.

In addition, we performed a quantitative comparison of protein interactions for the RNAseq, Bead and Oligo UC GRNs. We note that the overlap for protein interaction data and GRNs is expected to be low and non-random. For example the most prominent PPI interactions that can be found in a GRNs are physical interactions of genes corresponding to large protein complexes (e.g. ribosome biogenesis and proteasome) in contrast to more transient protein interactions [11,80]. A GRN is inferred from gene expression data and thus can only detect indirect association to the protein level of a gene network. However, the analysis allowed to compare network properties between the Oligo, Bead and RNAseq data and pointed to the tendency that Oligo expression data should be prefered over Bead expression data for a GRN inference.

## Conclusion

On the functional and structural level our results demonstrated that RNAseq based data is the preferred data type for a GRN inference. GRNs are highly dataset-specific at the interaction level, while at the global functional level they are highly similar. GRN inference is a powerful tool to provide a database of novel UC targets that can be studied for prognostic and diagnostic clinical applications [48,49,52,58,60].

## Additional file

Additional file 1 **Supplementary Materials: Urothelial cancer gene regulatory networks inferred from large-scale RNAseq, Bead and Oligo gene expression data.**
