# Supplementary material for: Urothelial cancer gene regulatory networks inferred from large-scale RNAseq, Bead and Oligo gene expression data
Source: BMC Syst Biol. 2015 May 14;9:21. doi: 10.1186/s12918-015-0165-z (PMC4460634; doi:10.1186/s12918-015-0165-z)
Supplement: Supplementary file 1 — Supplementary Materials: Urothelial cancer gene regulatory networks inferred from large-scale RNAseq, Bead and Oligo gene expression data. [file 12918_2015_165_MOESM1_ESM.pdf]

Supplementary Materials: Urothelial cancer gene regulatory networks inferred from large-scale RNAseq, Bead and Oligo gene expression data

Ricardo de Matos Simoes[1]
Email: r.dematossimoes@qub.ac.uk

Sabine Dalleau[1]
Email: s.dalleau@qub.ac.uk

Kate E Williamson[1]
*Corresponding author
Email: k.williamson@qub.ac.uk

Frank Emmert-Streib[2,3]
*Corresponding author
Email: v@bio-complexity.com

[1]Centre for Cancer Research and Cell Biology (CCRCB), Queens University Belfast, 97 Lisburn Road, Belfast, County Antrim, Northern Ireland, UK

[2]Computational Medicine and Statistical Learning Laboratory, Department of Signal Processing, Tampere University of Technology, 33720 Tampere, Finland

[3]Institute of Biosciences and Medical Technology, 33520 Tampere, Finland

Overview of Supplementary Tables and Figures

Figure S1	Functional landscape of urothelial cancer gene regulatory networks.
Table S1	GO biological process GPEA analysis of the RNAseq UC GRN.
Table S2	GO biological process GPEA analysis of the Bead UC GRN.
Table S3	GO biological process GPEA analysis of the Oligo UC GRN.
Table S4	Chromosomal GPEA analysis of the RNAseq UC GRN.
Table S5	Chromosomal GPEA analysis of the Bead UC GRN.
Table S6	Chromosomal GPEA analysis of the Oligo UC GRN.
Table S7	GPEA analysis of the RNAseq UC GRN for gene family gene sets.
Table S8	GPEA analysis of the Bead UC GRN for gene family gene sets.
Table S9	GPEA analysis of the Oligo UC GRN for gene family gene sets.
Table S10	Global comparisons of the urothelial cancer RNAseq, Bead and Oligo UC GRN to experimental and inferential PPI databases.
Table S11	Pairwise comparison of the F-score distributions between the RNAseq, Bead and Oligo UC GRN.

Functional landscape of urothelial cancer gene regulatory networks

For a given set of gene ontology terms (e.g., set of significant terms) we drew a Gene Ontology graph using *drawGO* (unpublished). In the first step the procedure extracted the entire Gene Ontology graph [1] from the defined set of genes and its corresponding parental terms to the root term of the GO. The GO graph was then restructured by deleting parental terms that were not included in the defined set

and by retaining the indirect associations between the terms. The parental terms that were iteratively deleted when corresponding child terms of a deleted term were reconnected to the parental terms of the deleted term. The introduced association in the graph therefore do not necessarily represent direct parent child connections and can include more distant ancestor child connection. The layout in *drawGO* is based on a force-based grid layout [2] that is remapped to node coordinates of a DIN scaled grid plane to prevent overlap between the nodes. The color scheme of the terms represented the rank of a term in the input, with red corresponding to terms that were highly ranked and yellow to terms with a lower rank. Functionally related GO terms were subsequently manually assigned into encircled subgroups and labeled to provide an intuitive interpretation of the functional landscape.

References

1. Beissbarth T, Speed T.P.. GOstat: find statistically overrepresented Gene Ontologies within a group of genes. Bioinformatics 2004;20:1464–5.

2. Csardi G, Nepusz T. The igraph software package for complex network research. InterJournal 2006;Complex Systems:1695.

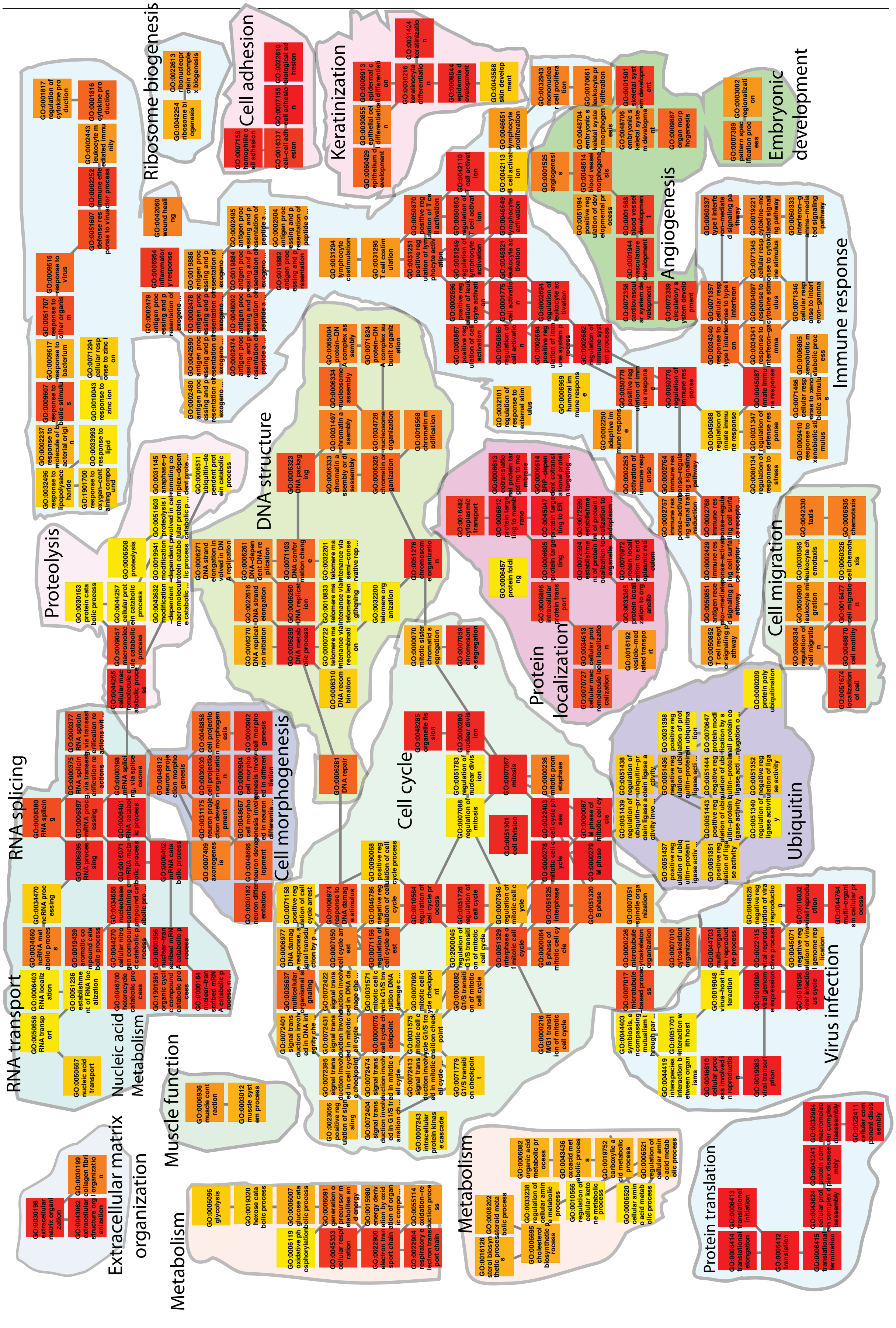

Figure S1 Functional landscape of urothelial cancer gene regulatory networks. Shown are 299 Gene Ontology biological process terms with common significantly enriched subnetworks among the RNAseq, Bead and Oligo UC GRNs. The ranks of the terms for the individual GRN were aggregated. The color range from red to yellow denotes highly to low ranking of the terms.

GPEA analysis of the RNAseq UC GRN for GO biological process

GOID	Term	edges	genes	gcc	padj	census
GO:0000184	nuclear-transcribed mRNA catabolic process, nonsense-mediated decay	301	118	76	0	4
GO:0006413	translational initiation	321	153	85	0	6
GO:0006414	translational elongation	320	107	81	0	3
GO:0006415	translational termination	302	91	76	0	3
GO:0006613	cotranslational protein targeting to membrane	305	107	76	0	4
GO:0006614	SRP-dependent cotranslational protein targeting to membrane	304	105	76	0	4
GO:0019080	viral genome expression	310	152	77	0	12/+
GO:0019083	viral transcription	310	152	77	0	12/+
GO:0045047	protein targeting to ER	306	107	76	0	4
GO:0070972	protein localization to endoplasmic reticulum	307	121	76	0	4
GO:0072599	establishment of protein localization to endoplasmic reticulum	306	108	76	0	4
GO:0006612	protein targeting to membrane	306	154	76	1.889e-316	6
GO:0043624	cellular protein complex disassembly	306	157	76	2.027e-311	5
GO:0043241	protein complex disassembly	306	162	76	2.842e-303	5
GO:0022403	cell cycle phase	1209	850	390	5.409e-300	70/+
GO:0000956	nuclear-transcribed mRNA catabolic process	311	171	79	3.634e-296	10
GO:0006402	mRNA catabolic process	313	183	80	4.368e-281	10
GO:0032984	macromolecular complex disassembly	311	183	78	1.91e-278	13/+
GO:0072594	establishment of protein localization to organelle	335	210	84	4.326e-272	6
GO:0000278	mitotic cell cycle	1042	773	351	6.789e-272	63/+
GO:0019058	viral infectious cycle	346	228	79	2.661e-262	17/+
GO:0006401	RNA catabolic process	321	210	81	5.29e-255	10
GO:0007155	cell adhesion	1239	958	598	1.126e-226	49/+
GO:0022610	biological adhesion	1241	960	608	3.962e-226	49/+
GO:0016071	mRNA metabolic process	730	608	307	1.31e-219	30
GO:0000279	M phase	615	535	200	2.641e-202	38/+
GO:0006412	translation	532	463	195	1.435e-201	18
GO:0022411	cellular component disassembly	344	293	81	3.654e-191	18
GO:0002682	regulation of immune system process	1002	856	439	1.63e-186	89/+
GO:0000087	M phase of mitotic cell cycle	407	373	135	4.939e-178	23/+
GO:0000280	nuclear division	391	362	130	8.151e-174	23/+
GO:0007067	mitosis	391	362	130	8.151e-174	23/+
GO:0048285	organelle fission	406	387	133	2.81e-166	23/+
GO:0045321	leukocyte activation	572	547	243	2.008e-165	68/+
GO:0001775	cell activation	803	751	359	1.665e-158	79/+
GO:0050776	regulation of immune response	516	528	257	1.603e-143	48/+
GO:0022415	viral reproductive process	535	546	141	1.785e-143	50/+
GO:0046649	lymphocyte activation	447	462	201	1.418e-142	65/+
GO:0002684	positive regulation of immune system process	510	528	231	7.14e-140	47/+
GO:0051301	cell division	448	479	142	2.691e-132	39/+
GO:0016032	viral reproduction	680	694	269	3.34e-132	53/+
GO:0044764	multi-organism cellular process	680	696	269	4.761e-131	53/+
GO:0007156	homophilic cell adhesion	156	139	51	9.721e-130	3
GO:0006259	DNA metabolic process	879	863	403	3.894e-127	84/+
GO:0042110	T cell activation	306	334	128	3.481e-126	51/+
GO:0008544	epidermis development	242	273	100	1.602e-114	24/+
GO:0030198	extracellular matrix organization	189	206	87	4.995e-112	11
GO:0006605	protein targeting	417	482	87	1.996e-111	40/+
GO:0043062	extracellular structure organization	189	207	87	2.615e-111	11
GO:0033365	protein localization to organelle	445	514	93	6.203e-110	47/+
GO:0051276	chromosome organization	629	699	338	2.239e-105	79/+
GO:0048610	cellular process involved in reproduction	467	548	80	8.757e-104	53/+
GO:0045087	innate immune response	433	513	229	1.055e-103	28/+
GO:0031424	keratinization	69	43	32	3.282e-102	0
GO:0051249	regulation of lymphocyte activation	239	290	100	3e-101	38/+
GO:0002694	regulation of leukocyte activation	269	331	113	5.633e-100	39/+
GO:0050865	regulation of cell activation	288	356	127	2.571e-99	42/+
GO:0044265	cellular macromolecule catabolic process	611	698	199	7.845e-98	41/+
GO:0006396	RNA processing	548	650	303	1.048e-92	26
GO:0044703	multi-organism reproductive process	634	733	152	4.569e-91	56/+
GO:0022904	respiratory electron transport chain	97	98	50	4.438e-89	5
GO:0050863	regulation of T cell activation	172	222	80	1.469e-85	31/+
GO:0022900	electron transport chain	116	136	58	5.873e-84	5
GO:0016337	cell-cell adhesion	307	409	97	9.162e-84	19
GO:0030216	keratinocyte differentiation	94	101	43	1.215e-82	3
GO:0002252	immune effector process	333	444	166	4.325e-82	33/+
GO:0072358	cardiovascular system development	647	767	346	4.555e-81	65/+
GO:0072359	circulatory system development	647	767	346	4.555e-81	65/+
GO:0016477	cell migration	740	848	436	9.196e-81	50/+
GO:0006886	intracellular protein transport	578	709	120	7.158e-79	53/+
GO:0009913	epidermal cell differentiation	102	121	45	5.48e-78	8
GO:0010564	regulation of cell cycle process	322	440	131	9.873e-78	49/+
GO:0006954	inflammatory response	359	488	213	6.394e-76	22
GO:0034655	nucleobase-containing compound catabolic process	634	769	167	2.624e-75	50/+
GO:0007059	chromosome segregation	114	146	51	3.917e-75	13/+
GO:0000904	cell morphogenesis involved in differentiation	559	700	314	4.665e-75	62/+
GO:0050867	positive regulation of cell activation	168	235	77	7.565e-75	29/+
GO:0016482	cytoplasmic transport	582	723	118	1.497e-74	56/+
GO:0045333	cellular respiration	115	150	61	1.011e-73	9
GO:0001944	vasculature development	384	522	211	1.382e-73	42/+
GO:0000902	cell morphogenesis	838	946	525	2.8e-73	71/+
GO:0048870	cell motility	804	921	472	2.04e-72	56/+
GO:0051674	localization of cell	804	921	472	2.04e-72	56/+
GO:0019882	antigen processing and presentation	150	212	66	7.738e-72	3
GO:0001501	skeletal system development	269	387	98	2.873e-71	38/+
GO:0051726	regulation of cell cycle	592	741	245	5.064e-71	87/+
GO:0002696	positive regulation of leukocyte activation	157	225	72	5.976e-71	28/+
GO:0001568	blood vessel development	356	497	193	1.889e-70	41/+
GO:0006397	mRNA processing	278	403	111	2.139e-69	20
GO:0019439	aromatic compound catabolic process	666	814	193	1.041e-68	50/+
GO:0050778	positive regulation of immune response	237	350	114	2.249e-68	31/+
GO:0008380	RNA splicing	220	328	97	1.948e-67	16
GO:0070727	cellular macromolecule localization	850	972	366	6.789e-67	68/+
GO:0034613	cellular protein localization	844	968	350	1.282e-66	68/+
GO:0006260	DNA replication	181	272	79	1.976e-66	26/+

GO:0030182	neuron differentiation	862	983	513	2.655e-66	79/+
GO:0044270	cellular nitrogen compound catabolic process	656	812	175	4.665e-66	50/+
GO:0046700	heterocycle catabolic process	653	812	175	4.854e-65	50/+
GO:0048002	antigen processing and presentation of peptide antigen	125	183	54	7.008e-65	3
GO:0007017	microtubule-based process	291	431	87	1.311e-64	29/+
GO:0051251	positive regulation of lymphocyte activation	136	207	68	2.193e-62	28/+
GO:0009057	macromolecule catabolic process	723	883	239	2.34e-61	52/+
GO:1901361	organic cyclic compound catabolic process	681	848	187	7.007e-61	50/+
GO:0051607	defense response to virus	120	184	53	7.84e-60	9
GO:0051707	response to other organism	382	555	185	3.03e-59	26
GO:0050870	positive regulation of T cell activation	108	164	59	7.584e-59	24/+
GO:0030030	cell projection organization	772	935	474	1.704e-57	66/+
GO:0006323	DNA packaging	97	146	56	2.077e-57	9
GO:0002478	antigen processing and presentation of exogenous peptide antigen	107	166	28	6.822e-57	3
GO:0019884	antigen processing and presentation of exogenous antigen	108	168	28	7.86e-57	3
GO:0051325	interphase	254	404	116	1.241e-55	39/+
GO:0051329	interphase of mitotic cell cycle	249	398	115	2.84e-55	38/+
GO:0002474	antigen processing and presentation of peptide antigen via MHC class I	71	99	37	7.608e-55	0
GO:0009607	response to biotic stimulus	395	582	188	9.581e-55	28
GO:0002768	immune response-regulating cell surface receptor signaling pathway	89	136	53	1.756e-54	17/+
GO:0000375	RNA splicing, via transesterification reactions	131	216	60	4.542e-54	12
GO:0006974	response to DNA damage stimulus	419	615	219	1.42e-52	66/+
GO:0000377	RNA splicing, via transesterification reactions with bulged adenosine as nucleophile	126	211	55	2.682e-52	12
GO:0000398	mRNA splicing, via spliceosome	126	211	55	2.682e-52	12
GO:0048858	cell projection morphogenesis	472	675	247	1.522e-51	54/+
GO:0060337	type I interferon-mediated signaling pathway	55	71	31	1.784e-51	5
GO:0071357	cellular response to type I interferon	55	71	31	1.784e-51	5
GO:0048666	neuron development	598	800	330	2.095e-51	65/+
GO:0034340	response to type I interferon	55	72	31	8.015e-51	5
GO:0006325	chromatin organization	340	529	126	1.325e-50	64/+
GO:0060429	epithelium development	404	604	215	1.388e-50	61/+
GO:0031175	neuron projection development	487	695	285	4.685e-50	60/+
GO:0048514	blood vessel morphogenesis	267	437	161	5.911e-50	33/+
GO:0000226	microtubule cytoskeleton organization	164	283	67	7.333e-50	24/+
GO:0007010	cytoskeleton organization	595	802	337	8.216e-50	55/+
GO:0000075	cell cycle checkpoint	143	249	86	4.19e-49	29/+
GO:0048812	neuron projection morphogenesis	370	570	182	7.65e-49	53/+
GO:0032990	cell part morphogenesis	475	687	248	1.435e-48	54/+
GO:0051320	S phase	89	148	56	1.465e-48	9
GO:0071103	DNA conformation change	106	182	57	1.592e-48	16/+
GO:0035637	multicellular organismal signaling	553	766	328	1.872e-48	28
GO:0048667	cell morphogenesis involved in neuron differentiation	364	565	181	3.858e-48	52/+
GO:0006334	nucleosome assembly	66	101	35	8.884e-48	5
GO:0002253	activation of immune response	160	282	78	1.213e-47	28/+
GO:0002429	immune response-activating cell surface receptor signaling pathway	77	125	37	1.332e-47	16/+
GO:0009887	organ morphogenesis	585	801	376	4.398e-47	86/+
GO:0002764	immune response-regulating signaling pathway	137	244	66	7.489e-47	29/+
GO:0050851	antigen receptor-mediated signaling pathway	73	118	34	7.817e-47	16/+
GO:0001816	cytokine production	251	425	146	2.147e-46	33/+
GO:0071156	regulation of cell cycle arrest	152	272	90	3.055e-46	31/+
GO:0009615	response to virus	145	263	57	5.102e-45	14
GO:0002479	antigen processing and presentation of exogenous peptide antigen via MHC class I, TAP-dependent	52	75	28	6.43e-45	0
GO:0002521	leukocyte differentiation	186	333	82	1.367e-44	63/+
GO:0000084	S phase of mitotic cell cycle	81	140	49	1.491e-44	8
GO:0006281	DNA repair	227	396	120	1.612e-44	45/+
GO:0034728	nucleosome organization	72	121	35	2.539e-44	11/+
GO:0042060	wound healing	387	604	222	3.619e-44	37/+
GO:0042590	antigen processing and presentation of exogenous peptide antigen via MHC class I	53	79	28	6.18e-44	0
GO:0031497	chromatin assembly	68	113	35	6.462e-44	9
GO:0006935	chemotaxis	380	598	223	1.703e-43	48/+
GO:0042330	taxis	380	598	223	1.703e-43	48/+
GO:0001525	angiogenesis	201	361	123	4.584e-43	26/+
GO:0006091	generation of precursor metabolites and energy	253	438	115	6.018e-43	20
GO:0019226	transmission of nerve impulse	513	744	308	7.056e-43	28
GO:0007409	axonogenesis	310	516	156	2.063e-42	51/+
GO:0030199	collagen fibril organization	33	36	17	3.127e-42	2
GO:0007268	synaptic transmission	425	654	257	5.856e-42	18
GO:0030855	epithelial cell differentiation	160	298	55	8.977e-42	30/+
GO:0002757	immune response-activating signal transduction	122	231	55	3.128e-41	28/+
GO:0000216	M/G1 transition of mitotic cell cycle	51	80	38	7.567e-41	0
GO:0030098	lymphocyte differentiation	118	228	50	1.618e-39	45/+
GO:0065004	protein-DNA complex assembly	71	130	35	2.916e-39	6
GO:0002520	immune system development	348	574	134	3.836e-39	101/+
GO:0030334	regulation of cell migration	216	396	139	4.288e-39	30/+
GO:0007050	cell cycle arrest	203	377	101	8.574e-39	44/+
GO:0030097	hemopoiesis	297	511	111	1.011e-38	90/+
GO:0007389	pattern specification process	238	430	51	1.204e-38	45/+
GO:0006333	chromatin assembly or disassembly	72	134	35	1.56e-38	15/+
GO:0051270	regulation of cellular component movement	263	468	153	7.359e-38	38/+
GO:0040012	regulation of locomotion	252	453	151	9.808e-38	36/+
GO:0034097	response to cytokine stimulus	284	498	142	1.912e-37	37/+
GO:0048534	hematopoietic or lymphoid organ development	319	544	121	2.446e-37	95/+
GO:0034341	response to interferon-gamma	60	110	36	8.509e-37	9
GO:0071824	protein-DNA complex subunit organization	77	150	35	1.175e-36	12/+
GO:0022616	DNA strand elongation	30	37	23	3.294e-36	2
GO:2000145	regulation of cell motility	225	419	141	3.755e-36	36/+
GO:0008284	positive regulation of cell proliferation	408	657	220	1.727e-35	63/+
GO:0000236	mitotic prometaphase	49	86	36	2.004e-35	5
GO:0032943	mononuclear cell proliferation	91	185	34	3.789e-35	27/+
GO:0048706	embryonic skeletal system development	60	114	12	4.308e-35	17/+
GO:0019221	cytokine-mediated signaling pathway	169	334	70	5.761e-35	30/+
GO:0006261	DNA-dependent DNA replication	54	100	31	9.296e-35	7
GO:0070661	leukocyte proliferation	94	193	35	1.335e-34	29/+
GO:0016568	chromatin modification	233	437	109	2.509e-34	61/+
GO:0034660	ncRNA metabolic process	143	291	36	3.923e-34	8
GO:0007599	hemostasis	275	498	161	6.814e-34	31/+
GO:0015980	energy derivation by oxidation of organic compounds	158	319	66	9.38e-34	16
GO:0050900	leukocyte migration	124	258	60	2.751e-33	19/+

GO:0055114	oxidation-reduction process	311	548	116	2.925e-33	18
GO:0006271	DNA strand elongation involved in DNA replication	27	34	20	3.867e-33	0
GO:0007596	blood coagulation	270	494	158	4.365e-33	30/+
GO:0030217	T cell differentiation	78	162	33	5.016e-33	34/+
GO:0001817	regulation of cytokine production	194	382	114	6.418e-33	29/+
GO:0046651	lymphocyte proliferation	87	183	32	8.786e-33	27/+
GO:0071345	cellular response to cytokine stimulus	213	413	94	1.426e-32	31/+
GO:0019886	antigen processing and presentation of exogenous peptide antigen via MHC class II	48	90	15	1.798e-32	3
GO:0050817	coagulation	270	497	158	2.992e-32	30/+
GO:0051094	positive regulation of developmental process	432	696	259	3.566e-32	75/+
GO:0045786	negative regulation of cell cycle	236	451	104	1.345e-31	52/+
GO:0022613	ribonucleoprotein complex biogenesis	109	234	23	1.97e-31	11
GO:0002250	adaptive immune response	87	187	50	1.98e-31	22/+
GO:0002495	antigen processing and presentation of peptide antigen via MHC class II	48	93	15	3.396e-31	3
GO:0050852	T cell receptor signaling pathway	48	93	17	3.396e-31	12/+
GO:0002504	antigen processing and presentation of peptide or polysaccharide antigen via MHC class II	48	94	15	8.822e-31	3
GO:0071346	cellular response to interferon-gamma	47	92	12	1.636e-30	9/+
GO:0048704	embryonic skeletal system morphogenesis	44	85	10	4.425e-30	12/+
GO:0007264	small GTPase mediated signal transduction	276	514	110	6.999e-30	25
GO:0003002	regionalization	147	313	22	1.007e-29	36/+
GO:0048729	tissue morphogenesis	253	483	131	1.378e-29	53/+
GO:0002697	regulation of immune effector process	98	217	43	1.552e-29	20/+
GO:0060333	interferon-gamma-mediated signaling pathway	39	73	12	2.28e-29	9/+
GO:0000082	G1/S transition of mitotic cell cycle	89	198	56	2.589e-29	16/+
GO:0060284	regulation of cell development	265	502	105	4.853e-29	51/+
GO:0031347	regulation of defense response	212	425	126	7.88e-29	29/+
GO:0009790	embryo development	644	923	375	2.455e-28	107/+
GO:0051960	regulation of nervous system development	224	446	120	3.949e-28	42/+
GO:0006936	muscle contraction	113	254	47	7.094e-28	6
GO:0050878	regulation of body fluid levels	327	590	178	1.377e-27	35/+
GO:0031295	T cell costimulation	35	66	11	1.696e-27	10/+
GO:0002443	leukocyte mediated immunity	85	195	47	1.913e-27	18/+
GO:0048598	embryonic morphogenesis	258	499	86	2.787e-27	54/+
GO:0031294	lymphocyte costimulation	35	67	11	4.657e-27	10/+
GO:0007417	central nervous system development	408	690	246	7.052e-27	69/+
GO:0007051	spindle organization	40	82	25	9.016e-27	7
GO:0016192	vesicle-mediated transport	603	892	396	1.149e-26	37
GO:0048705	skeletal system morphogenesis	78	182	14	1.933e-26	18/+
GO:0071466	cellular response to xenobiotic stimulus	64	148	20	2.531e-26	1
GO:0002460	adaptive immune response based on somatic recombination of immune receptors built from immunoglobulin superfamily domains	72	169	43	5.375e-26	18/+
GO:0070663	regulation of leukocyte proliferation	65	152	30	7.36e-26	21/+
GO:0006805	xenobiotic metabolic process	63	147	20	7.913e-26	1
GO:0009410	response to xenobiotic stimulus	64	150	20	1.092e-25	1
GO:0031581	hemidesmosome assembly	14	12	9	1.753e-25	0
GO:0042254	ribosome biogenesis	64	151	20	2.244e-25	6
GO:0035295	tube development	210	436	91	3.332e-25	48/+
GO:0022603	regulation of anatomical structure morphogenesis	329	603	173	4.807e-25	56/+
GO:0032944	regulation of mononuclear cell proliferation	62	147	29	5.109e-25	20/+
GO:0043436	oxoacid metabolic process	694	983	416	8.688e-25	29
GO:0016339	calcium-dependent cell-cell adhesion	20	28	12	8.99e-25	0
GO:0006082	organic acid metabolic process	713	1000	432	1.06e-24	29
GO:0048387	negative regulation of retinoic acid receptor signaling pathway	18	23	9	2.069e-24	2
GO:0009952	anterior/posterior pattern specification	87	211	15	3.935e-24	26/+
GO:0007420	brain development	252	505	119	9.946e-24	49/+
GO:0019752	carboxylic acid metabolic process	571	875	316	1.446e-23	22
GO:0030029	actin filament-based process	225	466	46	1.569e-23	31/+
GO:0031589	cell-substrate adhesion	90	222	39	5.226e-23	17/+
GO:0007411	axon guidance	158	359	84	7.813e-23	37/+
GO:0006695	cholesterol biosynthetic process	24	44	11	1.398e-22	1
GO:0042098	T cell proliferation	53	131	25	1.569e-22	18/+
GO:0006811	ion transport	698	997	476	1.75e-22	25
GO:0009617	response to bacterium	134	316	62	1.803e-22	13
GO:0050670	regulation of lymphocyte proliferation	58	146	27	3.877e-22	20/+
GO:0032946	positive regulation of mononuclear cell proliferation	41	98	21	4.794e-22	13/+
GO:0070665	positive regulation of leukocyte proliferation	42	101	22	4.865e-22	13/+
GO:0031145	anaphase-promoting complex-dependent proteasomal ubiquitin-dependent protein catabolic process	36	83	18	5.444e-22	2
GO:0003012	muscle system process	117	285	48	8.5e-22	8
GO:0048562	embryonic organ morphogenesis	88	223	13	1.52e-21	24/+
GO:0002480	antigen processing and presentation of exogenous peptide antigen via MHC class I, TAP-independent	11	9	7	1.808e-21	0
GO:0050671	positive regulation of lymphocyte proliferation	40	97	20	2.171e-21	13/+
GO:0034329	cell junction assembly	68	175	23	2.225e-21	11
GO:0043009	chordate embryonic development	275	548	60	2.564e-21	74/+
GO:0048385	regulation of retinoic acid receptor signaling pathway	18	28	9	3.145e-21	2
GO:0048585	negative regulation of response to stimulus	467	781	269	5.109e-21	69/+
GO:0002449	lymphocyte mediated immunity	59	153	27	6.433e-21	15/+
GO:0034330	cell junction organization	76	198	25	1.302e-20	13
GO:0009792	embryo development ending in birth or egg hatching	277	555	60	2.151e-20	74/+
GO:0030036	actin cytoskeleton organization	186	418	24	2.424e-20	29/+
GO:0080134	regulation of response to stress	421	734	239	2.751e-20	58/+
GO:0045664	regulation of neuron differentiation	133	324	47	4.142e-20	27/+
GO:0045597	positive regulation of cell differentiation	238	501	120	5.035e-20	62/+
GO:0016126	sterol biosynthetic process	24	50	11	5.783e-20	1
GO:0007606	sensory perception of chemical stimulus	43	111	8	7.272e-20	1
GO:0002009	morphogenesis of an epithelium	163	381	25	8.619e-20	42/+
GO:0040017	positive regulation of locomotion	92	241	37	2.286e-19	22/+
GO:0050767	regulation of neurogenesis	171	397	50	2.359e-19	42/+
GO:0007346	regulation of mitotic cell cycle	128	318	70	3.055e-19	33/+
GO:0051056	regulation of small GTPase mediated signal transduction	152	364	26	3.614e-19	25/+
GO:0032787	monocarboxylic acid metabolic process	188	427	64	4.24e-19	13
GO:0007044	cell-substrate junction assembly	26	59	15	4.485e-19	5
GO:0000819	sister chromatid segregation	24	53	18	8.739e-19	1
GO:0050911	detection of chemical stimulus involved in sensory perception of smell	18	33	8	1.273e-18	0
GO:0006007	glucose catabolic process	30	75	24	3.307e-18	1
GO:0006521	regulation of cellular amino acid metabolic process	25	58	17	3.336e-18	2
GO:0042129	regulation of T cell proliferation	39	105	22	5.13e-18	14/+

GO:0042102	positive regulation of T cell proliferation	28	69	12	5.893e-18	9/+
GO:0048384	retinoic acid receptor signaling pathway	19	38	9	6.723e-18	4
GO:0008203	cholesterol metabolic process	42	115	15	7.558e-18	3
GO:0001932	regulation of protein phosphorylation	485	816	286	8.595e-18	78/+
GO:0030335	positive regulation of cell migration	83	227	29	1.059e-17	20/+
GO:0006270	DNA replication initiation	16	29	12	2.745e-17	2
GO:0010648	negative regulation of cell communication	360	678	89	3.444e-17	63/+
GO:0008202	steroid metabolic process	102	273	17	3.448e-17	6
GO:0009968	negative regulation of signal transduction	331	642	83	3.489e-17	62/+
GO:2000147	positive regulation of cell motility	84	232	29	4.129e-17	22/+
GO:0016125	sterol metabolic process	43	121	15	4.569e-17	3
GO:0023057	negative regulation of signaling	357	675	89	4.624e-17	63/+
GO:0009967	positive regulation of signal transduction	483	818	235	5.3e-17	73/+
GO:0006968	cellular defense response	24	58	11	5.594e-17	3
GO:0016072	rRNA metabolic process	40	112	15	5.9e-17	3
GO:0072001	renal system development	89	245	17	7.446e-17	31/+
GO:0002683	negative regulation of immune system process	62	177	30	7.466e-17	20/+
GO:0006364	rRNA processing	38	106	15	7.601e-17	3
GO:0051272	positive regulation of cellular component movement	86	238	29	7.751e-17	24/+
GO:0001655	urogenital system development	106	284	28	1.059e-16	37/+
GO:0007423	sensory organ development	190	442	34	1.129e-16	40/+
GO:0006977	DNA damage response, signal transduction by p53 class mediator resulting in cell cycle arrest	26	66	18	1.135e-16	5
GO:0072413	signal transduction involved in mitotic cell cycle checkpoint	26	66	18	1.135e-16	5
GO:0072431	signal transduction involved in mitotic cell cycle G1/S transition DNA damage checkpoint	26	66	18	1.135e-16	5
GO:0072474	signal transduction involved in mitotic cell cycle G1/S checkpoint	26	66	18	1.135e-16	5
GO:0016570	histone modification	99	270	43	2.136e-16	37/+
GO:0051436	negative regulation of ubiquitin-protein ligase activity involved in mitotic cell cycle	26	67	18	2.366e-16	1
GO:0072401	signal transduction involved in DNA integrity checkpoint	26	67	18	2.366e-16	6
GO:0072404	signal transduction involved in G1/S transition checkpoint	26	67	18	2.366e-16	5
GO:0072422	signal transduction involved in DNA damage checkpoint	26	67	18	2.366e-16	6
GO:0023056	positive regulation of signaling	520	861	268	3.994e-16	74/+
GO:0016569	covalent chromatin modification	101	276	43	4.535e-16	40/+
GO:0072395	signal transduction involved in cell cycle checkpoint	26	68	18	4.869e-16	6
GO:0000070	mitotic sister chromatid segregation	21	50	17	5.255e-16	1
GO:0045071	negative regulation of viral genome replication	16	32	8	6.607e-16	1
GO:0006631	fatty acid metabolic process	112	301	37	7.793e-16	11
GO:0032101	regulation of response to external stimulus	152	380	83	8.155e-16	23/+
GO:0060485	mesenchyme development	56	165	13	9.493e-16	17/+
GO:0002237	response to molecule of bacterial origin	73	211	38	1.01e-15	10
GO:0061448	connective tissue development	69	201	20	1.216e-15	21/+
GO:0045619	regulation of lymphocyte differentiation	36	104	11	1.265e-15	19/+
GO:0010647	positive regulation of cell communication	519	863	266	1.429e-15	75/+
GO:0048525	negative regulation of viral reproduction	16	33	8	1.774e-15	1
GO:0007243	intracellular protein kinase cascade	534	879	293	2.018e-15	83/+
GO:0044255	cellular lipid metabolic process	488	832	202	2.228e-15	28
GO:0060271	cilium morphogenesis	36	106	14	4.188e-15	1
GO:0019320	hexose catabolic process	31	89	24	4.441e-15	1
GO:0033238	regulation of cellular amine metabolic process	27	75	18	5.08e-15	3
GO:0031570	DNA integrity checkpoint	47	142	18	6.502e-15	16/+
GO:0001503	ossification	102	284	25	7.055e-15	36/+
GO:0051352	negative regulation of ligase activity	26	72	18	7.729e-15	2
GO:0051437	positive regulation of ubiquitin-protein ligase activity involved in mitotic cell cycle	26	72	18	7.729e-15	1
GO:0051444	negative regulation of ubiquitin-protein ligase activity	26	72	18	7.729e-15	2
GO:0006096	glycolysis	23	61	18	8.119e-15	1
GO:0042325	regulation of phosphorylation	524	873	314	1.091e-14	84/+
GO:0045088	regulation of innate immune response	75	221	27	1.134e-14	20/+
GO:0051240	positive regulation of multicellular organismal process	236	525	107	1.246e-14	45/+
GO:0048568	embryonic organ development	130	344	16	1.27e-14	47/+
GO:0006310	DNA recombination	71	211	31	1.307e-14	26/+
GO:0007018	microtubule-based movement	57	173	20	1.387e-14	3
GO:0031571	mitotic cell cycle G1/S transition DNA damage checkpoint	26	73	18	1.499e-14	6
GO:0002819	regulation of adaptive immune response	31	91	18	1.523e-14	9/+
GO:0030155	regulation of cell adhesion	94	268	18	1.858e-14	30/+
GO:0034470	ncRNA processing	68	204	22	2.013e-14	5
GO:0043588	skin development	20	51	7	2.145e-14	8/+
GO:0034622	cellular macromolecular complex assembly	247	543	78	2.56e-14	28
GO:0007507	heart development	151	386	74	2.711e-14	42/+
GO:0007052	mitotic spindle organization	16	36	12	2.847e-14	3
GO:0051439	regulation of ubiquitin-protein ligase activity involved in mitotic cell cycle	27	78	18	3.538e-14	2
GO:0031575	mitotic cell cycle G1/S transition checkpoint	28	82	18	4.401e-14	9/+
GO:0002822	regulation of adaptive immune response based on somatic recombination of immune receptors built from immunoglobulin superfamily domains	28	83	17	8.128e-14	7
GO:0046365	monosaccharide catabolic process	31	94	24	9.077e-14	1
GO:0050907	detection of chemical stimulus involved in sensory perception	21	57	8	1.024e-13	0
GO:0071158	positive regulation of cell cycle arrest	27	80	18	1.227e-13	8/+
GO:0007126	meiosis	51	160	25	1.362e-13	15/+
GO:0051327	M phase of meiotic cell cycle	51	160	25	1.362e-13	15/+
GO:0042113	B cell activation	54	169	33	1.433e-13	34/+
GO:0007416	synapse assembly	25	73	11	1.638e-13	2
GO:0007517	muscle organ development	126	342	66	1.774e-13	26/+
GO:0007167	enzyme linked receptor protein signaling pathway	524	880	260	1.789e-13	86/+
GO:0051321	meiotic cell cycle	52	164	26	2.244e-13	15/+
GO:0051443	positive regulation of ubiquitin-protein ligase activity	27	81	18	2.252e-13	2
GO:1901700	response to oxygen-containing compound	409	757	199	2.468e-13	44/+
GO:0007160	cell-matrix adhesion	47	149	16	2.482e-13	13/+
GO:0045621	positive regulation of lymphocyte differentiation	22	63	8	4.277e-13	11/+
GO:0006812	cation transport	315	644	162	4.763e-13	17
GO:0071779	G1/S transition checkpoint	28	86	18	4.848e-13	10/+
GO:0010528	regulation of transposition	7	7	5	5e-13	0
GO:0010529	negative regulation of transposition	7	7	5	5e-13	0
GO:0002274	myeloid leukocyte activation	37	118	14	5.164e-13	8
GO:0000077	DNA damage checkpoint	42	135	18	6.353e-13	16/+
GO:0009100	glycoprotein metabolic process	135	363	20	6.577e-13	14
GO:0007506	gonadal mesoderm development	6	5	4	9.929e-13	0
GO:0002526	acute inflammatory response	34	109	11	1.053e-12	7
GO:0030326	embryonic limb morphogenesis	35	113	7	1.379e-12	23/+
GO:0035113	embryonic appendage morphogenesis	35	113	7	1.379e-12	23/+

GO:0072376	protein activation cascade	21	61	7	1.502e-12	0
GO:0046631	alpha-beta T cell activation	28	88	12	1.527e-12	17/+
GO:0007186	G-protein coupled receptor signaling pathway	271	588	40	1.85e-12	10
GO:0060326	cell chemotaxis	46	150	20	1.893e-12	9
GO:0051351	positive regulation of ligase activity	27	85	18	2.333e-12	2
GO:0061061	muscle structure development	188	462	99	2.488e-12	44/+
GO:0007093	mitotic cell cycle checkpoint	43	141	19	2.503e-12	15/+
GO:0050808	synapse organization	43	141	11	2.503e-12	8
GO:0035239	tube morphogenesis	104	302	12	2.803e-12	34/+
GO:0010627	regulation of intracellular protein kinase cascade	356	701	118	3.504e-12	70/+
GO:0007586	digestion	35	115	6	3.883e-12	0
GO:0048878	chemical homeostasis	509	873	293	4.341e-12	43/+
GO:0051253	negative regulation of RNA metabolic process	485	848	285	4.369e-12	97/+
GO:0001822	kidney development	63	202	10	4.407e-12	24/+
GO:0071294	cellular response to zinc ion	8	11	6	4.672e-12	0
GO:0071363	cellular response to growth factor stimulus	170	433	58	5.254e-12	51/+
GO:0030900	forebrain development	94	281	18	5.666e-12	37/+
GO:0002263	cell activation involved in immune response	40	133	11	6.186e-12	17/+
GO:0002366	leukocyte activation involved in immune response	40	133	11	6.186e-12	17/+
GO:0090068	positive regulation of cell cycle process	50	165	30	6.419e-12	15/+
GO:0006959	humoral immune response	31	102	8	7.517e-12	10/+
GO:0030168	platelet activation	68	217	19	7.978e-12	14
GO:0010558	negative regulation of macromolecule biosynthetic process	597	962	386	8.82e-12	103/+
GO:0051438	regulation of ubiquitin-protein ligase activity	29	95	18	9.18e-12	4
GO:0051216	cartilage development	50	166	18	1.017e-11	17/+
GO:0045069	regulation of viral genome replication	17	48	8	1.347e-11	4
GO:0006694	steroid biosynthetic process	41	138	11	1.384e-11	3
GO:0070848	response to growth factor stimulus	176	446	60	1.414e-11	53/+
GO:2000113	negative regulation of cellular macromolecule biosynthetic process	554	922	335	1.644e-11	102/+
GO:0045580	regulation of T cell differentiation	25	81	7	1.834e-11	16/+
GO:0032940	secretion by cell	345	692	87	1.98e-11	23
GO:0007608	sensory perception of smell	19	57	8	2.069e-11	1
GO:0032496	response to lipopolysaccharide	61	200	33	2.142e-11	8
GO:0031397	negative regulation of protein ubiquitination	29	97	19	2.623e-11	5
GO:0002706	regulation of lymphocyte mediated immunity	23	74	17	3.065e-11	6
GO:0035107	appendage morphogenesis	39	133	7	3.105e-11	24/+
GO:0035108	limb morphogenesis	39	133	7	3.105e-11	24/+
GO:0045892	negative regulation of transcription, DNA-dependent	451	817	258	3.334e-11	95/+
GO:0045934	negative regulation of nucleobase-containing compound metabolic process	558	928	340	3.458e-11	107/+
GO:0032196	transposition	7	9	5	3.547e-11	0
GO:0030595	leukocyte chemotaxis	36	123	18	3.624e-11	7
GO:0050657	nucleic acid transport	38	130	9	3.765e-11	10
GO:0050658	RNA transport	38	130	9	3.765e-11	10
GO:0051236	establishment of RNA localization	38	130	9	3.765e-11	10
GO:0043583	ear development	57	190	10	4.041e-11	23/+
GO:0043901	negative regulation of multi-organism process	18	54	9	4.363e-11	1
GO:0050864	regulation of B cell activation	25	83	11	5.445e-11	15/+
GO:0048762	mesenchymal cell differentiation	41	141	8	5.628e-11	14/+
GO:0006986	response to unfolded protein	38	131	8	6.027e-11	8
GO:0043900	regulation of multi-organism process	64	211	15	6.522e-11	18/+
GO:1901701	cellular response to oxygen-containing compound	143	390	55	6.56e-11	25/+
GO:0051340	regulation of ligase activity	29	99	18	7.285e-11	4
GO:0048736	appendage development	41	142	7	8.892e-11	24/+
GO:0060173	limb development	41	142	7	8.892e-11	24/+
GO:0033993	response to lipid	229	537	92	9.469e-11	40/+
GO:0046903	secretion	424	790	119	9.767e-11	27
GO:0060562	epithelial tube morphogenesis	94	288	12	9.772e-11	32/+
GO:0051172	negative regulation of nitrogen compound metabolic process	568	941	346	1.079e-10	107/+
GO:0000723	telomere maintenance	20	64	11	1.135e-10	9/+
GO:0001763	morphogenesis of a branching structure	59	199	11	1.583e-10	29/+
GO:0010833	telomere maintenance via telomere lengthening	14	39	10	1.94e-10	3
GO:0032200	telomere organization	20	65	11	2.012e-10	9/+
GO:0050906	detection of stimulus involved in sensory perception	26	90	8	2.667e-10	3
GO:0042384	cilium assembly	23	78	11	2.72e-10	1
GO:0035966	response to topologically incorrect protein	39	138	8	3.083e-10	8
GO:0007610	behavior	214	517	87	3.113e-10	25
GO:0046578	regulation of Ras protein signal transduction	87	275	11	4.051e-10	20/+
GO:0019079	viral genome replication	19	62	8	4.088e-10	6
GO:0007600	sensory perception	201	497	58	4.186e-10	10
GO:0042742	defense response to bacterium	33	118	10	5.313e-10	2
GO:0045859	regulation of protein kinase activity	275	609	41	5.582e-10	61/+
GO:0007178	transmembrane receptor protein serine/threonine kinase signaling pathway	84	269	23	6.761e-10	22/+
GO:0052548	regulation of endopeptidase activity	76	249	31	7.179e-10	19/+
GO:0000086	G2/M transition of mitotic cell cycle	41	147	23	8.128e-10	15/+
GO:2000045	regulation of G1/S transition of mitotic cell cycle	29	104	18	8.315e-10	10/+
GO:0070647	protein modification by small protein conjugation or removal	299	643	39	8.428e-10	40/+
GO:0006403	RNA localization	38	137	9	9.08e-10	10
GO:0014031	mesenchymal cell development	36	130	5	9.142e-10	12/+
GO:0072331	signal transduction by p53 class mediator	36	130	20	9.142e-10	17/+
GO:0000165	MAPK cascade	221	531	45	9.212e-10	55/+
GO:0051028	mRNA transport	31	112	7	1.01e-09	8
GO:0030001	metal ion transport	210	514	42	1.026e-09	16
GO:0007229	integrin-mediated signaling pathway	20	68	6	1.052e-09	2
GO:0043687	post-translational protein modification	55	192	12	1.097e-09	2
GO:0006520	cellular amino acid metabolic process	177	460	70	1.339e-09	9
GO:0055082	cellular chemical homeostasis	315	666	123	1.585e-09	30
GO:0032201	telomere maintenance via semi-conservative replication	10	24	9	1.61e-09	0
GO:0009101	glycoprotein biosynthetic process	103	316	17	1.666e-09	12
GO:0044057	regulation of system process	170	449	59	2.075e-09	22
GO:0022604	regulation of cell morphogenesis	97	303	12	2.116e-09	18
GO:0052547	regulation of peptidase activity	78	257	32	2.159e-09	19/+
GO:0050727	regulation of inflammatory response	54	191	15	2.441e-09	12
GO:0061138	morphogenesis of a branching epithelium	53	188	11	2.462e-09	28/+
GO:0033043	regulation of organelle organization	202	504	47	2.798e-09	50/+
GO:0052695	cellular glucuronidation	7	12	6	3.23e-09	0
GO:0031398	positive regulation of protein ubiquitination	36	133	18	3.368e-09	7
GO:0005975	carbohydrate metabolic process	399	772	226	3.836e-09	24
GO:0042770	signal transduction in response to DNA damage	32	119	19	4.345e-09	16/+
GO:0019048	virus-host interaction	127	370	32	4.616e-09	38/+

GO:0000910	cytokinesis	29	108	16	5.2e-09	8
GO:0030330	DNA damage response, signal transduction by p53 class mediator	29	108	19	5.2e-09	14/+
GO:0015931	nucleobase-containing compound transport	41	152	9	6.603e-09	10
GO:0043632	modification-dependent macromolecule catabolic process	138	393	29	6.753e-09	19
GO:0008285	negative regulation of cell proliferation	212	523	23	7.674e-09	68/+
GO:0002703	regulation of leukocyte mediated immunity	25	93	18	7.855e-09	8
GO:0000722	telomere maintenance via recombination	10	26	9	8.122e-09	0
GO:0051093	negative regulation of developmental process	229	550	39	9.156e-09	73/+
GO:0006063	uronic acid metabolic process	7	13	6	1.085e-08	0
GO:0019585	glucuronate metabolic process	7	13	6	1.085e-08	0
GO:0006457	protein folding	61	216	12	1.357e-08	3
GO:0006508	proteolysis	549	937	213	1.531e-08	29
GO:0019941	modification-dependent protein catabolic process	135	390	29	1.837e-08	19
GO:0071219	cellular response to molecule of bacterial origin	26	99	17	1.878e-08	3
GO:0042274	ribosomal small subunit biogenesis	8	18	6	2.191e-08	0
GO:0009593	detection of chemical stimulus	23	87	8	2.273e-08	0
GO:0006120	mitochondrial electron transport, NADH to ubiquinone	12	37	7	2.333e-08	0
GO:0006022	aminoglycan metabolic process	42	159	10	2.791e-08	7
GO:0071495	cellular response to endogenous stimulus	352	722	123	3.094e-08	69/+
GO:0001649	osteoblast differentiation	37	142	8	3.175e-08	20/+
GO:0005996	monosaccharide metabolic process	81	272	40	3.225e-08	6
GO:0006873	cellular ion homeostasis	265	607	98	3.337e-08	29
GO:0051701	interaction with host	144	410	44	4.25e-08	38/+
GO:0042391	regulation of membrane potential	81	273	38	4.585e-08	19/+
GO:0030203	glycosaminoglycan metabolic process	39	150	10	4.623e-08	7
GO:0006119	oxidative phosphorylation	17	62	7	4.78e-08	3
GO:0060537	muscle tissue development	101	321	45	4.912e-08	29/+
GO:0044403	symbiosis, encompassing mutualism through parasitism	162	444	48	4.972e-08	41/+
GO:0044419	interspecies interaction between organisms	162	444	48	4.972e-08	41/+
GO:0016055	Wnt receptor signaling pathway	94	305	13	5.219e-08	32/+
GO:0043408	regulation of MAPK cascade	172	462	27	5.227e-08	49/+
GO:0045582	positive regulation of T cell differentiation	15	53	7	5.86e-08	10/+
GO:0010951	negative regulation of endopeptidase activity	33	129	13	5.979e-08	6
GO:0010629	negative regulation of gene expression	515	907	291	6.021e-08	101/+
GO:0003013	circulatory system process	112	346	21	6.057e-08	9
GO:0001667	ameboidal cell migration	28	110	8	6.695e-08	4
GO:0006023	aminoglycan biosynthetic process	26	102	10	6.896e-08	7
GO:0019322	pentose biosynthetic process	4	4	4	8.451e-08	0
GO:0008015	blood circulation	111	345	21	8.97e-08	9
GO:0042773	ATP synthesis coupled electron transport	14	49	7	9.015e-08	2
GO:0042775	mitochondrial ATP synthesis coupled electron transport	14	49	7	9.015e-08	2
GO:0060678	dichotomous subdivision of terminal units involved in ureteric bud branching	5	7	4	1.04e-07	0
GO:0006006	glucose metabolic process	57	210	30	1.075e-07	6
GO:0001819	positive regulation of cytokine production	54	201	9	1.077e-07	15/+
GO:0051603	proteolysis involved in cellular protein catabolic process	143	411	29	1.115e-07	19
GO:0046364	monosaccharide biosynthetic process	19	73	12	1.151e-07	2
GO:0019724	B cell mediated immunity	24	95	9	1.204e-07	10/+
GO:0045765	regulation of angiogenesis	40	156	23	1.217e-07	12/+
GO:0034308	primary alcohol metabolic process	8	20	4	1.247e-07	1
GO:0044724	single-organism carbohydrate catabolic process	33	131	24	1.321e-07	1
GO:0006069	ethanol oxidation	6	11	4	1.323e-07	1
GO:0009812	flavonoid metabolic process	6	11	6	1.323e-07	0
GO:0006511	ubiquitin-dependent protein catabolic process	129	384	27	1.366e-07	19
GO:0034976	response to endoplasmic reticulum stress	28	112	9	1.523e-07	7
GO:0000209	protein polyubiquitination	43	167	21	1.619e-07	7
GO:0010563	negative regulation of phosphorus metabolic process	73	256	10	1.637e-07	28/+
GO:0045936	negative regulation of phosphate metabolic process	73	256	10	1.637e-07	28/+
GO:0070085	glycosylation	73	256	14	1.637e-07	6
GO:0043549	regulation of kinase activity	285	640	41	1.648e-07	66/+
GO:0045165	cell fate commitment	61	223	8	1.71e-07	42/+
GO:0006790	sulfur compound metabolic process	63	229	12	1.887e-07	10
GO:0010466	negative regulation of peptidase activity	33	132	13	1.949e-07	6
GO:0051130	positive regulation of cellular component organization	187	492	56	1.985e-07	46/+
GO:0045944	positive regulation of transcription from RNA polymerase II promoter	336	708	74	2.069e-07	105/+
GO:0018212	peptidyl-tyrosine modification	54	203	8	2.199e-07	30/+
GO:0055123	digestive system development	28	113	18	2.278e-07	15/+
GO:0002695	negative regulation of leukocyte activation	27	109	15	2.312e-07	16/+
GO:0086091	regulation of heart rate by cardiac conduction	7	16	4	2.363e-07	0
GO:0006369	termination of RNA polymerase II transcription	13	46	7	2.524e-07	4
GO:0006024	glycosaminoglycan biosynthetic process	25	101	10	2.556e-07	7
GO:2000602	regulation of interphase of mitotic cell cycle	40	158	18	2.568e-07	18/+
GO:0006312	mitotic recombination	11	36	9	2.603e-07	2
GO:0060548	negative regulation of cell death	272	624	75	3.135e-07	74/+
GO:0006302	double-strand break repair	28	114	6	3.389e-07	16/+
GO:0050866	negative regulation of cell activation	29	118	15	3.401e-07	18/+
GO:0006486	protein glycosylation	71	253	14	3.626e-07	6
GO:0043413	macromolecule glycosylation	71	253	14	3.626e-07	6
GO:0010740	positive regulation of intracellular protein kinase cascade	188	496	41	4.1e-07	45/+
GO:0051336	regulation of hydrolase activity	368	750	157	4.231e-07	53/+
GO:0044257	cellular protein catabolic process	148	425	29	4.639e-07	20
GO:0071222	cellular response to lipopolysaccharide	23	94	17	4.751e-07	3
GO:0048895	lateral line nerve glial cell differentiation	5	8	4	4.996e-07	0
GO:0048937	lateral line nerve glial cell development	5	8	4	4.996e-07	0
GO:0050935	iridophore differentiation	5	8	4	4.996e-07	0
GO:0086069	bundle of His cell to Purkinje myocyte communication	5	8	4	4.996e-07	0
GO:0043066	negative regulation of apoptotic process	250	594	70	5.114e-07	69/+
GO:0031400	negative regulation of protein modification process	97	319	33	5.37e-07	30/+
GO:0050801	ion homeostasis	313	682	119	6.319e-07	31
GO:0043069	negative regulation of programmed cell death	255	602	70	6.329e-07	72/+
GO:0070507	regulation of microtubule cytoskeleton organization	19	77	15	6.889e-07	8/+
GO:0031396	regulation of protein ubiquitination	46	181	19	6.914e-07	14/+
GO:0048193	Golgi vesicle transport	46	181	11	6.914e-07	7
GO:0034220	ion transmembrane transport	87	296	8	7.028e-07	7
GO:1901615	organic hydroxy compound metabolic process	126	383	29	7.036e-07	9
GO:0006418	tRNA aminoacylation for protein translation	13	48	7	7.184e-07	2
GO:0046634	regulation of alpha-beta T cell activation	15	58	8	7.189e-07	11/+
GO:0043086	negative regulation of catalytic activity	201	520	36	8.651e-07	32/+
GO:0016052	carbohydrate catabolic process	33	136	24	8.823e-07	1
GO:0007565	female pregnancy	39	158	13	9.14e-07	6

GO:0010043	response to zinc ion	10	33	7	9.276e-07	1
GO:0018108	peptidyl-tyrosine phosphorylation	52	201	7	9.284e-07	30/+
GO:0060541	respiratory system development	43	172	25	9.772e-07	23/+
GO:0051338	regulation of transferase activity	296	661	41	1.03e-06	69/+
GO:0086001	regulation of cardiac muscle cell action potential	9	28	5	1.064e-06	0
GO:0050792	regulation of viral reproduction	27	113	10	1.113e-06	12/+
GO:1901342	regulation of vasculature development	42	169	24	1.124e-06	15/+
GO:0071216	cellular response to biotic stimulus	26	109	17	1.171e-06	5
GO:0051552	flavone metabolic process	4	5	5	1.18e-06	0
GO:0070383	DNA cytosine deamination	4	5	4	1.18e-06	0
GO:0051084	’de novo’ posttranslational protein folding	13	49	5	1.189e-06	1
GO:0006353	DNA-dependent transcription, termination	20	83	7	1.248e-06	6
GO:0016064	immunoglobulin mediated immune response	22	92	8	1.25e-06	9/+
GO:0030031	cell projection assembly	55	212	11	1.695e-06	7
GO:0050871	positive regulation of B cell activation	14	55	7	1.846e-06	10/+
GO:0048892	lateral line nerve development	5	9	4	1.905e-06	1
GO:0048925	lateral line system development	5	9	4	1.905e-06	1
GO:0001657	ureteric bud development	24	102	4	2.06e-06	18/+
GO:0006066	alcohol metabolic process	92	312	17	2.439e-06	6
GO:0030323	respiratory tube development	37	154	21	2.766e-06	19/+
GO:0019725	cellular homeostasis	361	748	133	2.898e-06	32
GO:0048701	embryonic cranial skeleton morphogenesis	10	35	5	2.926e-06	5/+
GO:0071260	cellular response to mechanical stimulus	14	56	7	2.937e-06	5
GO:0006067	ethanol metabolic process	6	14	4	2.957e-06	1
GO:0043038	amino acid activation	13	51	7	3.143e-06	2
GO:0043039	tRNA aminoacylation	13	51	7	3.143e-06	2
GO:0044723	single-organism carbohydrate metabolic process	226	564	73	3.152e-06	13
GO:0044283	small molecule biosynthetic process	159	452	28	3.383e-06	9
GO:0051656	establishment of organelle localization	27	116	8	3.425e-06	6
GO:2000242	negative regulation of reproductive process	19	81	8	3.663e-06	6
GO:0071229	cellular response to acid	11	41	5	4.115e-06	6/+
GO:0014706	striated muscle tissue development	90	309	39	4.255e-06	29/+
GO:0048565	digestive tract development	24	104	16	4.403e-06	15/+
GO:0044711	single-organism biosynthetic process	165	464	28	4.684e-06	9
GO:0016266	O-glycan processing	13	52	5	5.024e-06	1
GO:0048839	inner ear development	39	163	8	5.171e-06	22/+
GO:0043087	regulation of GTPase activity	56	219	8	6.172e-06	15/+
GO:0022618	ribonucleoprotein complex assembly	24	105	4	6.387e-06	7
GO:0086036	regulation of cardiac muscle cell membrane potential	9	31	5	6.506e-06	0
GO:0045596	negative regulation of cell differentiation	154	445	11	6.538e-06	67/+
GO:0006690	icosanoid metabolic process	16	68	5	6.781e-06	2
GO:1901568	fatty acid derivative metabolic process	16	68	5	6.781e-06	2
GO:0010562	positive regulation of phosphorus metabolic process	294	665	95	7.209e-06	58/+
GO:0045937	positive regulation of phosphate metabolic process	294	665	95	7.209e-06	58/+
GO:0018279	protein N-linked glycosylation via asparagine	21	92	12	7.259e-06	1
GO:0002483	antigen processing and presentation of endogenous peptide antigen	4	6	5	7.639e-06	0
GO:0019885	antigen processing and presentation of endogenous peptide antigen via MHC class I	4	6	5	7.639e-06	0
GO:0045869	negative regulation of retroviral genome replication	4	6	4	7.639e-06	1
GO:0030278	regulation of ossification	37	157	6	7.695e-06	21/+
GO:0019318	hexose metabolic process	65	246	30	7.872e-06	6
GO:0030593	neutrophil chemotaxis	13	53	5	7.945e-06	3
GO:0031344	regulation of cell projection organization	70	260	14	8.266e-06	17/+
GO:0043161	proteasomal ubiquitin-dependent protein catabolic process	58	226	20	8.498e-06	10
GO:0006984	ER-nucleus signaling pathway	22	97	5	8.679e-06	6
GO:0042326	negative regulation of phosphorylation	54	214	8	8.716e-06	25/+
GO:0030163	protein catabolic process	201	528	29	9.14e-06	29/+
GO:0006633	fatty acid biosynthetic process	30	131	6	9.43e-06	6
GO:0018196	peptidyl-asparagine modification	21	93	12	1.059e-05	1
GO:0033121	regulation of purine nucleotide catabolic process	65	247	10	1.072e-05	15
GO:0010769	regulation of cell morphogenesis involved in differentiation	45	186	8	1.122e-05	13
GO:0009118	regulation of nucleoside metabolic process	66	250	10	1.137e-05	16
GO:0009954	proximal/distal pattern formation	9	32	3	1.139e-05	7/+
GO:0033124	regulation of GTP catabolic process	56	221	8	1.159e-05	15/+
GO:0006352	DNA-dependent transcription, initiation	58	227	14	1.161e-05	16/+
GO:0031349	positive regulation of defense response	58	227	12	1.161e-05	17/+
GO:0030968	endoplasmic reticulum unfolded protein response	19	84	5	1.194e-05	4
GO:0030522	intracellular receptor mediated signaling pathway	60	233	11	1.198e-05	29/+
GO:0030324	lung development	35	151	20	1.214e-05	19/+
GO:0006458	’de novo’ protein folding	13	54	5	1.243e-05	1
GO:0051248	negative regulation of protein metabolic process	172	480	40	1.297e-05	43/+
GO:0007283	spermatogenesis	126	393	21	1.435e-05	22
GO:0008610	lipid biosynthetic process	220	560	31	1.447e-05	18
GO:0001934	positive regulation of protein phosphorylation	231	577	70	1.447e-05	54/+
GO:0030811	regulation of nucleotide catabolic process	65	248	10	1.456e-05	15
GO:0009698	phenylpropanoid metabolic process	6	16	6	1.584e-05	0
GO:0022612	gland morphogenesis	25	112	7	1.68e-05	15/+
GO:0042100	B cell proliferation	14	60	9	1.709e-05	12/+
GO:0034370	triglyceride-rich lipoprotein particle remodeling	5	11	4	1.732e-05	0
GO:0034372	very-low-density lipoprotein particle remodeling	5	11	4	1.732e-05	0
GO:0010975	regulation of neuron projection development	52	210	13	1.737e-05	14
GO:0034620	cellular response to unfolded protein	19	85	5	1.748e-05	4
GO:0016053	organic acid biosynthetic process	82	294	11	1.784e-05	7
GO:0046394	carboxylic acid biosynthetic process	82	294	11	1.784e-05	7
GO:0006956	complement activation	11	44	5	1.801e-05	0
GO:0032269	negative regulation of cellular protein metabolic process	138	418	39	1.851e-05	35/+
GO:0051640	organelle localization	41	174	9	1.899e-05	12
GO:0048232	male gamete generation	126	394	21	1.913e-05	22
GO:0051052	regulation of DNA metabolic process	50	204	13	1.916e-05	32/+
GO:0035967	cellular response to topologically incorrect protein	20	90	5	1.942e-05	4
GO:0051250	negative regulation of lymphocyte activation	21	95	8	2.216e-05	15/+
GO:0006897	endocytosis	111	363	13	2.392e-05	19
GO:0044092	negative regulation of molecular function	298	675	119	2.582e-05	46/+
GO:0002440	production of molecular mediator of immune response	24	109	10	2.683e-05	17/+
GO:0042327	positive regulation of phosphorylation	238	590	84	2.803e-05	55/+
GO:0030513	positive regulation of BMP signaling pathway	8	28	4	2.831e-05	2
GO:0003158	endothelium development	13	56	4	2.956e-05	6
GO:0048732	gland development	74	275	28	2.965e-05	45/+
GO:0040007	growth	372	770	160	3.201e-05	73/+

GO:0090092	regulation of transmembrane receptor protein serine/threonine kinase signaling pathway	38	165	9	3.207e-05	16/+
GO:0000122	negative regulation of transcription from RNA polymerase II promoter	185	506	27	3.415e-05	67/+
GO:0009154	purine ribonucleotide catabolic process	143	430	21	3.493e-05	30/+
GO:0045216	cell-cell junction organization	39	169	6	3.647e-05	13/+
GO:0007224	smoothened signaling pathway	19	87	7	3.676e-05	9/+
GO:0006987	activation of signaling protein activity involved in unfolded protein response	14	62	5	3.904e-05	2
GO:0042180	cellular ketone metabolic process	55	222	19	3.987e-05	12
GO:0032886	regulation of microtubule-based process	20	92	16	4.027e-05	9/+
GO:0006184	GTP catabolic process	99	338	15	4.19e-05	20
GO:0034367	macromolecular complex remodeling	7	23	5	4.323e-05	0
GO:0034368	protein-lipid complex remodeling	7	23	5	4.323e-05	0
GO:0034369	plasma lipoprotein particle remodeling	7	23	5	4.323e-05	0
GO:0008360	regulation of cell shape	23	106	5	4.394e-05	5
GO:0048880	sensory system development	5	12	4	4.413e-05	1
GO:0086005	regulation of ventricular cardiac muscle cell action potential	5	12	4	4.413e-05	0
GO:0010720	positive regulation of cell development	35	155	9	4.46e-05	20/+
GO:0009261	ribonucleotide catabolic process	143	431	21	4.611e-05	30/+
GO:0006958	complement activation, classical pathway	8	29	4	4.927e-05	0
GO:0046039	GTP metabolic process	104	350	19	4.934e-05	20
GO:0002377	immunoglobulin production	16	73	8	4.982e-05	12/+
GO:0010565	regulation of cellular ketone metabolic process	39	170	18	4.986e-05	10
GO:0046165	alcohol biosynthetic process	29	132	11	5.102e-05	2
GO:0002709	regulation of T cell mediated immunity	9	35	6	5.469e-05	3
GO:0033205	cell cycle cytokinesis	9	35	8	5.469e-05	4
GO:0071230	cellular response to amino acid stimulus	9	35	5	5.469e-05	5/+
GO:0006487	protein N-linked glycosylation	21	98	12	6.434e-05	1
GO:0045667	regulation of osteoblast differentiation	21	98	5	6.434e-05	15/+
GO:0009894	regulation of catabolic process	190	517	26	6.811e-05	34/+
GO:0033559	unsaturated fatty acid metabolic process	17	79	5	7.099e-05	2
GO:0010498	proteasomal protein catabolic process	59	236	20	7.141e-05	10
GO:1901069	guanosine-containing compound catabolic process	99	340	15	7.356e-05	20
GO:0071826	ribonucleoprotein complex subunit organization	24	112	4	7.471e-05	8
GO:0060021	palate development	15	69	5	7.741e-05	7/+
GO:0055085	transmembrane transport	306	690	40	8.153e-05	17
GO:0050853	B cell receptor signaling pathway	8	30	4	8.396e-05	6/+
GO:0061337	cardiac conduction	8	30	4	8.396e-05	0
GO:0031401	positive regulation of protein modification process	343	738	96	8.561e-05	63/+
GO:0032075	positive regulation of nuclease activity	14	64	5	8.629e-05	2
GO:0002455	humoral immune response mediated by circulating immunoglobulin	10	42	4	9.672e-05	1
GO:0002456	T cell mediated immunity	11	48	7	0.0001082	4
GO:0050909	sensory perception of taste	11	48	6	0.0001082	0
GO:0006216	cytidine catabolic process	4	8	4	0.0001135	0
GO:0009972	cytidine deamination	4	8	4	0.0001135	0
GO:0019883	antigen processing and presentation of endogenous antigen	4	8	5	0.0001135	1
GO:0045091	regulation of retroviral genome replication	4	8	4	0.0001135	1
GO:0046087	cytidine metabolic process	4	8	4	0.0001135	0
GO:1901617	organic hydroxy compound biosynthetic process	35	158	11	0.0001137	3
GO:0015672	monovalent inorganic cation transport	76	285	10	0.0001148	4
GO:0034754	cellular hormone metabolic process	20	95	5	0.0001153	2
GO:1901137	carbohydrate derivative biosynthetic process	280	656	35	0.0001158	23
GO:0010942	positive regulation of cell death	148	444	40	0.0001167	45/+
GO:0007169	transmembrane receptor protein tyrosine kinase signaling pathway	258	625	81	0.0001218	64/+
GO:0009753	response to jasmonic acid stimulus	3	4	3	0.0001228	0
GO:0071395	cellular response to jasmonic acid stimulus	3	4	3	0.0001228	0
GO:0031329	regulation of cellular catabolic process	150	448	26	0.0001233	26/+
GO:0072524	pyridine-containing compound metabolic process	12	54	6	0.0001251	1
GO:0015985	energy coupled proton transport, down electrochemical gradient	6	19	3	0.0001324	0
GO:0015986	ATP synthesis coupled proton transport	6	19	3	0.0001324	0
GO:0086002	regulation of cardiac muscle cell action potential involved in contraction	6	19	4	0.0001324	0
GO:0032446	protein modification by small protein conjugation	225	576	36	0.0001342	36/+
GO:1901136	carbohydrate derivative catabolic process	214	559	47	0.0001431	34/+
GO:0051493	regulation of cytoskeleton organization	69	267	17	0.0001446	19/+
GO:0032318	regulation of Ras GTPase activity	35	159	5	0.0001541	12/+
GO:0006304	DNA modification	15	71	5	0.0001635	7
GO:0019362	pyridine nucleotide metabolic process	11	49	6	0.0001647	1
GO:0046496	nicotinamide nucleotide metabolic process	11	49	6	0.0001647	1
GO:0003001	generation of a signal involved in cell-cell signaling	99	343	21	0.0001678	14
GO:0023061	signal release	99	343	21	0.0001678	14
GO:0042089	cytokine biosynthetic process	21	101	5	0.0001781	9/+
GO:0007163	establishment or maintenance of cell polarity	24	115	4	0.0001994	6
GO:0042476	odontogenesis	22	106	4	0.000201	13/+
GO:0043542	endothelial cell migration	22	106	5	0.000201	9
GO:0048754	branching morphogenesis of an epithelial tube	35	160	6	0.0002082	23/+
GO:0050663	cytokine secretion	19	92	6	0.0002129	6
GO:0043065	positive regulation of apoptotic process	135	421	30	0.0002148	43/+
GO:0006195	purine nucleotide catabolic process	159	467	23	0.0002222	32/+
GO:1901068	guanosine-containing compound metabolic process	105	358	19	0.0002383	20
GO:0007265	Ras protein signal transduction	63	252	8	0.0002444	18/+
GO:0035023	regulation of Rho protein signal transduction	37	168	5	0.0002449	12
GO:0050832	defense response to fungus	6	20	3	0.0002477	0
GO:0051303	establishment of chromosome localization	6	20	5	0.0002477	2
GO:0032963	collagen metabolic process	12	56	6	0.0002764	3
GO:0033002	muscle cell proliferation	22	107	6	0.0002779	13/+
GO:0043068	positive regulation of programmed cell death	137	426	30	0.0002816	44/+
GO:0021953	central nervous system neuron differentiation	26	125	7	0.0002973	16/+
GO:0007088	regulation of mitosis	19	93	14	0.0002976	11/+
GO:0051783	regulation of nuclear division	19	93	14	0.0002976	11/+
GO:0044259	multicellular organismal macromolecule metabolic process	13	62	6	0.0003201	3
GO:0034587	piRNA metabolic process	4	9	5	0.0003246	0
GO:0045006	DNA deamination	4	9	4	0.0003246	0
GO:0046133	pyrimidine ribonucleoside catabolic process	4	9	4	0.0003246	0
GO:0009306	protein secretion	37	169	10	0.0003283	9
GO:0030888	regulation of B cell proliferation	11	51	6	0.0003702	10/+
GO:0019319	hexose biosynthetic process	14	68	8	0.0003842	2
GO:0032479	regulation of type I interferon production	14	68	5	0.0003842	3
GO:0072523	purine-containing compound catabolic process	160	471	23	0.000389	32/+
GO:0016051	carbohydrate biosynthetic process	36	166	14	0.0004034	7
GO:0002027	regulation of heart rate	12	57	4	0.0004054	0

GO:0045766	positive regulation of angiogenesis	18	89	5	0.0004056	5
GO:0071559	response to transforming growth factor beta stimulus	45	198	16	0.000415	17/+
GO:0050000	chromosome localization	6	21	5	0.0004479	2
GO:0006406	mRNA export from nucleus	13	63	6	0.0004627	5
GO:1901565	organonitrogen compound catabolic process	333	732	88	0.0004673	35
GO:0042107	cytokine metabolic process	21	104	5	0.0004712	9/+
GO:0018193	peptidyl-amino acid modification	276	656	64	0.0004801	59/+
GO:0060070	canonical Wnt receptor signaling pathway	41	185	9	0.0005176	24/+
GO:0044706	multi-multicellular organism process	43	192	14	0.0005222	7
GO:0030509	BMP signaling pathway	22	109	6	0.0005237	8
GO:0016042	lipid catabolic process	61	249	12	0.000526	3
GO:0002275	myeloid cell activation involved in immune response	10	46	5	0.0005323	3
GO:0097285	cell-type specific apoptotic process	68	269	7	0.000533	31/+
GO:0006493	protein O-linked glycosylation	14	69	5	0.0005481	1
GO:0009725	response to hormone stimulus	272	651	49	0.0005603	57/+
GO:0032480	negative regulation of type I interferon production	8	34	3	0.0005886	2
GO:0009143	nucleoside triphosphate catabolic process	130	415	17	0.0006139	30/+
GO:1900542	regulation of purine nucleotide metabolic process	99	348	14	0.0006303	18
GO:0001818	negative regulation of cytokine production	28	136	5	0.0006372	9
GO:0009203	ribonucleoside triphosphate catabolic process	126	407	17	0.0006429	30/+
GO:0009207	purine ribonucleoside triphosphate catabolic process	126	407	17	0.0006429	30/+
GO:0043269	regulation of ion transport	71	278	18	0.0006506	12
GO:0006094	gluconeogenesis	13	64	8	0.0006637	2
GO:0021675	nerve development	13	64	4	0.0006637	6
GO:0006739	NADP metabolic process	7	28	6	0.0006685	1
GO:0071804	cellular potassium ion transport	7	28	4	0.0006685	0
GO:0071805	potassium ion transmembrane transport	7	28	4	0.0006685	0
GO:0010901	regulation of very-low-density lipoprotein particle remodeling	3	5	3	0.0007349	0
GO:0052696	flavonoid glucuronidation	3	5	3	0.0007349	0
GO:0052697	xenobiotic glucuronidation	3	5	3	0.0007349	0
GO:0032069	regulation of nuclease activity	14	70	5	0.0007766	3
GO:0042035	regulation of cytokine biosynthetic process	18	91	4	0.0007788	9/+
GO:0043085	positive regulation of catalytic activity	484	909	280	0.0007944	71/+
GO:0050679	positive regulation of epithelial cell proliferation	23	115	6	0.0008086	19/+
GO:0021537	telencephalon development	33	157	7	0.0008123	15/+
GO:0033700	phospholipid efflux	4	10	3	0.0008157	0
GO:0045090	retroviral genome replication	4	10	4	0.0008157	2
GO:0031124	mRNA 3'-end processing	16	81	7	0.0008486	7
GO:0006733	oxidoreduction coenzyme metabolic process	12	59	6	0.0008508	1
GO:0050795	regulation of behavior	28	137	9	0.0008546	6
GO:0016045	detection of bacterium	5	16	3	0.000903	0
GO:0060600	dichotomous subdivision of an epithelial terminal unit	5	16	4	0.000903	0
GO:0072330	monocarboxylic acid biosynthetic process	41	187	6	0.0009038	6
GO:0030890	positive regulation of B cell proliferation	8	35	5	0.0009195	7/+

Table S1 GO biological process GPEA analysis of the RNAseq UC GRN. Shown are the GO identifiers (GOID), the corresponding GO term (Term), the number of interactions in the corresponding GRN subnetwork (edges), the number of genes, the size of the giant connected component of the corresponding subnetwork (gcc), bonferroni adjusted p-value and the number of cancer census genes in the corresponding subnetwork (census). A "+" sign in the census column indicates a significantly over-represented number of cancer census genes in the corresponding subnetwork.

GPEA analysis of the Bead UC GRN for GO biological process

GOID	Term	edges	genes	gcc	padj	census
GO:0022403	cell cycle phase	938	809	312	1.868e-227	66/+
GO:0000278	mitotic cell cycle	797	741	276	4.646e-196	61/+
GO:0006414	translational elongation	142	104	65	2.538e-155	3
GO:0000279	M phase	461	506	146	3.817e-145	35/+
GO:0006415	translational termination	123	88	62	2.053e-144	3
GO:0006955	immune response	991	985	455	3.123e-142	71/+
GO:0006413	translational initiation	160	145	82	2.666e-138	6
GO:0006614	SRP-dependent cotranslational protein targeting to membrane	125	102	63	8.988e-132	4
GO:0006613	cotranslational protein targeting to membrane	125	104	63	1.065e-129	4
GO:0045047	protein targeting to ER	125	104	63	1.065e-129	4
GO:0072599	establishment of protein localization to endoplasmic reticulum	125	105	63	1.116e-128	4
GO:0000087	M phase of mitotic cell cycle	308	357	101	2.473e-128	23/+
GO:0000280	nuclear division	297	347	97	6.642e-126	23/+
GO:0007067	mitosis	297	347	97	6.642e-126	23/+
GO:0016071	mRNA metabolic process	489	572	295	5.478e-124	29
GO:0048285	organelle fission	309	371	100	1.642e-120	23/+
GO:0070972	protein localization to endoplasmic reticulum	126	118	63	1.111e-117	4
GO:0000184	nuclear-transcribed mRNA catabolic process, nonsense-mediated decay	123	115	62	2.944e-116	4
GO:0006412	translation	361	445	200	9.243e-115	17
GO:0006612	protein targeting to membrane	130	150	68	2.981e-97	6
GO:0019080	viral genome expression	129	149	64	8.676e-97	12/+
GO:0019083	viral transcription	129	149	64	8.676e-97	12/+
GO:0006259	DNA metabolic process	674	818	244	1.787e-93	81/+
GO:0000956	nuclear-transcribed mRNA catabolic process	135	165	63	1.104e-92	10
GO:0043624	cellular protein complex disassembly	125	153	62	1.992e-89	4
GO:0051301	cell division	325	453	117	7.663e-88	37/+
GO:0043241	protein complex disassembly	125	158	62	3.583e-86	4
GO:0006402	mRNA catabolic process	136	177	63	3.905e-86	10
GO:0006396	RNA processing	445	606	292	5.704e-84	24
GO:0072594	establishment of protein localization to organelle	147	202	74	1.853e-82	6
GO:0032984	macromolecular complex disassembly	128	179	62	9.605e-77	12
GO:0006401	RNA catabolic process	141	204	64	1.165e-75	10
GO:0019058	viral infectious cycle	149	218	68	1.278e-75	17/+
GO:0060337	type I interferon-mediated signaling pathway	58	68	32	3.223e-61	5
GO:0071357	cellular response to type I interferon	58	68	32	3.223e-61	5
GO:0034340	response to type I interferon	58	69	32	1.719e-60	5
GO:0002682	regulation of immune system process	576	821	306	5.968e-56	85/+
GO:0044764	multi-organism cellular process	435	669	191	1.563e-55	52/+
GO:0016032	viral reproduction	433	667	191	2.015e-55	52/+
GO:0051276	chromosome organization	424	657	201	3.065e-55	75/+
GO:0022904	respiratory electron transport chain	66	96	47	2.8e-54	5
GO:0022415	viral reproductive process	312	527	106	1.742e-51	49/+
GO:0010564	regulation of cell cycle process	235	418	106	8.688e-51	45/+
GO:0045321	leukocyte activation	313	531	139	1.316e-50	65/+
GO:0045087	innate immune response	285	496	144	2.37e-49	28/+
GO:0046649	lymphocyte activation	250	447	90	1.111e-48	62/+
GO:0006260	DNA replication	138	264	70	1.267e-46	25/+
GO:0001775	cell activation	462	729	208	2.464e-46	75/+
GO:0008380	RNA splicing	158	303	113	4.572e-46	15
GO:0006397	mRNA processing	199	375	136	7.469e-46	19
GO:0045333	cellular respiration	80	146	57	2.838e-45	8
GO:0030216	keratinocyte differentiation	60	99	31	2.866e-45	3
GO:0002684	positive regulation of immune system process	284	510	136	1.999e-44	46/+
GO:0008544	epidermis development	136	267	63	3.686e-44	23/+
GO:0007155	cell adhesion	626	910	348	4.749e-44	46/+
GO:0022610	biological adhesion	626	912	348	1.758e-43	46/+
GO:0009913	epidermal cell differentiation	66	118	32	4.007e-43	7
GO:0050776	regulation of immune response	280	510	153	1.131e-42	47/+
GO:0022900	electron transport chain	71	133	50	7.834e-42	5
GO:0051726	regulation of cell cycle	425	704	182	4.329e-41	81/+
GO:0022411	cellular component disassembly	142	288	62	5.937e-41	16
GO:0006974	response to DNA damage stimulus	328	584	186	1.73e-40	63/+
GO:0006954	inflammatory response	252	477	118	3.378e-40	22
GO:0044265	cellular macromolecule catabolic process	393	669	151	4.751e-40	39/+
GO:0051325	interphase	193	386	101	3.829e-39	37/+
GO:0006281	DNA repair	183	374	83	1.448e-37	42/+
GO:0042110	T cell activation	154	323	59	4.262e-37	49/+
GO:0002252	immune effector process	213	427	121	6.734e-37	31/+
GO:0051329	interphase of mitotic cell cycle	185	381	98	1.57e-36	37/+
GO:0000236	mitotic prometaphase	46	83	28	7.024e-36	5
GO:0031424	keratinization	31	43	19	1.135e-35	0
GO:0019884	antigen processing and presentation of exogenous antigen	74	160	24	2.786e-34	3
GO:0043062	extracellular structure organization	90	199	52	4.006e-34	11
GO:0002478	antigen processing and presentation of exogenous peptide antigen	73	158	24	4.137e-34	3
GO:0006334	nucleosome assembly	49	96	12	9.115e-34	5
GO:0030198	extracellular matrix organization	89	198	52	1.096e-33	11
GO:0048002	antigen processing and presentation of peptide antigen	78	172	25	1.492e-33	3
GO:0019221	cytokine-mediated signaling pathway	148	325	50	3.91e-33	29/+
GO:0019882	antigen processing and presentation	89	201	25	1.008e-32	3
GO:0006323	DNA packaging	64	139	12	1.054e-32	9
GO:0065004	protein-DNA complex assembly	58	124	13	2.222e-32	6
GO:0034341	response to interferon-gamma	52	110	29	1.685e-31	9
GO:0007017	microtubule-based process	185	400	62	3.089e-31	27/+
GO:0051249	regulation of lymphocyte activation	121	278	54	5.092e-31	36/+
GO:0031497	chromatin assembly	50	107	12	1.576e-30	9
GO:0044703	multi-organism reproductive process	395	708	117	1.645e-30	55/+
GO:0051607	defense response to virus	77	179	29	1.834e-30	8
GO:0071103	DNA conformation change	74	173	16	5.732e-30	15/+
GO:0000375	RNA splicing, via transesterification reactions	85	203	56	3.62e-29	11
GO:0006091	generation of precursor metabolites and energy	194	425	114	7.893e-29	19
GO:0071345	cellular response to cytokine stimulus	179	399	72	9.876e-29	30/+
GO:0009615	response to virus	107	256	35	1.137e-28	13
GO:0051707	response to other organism	268	545	70	1.981e-28	25
GO:0051320	S phase	60	141	26	2.184e-28	8
GO:0002696	positive regulation of leukocyte activation	89	216	29	3.357e-28	27/+

GO:0009057	macromolecule catabolic process	505	846	236	3.408e-28	50/+
GO:0009607	response to biotic stimulus	285	571	71	3.53e-28	27
GO:0000377	RNA splicing, via transesterification reactions with bulged adenosine as nucleophile	81	198	53	9.554e-28	11
GO:0000398	mRNA splicing, via spliceosome	81	198	53	9.554e-28	11
GO:0071824	protein-DNA complex subunit organization	60	144	13	1.989e-27	12/+
GO:0002694	regulation of leukocyte activation	134	319	57	3.115e-27	37/+
GO:0034097	response to cytokine stimulus	224	482	100	3.7e-27	35/+
GO:0050867	positive regulation of cell activation	91	225	29	5.083e-27	28/+
GO:0060333	interferon-gamma-mediated signaling pathway	35	73	13	1.483e-26	9/+
GO:0006605	protein targeting	215	470	93	1.655e-26	40/+
GO:0006261	DNA-dependent DNA replication	43	98	17	2.179e-26	6
GO:0034728	nucleosome organization	49	116	12	2.359e-26	11/+
GO:0030855	epithelial cell differentiation	120	294	47	3.902e-26	29/+
GO:0050865	regulation of cell activation	143	342	58	5.225e-26	40/+
GO:0000075	cell cycle checkpoint	94	237	47	1.038e-25	28/+
GO:0000226	microtubule cytoskeleton organization	107	268	49	1.795e-25	22/+
GO:0071346	cellular response to interferon-gamma	40	92	16	2.877e-25	9/+
GO:0002479	antigen processing and presentation of exogenous peptide antigen via MHC class I, TAP-dependent	33	70	12	2.937e-25	0
GO:0007059	chromosome segregation	56	140	18	3.428e-25	12/+
GO:0051251	positive regulation of lymphocyte activation	77	198	25	6.676e-25	27/+
GO:0071156	regulation of cell cycle arrest	102	260	48	1.324e-24	30/+
GO:0033365	protein localization to organelle	226	500	84	9.264e-24	47/+
GO:0042590	antigen processing and presentation of exogenous peptide antigen via MHC class I	33	74	12	9.683e-24	0
GO:0006333	chromatin assembly or disassembly	50	128	12	1.618e-23	15/+
GO:0015980	energy derivation by oxidation of organic compounds	121	307	78	1.852e-23	15
GO:0006936	muscle contraction	93	245	43	4.76e-23	6
GO:0050863	regulation of T cell activation	80	213	28	5.578e-23	29/+
GO:0016482	cytoplasmic transport	355	691	160	7.182e-23	54/+
GO:0050870	positive regulation of T cell activation	59	157	23	9.089e-23	23/+
GO:0000084	S phase of mitotic cell cycle	51	134	24	1.223e-22	8
GO:0006886	intracellular protein transport	351	687	168	1.31e-22	52/+
GO:0002474	antigen processing and presentation of peptide antigen via MHC class I	37	91	13	2.24e-22	0
GO:0003012	muscle system process	102	275	43	5.474e-21	8
GO:0034655	nucleobase-containing compound catabolic process	381	734	99	6.339e-21	46/+
GO:0007050	cell cycle arrest	140	359	56	1.133e-20	42/+
GO:0001944	vasculature development	223	510	116	1.401e-20	39/+
GO:0019886	antigen processing and presentation of exogenous peptide antigen via MHC class II	34	87	12	1.852e-20	3
GO:0034660	ncRNA metabolic process	101	276	34	3.141e-20	7
GO:0001501	skeletal system development	138	357	43	3.181e-20	31/+
GO:0050900	leukocyte migration	88	247	38	1.135e-19	18/+
GO:0002495	antigen processing and presentation of peptide antigen via MHC class II	34	90	12	1.55e-19	3
GO:0022616	DNA strand elongation	19	37	8	1.875e-19	2
GO:0002504	antigen processing and presentation of peptide or polysaccharide antigen via MHC class II	34	91	12	3.087e-19	3
GO:0016477	cell migration	439	814	215	3.589e-19	45/+
GO:0048610	cellular process involved in reproduction	226	522	68	3.777e-19	50/+
GO:0044270	cellular nitrogen compound catabolic process	406	776	105	7.843e-19	46/+
GO:0046700	heterocycle catabolic process	406	776	100	7.843e-19	46/+
GO:0016192	vesicle-mediated transport	463	844	216	8.401e-19	34
GO:0070727	cellular macromolecule localization	548	939	245	1.295e-18	66/+
GO:0060429	epithelium development	265	587	86	2.08e-18	57/+
GO:0034613	cellular protein localization	543	935	243	2.268e-18	66/+
GO:0001568	blood vessel development	202	486	105	2.506e-18	38/+
GO:0071294	cellular response to zinc ion	10	10	7	2.577e-18	0
GO:0055114	oxidation-reduction process	225	525	113	3.031e-18	17
GO:0072358	cardiovascular system development	376	742	170	3.67e-18	59/+
GO:0072359	circulatory system development	376	742	170	3.67e-18	59/+
GO:0007051	spindle organization	29	77	22	4.015e-18	6
GO:0071822	protein complex subunit organization	591	988	116	7.479e-18	62/+
GO:0019439	aromatic compound catabolic process	403	778	100	8.955e-18	46/+
GO:0034622	cellular macromolecular complex assembly	219	518	35	1.078e-17	27
GO:0048706	embryonic skeletal system development	37	107	9	1.179e-17	14/+
GO:0006271	DNA strand elongation involved in DNA replication	17	34	7	1.35e-17	0
GO:0022613	ribonucleoprotein complex biogenesis	75	223	12	1.593e-17	9
GO:0045786	negative regulation of cell cycle	168	430	59	2.439e-17	50/+
GO:1901361	organic cyclic compound catabolic process	427	811	115	4.326e-17	46/+
GO:0030595	leukocyte chemotaxis	39	117	23	7.549e-17	6
GO:0031295	T cell costimulation	24	65	7	4.442e-16	10/+
GO:0050778	positive regulation of immune response	119	337	15	4.468e-16	30/+
GO:0032101	regulation of response to external stimulus	134	370	59	6.18e-16	23/+
GO:0007010	cytoskeleton organization	384	765	89	7.405e-16	52/+
GO:0008284	positive regulation of cell proliferation	281	625	76	8.197e-16	60/+
GO:0031294	lymphocyte costimulation	24	66	7	8.882e-16	10/+
GO:0007346	regulation of mitotic cell cycle	101	302	48	7.177e-15	31/+
GO:0048870	cell motility	476	882	224	9.939e-15	51/+
GO:0051674	localization of cell	476	882	224	9.939e-15	51/+
GO:0048704	embryonic skeletal system morphogenesis	26	79	8	2.446e-14	10/+
GO:0002764	immune response-regulating signaling pathway	73	234	11	3.734e-14	28/+
GO:0000082	G1/S transition of mitotic cell cycle	58	191	13	4.615e-14	15/+
GO:0006310	DNA recombination	60	198	36	7.127e-14	24/+
GO:0060326	cell chemotaxis	43	144	24	8.838e-14	8
GO:0002757	immune response-activating signal transduction	68	222	11	9.213e-14	27/+
GO:0006935	chemotaxis	238	573	95	1.989e-13	45/+
GO:0042330	taxis	238	573	95	1.989e-13	45/+
GO:0006325	chromatin organization	194	500	26	2.788e-13	62/+
GO:0030334	regulation of cell migration	130	380	53	1.214e-12	29/+
GO:0030199	collagen fibril organization	14	35	10	1.496e-12	2
GO:0040012	regulation of locomotion	156	436	60	3.476e-12	35/+
GO:0002253	activation of immune response	83	272	13	3.855e-12	27/+
GO:0051701	interaction with host	135	395	16	6.464e-12	37/+
GO:0001525	angiogenesis	116	354	62	7.529e-12	23/+
GO:0048514	blood vessel morphogenesis	150	427	77	1.017e-11	30/+
GO:0007156	homophilic cell adhesion	37	133	12	1.163e-11	3
GO:0002521	leukocyte differentiation	99	317	47	2.218e-11	60/+
GO:2000145	regulation of cell motility	137	403	56	2.635e-11	35/+
GO:0050851	antigen receptor-mediated signaling pathway	31	112	7	2.765e-11	15/+
GO:0006312	mitotic recombination	13	35	7	4.516e-11	2
GO:0019048	virus-host interaction	114	356	16	8.517e-11	37/+

GO:0000216	M/G1 transition of mitotic cell cycle	22	77	7	9.032e-11	0
GO:0065003	macromolecular complex assembly	527	965	84	1.135e-10	61/+
GO:0002429	immune response-activating cell surface receptor signaling pathway	32	120	7	1.776e-10	15/+
GO:0000904	cell morphogenesis involved in differentiation	297	682	113	2.083e-10	59/+
GO:0031145	anaphase-promoting complex-dependent proteasomal ubiquitin-dependent protein catabolic process	22	79	8	2.509e-10	2
GO:0002520	immune system development	210	547	67	2.83e-10	94/+
GO:0002768	immune response-regulating cell surface receptor signaling pathway	34	130	7	4.615e-10	16/+
GO:0002237	response to molecule of bacterial origin	56	207	29	5.277e-10	10
GO:0016337	cell-cell adhesion	126	388	14	5.398e-10	18
GO:0050657	nucleic acid transport	29	112	18	1.066e-09	9
GO:0050658	RNA transport	29	112	18	1.066e-09	9
GO:0051236	establishment of RNA localization	29	112	18	1.066e-09	9
GO:0034470	ncRNA processing	51	193	13	1.2e-09	4
GO:0042254	ribosome biogenesis	37	144	4	1.331e-09	4
GO:0032103	positive regulation of response to external stimulus	38	148	13	1.475e-09	10
GO:0010043	response to zinc ion	11	31	7	2.32e-09	1
GO:0032989	cellular component morphogenesis	522	969	205	2.343e-09	72/+
GO:0031347	regulation of defense response	134	410	40	2.738e-09	29/+
GO:0048705	skeletal system morphogenesis	43	168	8	2.991e-09	15/+
GO:0051270	regulation of cellular component movement	154	452	57	3.762e-09	37/+
GO:0002480	antigen processing and presentation of exogenous peptide antigen via MHC class I, TAP-independent	6	9	6	3.893e-09	0
GO:0035456	response to interferon-beta	7	13	7	4.095e-09	0
GO:0031589	cell-substrate adhesion	55	210	11	5.282e-09	15/+
GO:0030097	hemopoiesis	173	490	58	5.784e-09	86/+
GO:0030098	lymphocyte differentiation	58	220	29	6.304e-09	43/+
GO:0010466	negative regulation of peptidase activity	31	125	17	8.045e-09	6
GO:0006270	DNA replication initiation	10	28	4	9.237e-09	1
GO:0009952	anterior/posterior pattern specification	52	202	9	9.927e-09	24/+
GO:0000902	cell morphogenesis	467	912	162	9.967e-09	68/+
GO:0015931	nucleobase-containing compound transport	33	134	21	1.181e-08	9
GO:0030182	neuron differentiation	505	956	216	1.349e-08	77/+
GO:0043588	skin development	14	49	7	1.456e-08	7/+
GO:0010951	negative regulation of endopeptidase activity	30	123	17	1.86e-08	6
GO:0032201	telomere maintenance via semi-conservative replication	9	24	6	1.924e-08	0
GO:0051983	regulation of chromosome segregation	9	24	5	1.924e-08	2
GO:0006403	RNA localization	29	119	18	1.98e-08	9
GO:0048534	hematopoietic or lymphoid organ development	187	520	62	2.182e-08	90/+
GO:0044403	symbiosis, encompassing mutualism through parasitism	139	427	16	2.241e-08	40/+
GO:0044419	interspecies interaction between organisms	139	427	16	2.241e-08	40/+
GO:0002685	regulation of leukocyte migration	20	80	11	3.07e-08	2
GO:0031175	neuron projection development	280	674	116	4.345e-08	58/+
GO:0032270	positive regulation of cellular protein metabolic process	362	789	73	5.64e-08	63/+
GO:0001655	urogenital system development	73	272	13	7.673e-08	34/+
GO:0051247	positive regulation of protein metabolic process	418	861	81	8.239e-08	72/+
GO:0000722	telomere maintenance via recombination	9	26	6	8.248e-08	0
GO:0009617	response to bacterium	88	313	32	8.424e-08	13
GO:0090068	positive regulation of cell cycle process	38	159	13	9.599e-08	15/+
GO:0032943	mononuclear cell proliferation	43	178	26	1.161e-07	25/+
GO:0032496	response to lipopolysaccharide	48	196	27	1.345e-07	8
GO:0070661	leukocyte proliferation	45	186	27	1.566e-07	27/+
GO:0030335	positive regulation of cell migration	54	218	12	2.555e-07	19/+
GO:0042098	T cell proliferation	29	126	16	2.891e-07	16/+
GO:0006521	regulation of cellular amino acid metabolic process	14	55	8	3.084e-07	2
GO:0030217	T cell differentiation	36	155	17	3.521e-07	32/+
GO:0002687	positive regulation of leukocyte migration	15	61	7	4.325e-07	1
GO:0006119	oxidative phosphorylation	15	61	5	4.325e-07	3
GO:0051603	proteolysis involved in cellular protein catabolic process	118	391	32	4.364e-07	18
GO:0001817	regulation of cytokine production	111	375	36	4.491e-07	29/+
GO:0044257	cellular protein catabolic process	124	405	32	5.162e-07	19
GO:0002443	leukocyte mediated immunity	44	186	9	5.19e-07	16/+
GO:0019941	modification-dependent protein catabolic process	109	371	31	5.516e-07	18
GO:0050852	T cell receptor signaling pathway	20	87	7	5.843e-07	11/+
GO:0045071	negative regulation of viral genome replication	9	29	5	5.898e-07	1
GO:0043632	modification-dependent macromolecule catabolic process	110	374	31	6.694e-07	18
GO:0046651	lymphocyte proliferation	41	176	25	7.025e-07	25/+
GO:0002688	regulation of leukocyte chemotaxis	14	57	10	7.827e-07	1
GO:0007093	mitotic cell cycle checkpoint	30	133	13	7.828e-07	14/+
GO:0031401	positive regulation of protein modification process	298	711	41	9.455e-07	60/+
GO:0033043	regulation of organelle organization	161	484	24	1e-06	46/+
GO:0048525	negative regulation of viral reproduction	9	30	5	1.081e-06	1
GO:2000147	positive regulation of cell motility	54	223	12	1.269e-06	21/+
GO:0006959	humoral immune response	22	99	6	1.463e-06	10/+
GO:0051436	negative regulation of ubiquitin-protein ligase activity involved in mitotic cell cycle	15	64	8	1.617e-06	1
GO:0080134	regulation of response to stress	295	709	107	1.802e-06	58/+
GO:0061061	muscle structure development	141	446	57	1.916e-06	41/+
GO:0006007	glucose catabolic process	17	75	11	1.978e-06	1
GO:0045619	regulation of lymphocyte differentiation	22	100	14	2.119e-06	19/+
GO:0033993	response to lipid	181	526	59	3.149e-06	39/+
GO:0007243	intracellular protein kinase cascade	396	847	108	3.276e-06	81/+
GO:0001816	cytokine production	126	416	38	3.45e-06	33/+
GO:0031399	regulation of protein modification process	518	991	71	4.55e-06	86/+
GO:0033238	regulation of cellular amine metabolic process	16	72	10	4.843e-06	3
GO:0009887	organ morphogenesis	338	773	74	4.951e-06	81/+
GO:0042113	B cell activation	36	163	11	5.055e-06	32/+
GO:0009967	positive regulation of signal transduction	346	784	55	5.243e-06	70/+
GO:0040017	positive regulation of locomotion	55	231	12	5.399e-06	21/+
GO:0031349	positive regulation of defense response	51	218	18	5.847e-06	17/+
GO:0034508	centromere complex assembly	9	33	9	5.871e-06	3
GO:0050920	regulation of chemotaxis	21	98	12	5.898e-06	6
GO:0006511	ubiquitin-dependent protein catabolic process	103	365	29	6.151e-06	18
GO:0010833	telomere maintenance via telomere lengthening	10	39	6	6.315e-06	3
GO:0006096	glycolysis	14	62	9	6.82e-06	1
GO:0006977	DNA damage response, signal transduction by p53 class mediator resulting in cell cycle arrest	14	62	7	6.82e-06	5
GO:0072413	signal transduction involved in mitotic cell cycle checkpoint	14	62	7	6.82e-06	5
GO:0072431	signal transduction involved in mitotic cell cycle G1/S transition DNA damage checkpoint	14	62	7	6.82e-06	5

GO:0072474	signal transduction involved in mitotic cell cycle G1/S checkpoint	14	62	7	6.82e-06	5
GO:0051272	positive regulation of cellular component movement	54	229	12	7.888e-06	23/+
GO:0051352	negative regulation of ligase activity	15	68	8	8.37e-06	2
GO:0051437	positive regulation of ubiquitin-protein ligase activity involved in mitotic cell cycle	15	68	7	8.37e-06	1
GO:0051444	negative regulation of ubiquitin-protein ligase activity	15	68	8	8.37e-06	2
GO:0023056	positive regulation of signaling	377	827	58	9.662e-06	71/+
GO:0072401	signal transduction involved in DNA integrity checkpoint	14	63	7	1.025e-05	6
GO:0072404	signal transduction involved in G1/S transition checkpoint	14	63	7	1.025e-05	5
GO:0072422	signal transduction involved in DNA damage checkpoint	14	63	7	1.025e-05	6
GO:0050795	regulation of behavior	28	132	15	1.028e-05	6
GO:0051439	regulation of ubiquitin-protein ligase activity involved in mitotic cell cycle	16	74	8	1.054e-05	2
GO:0002250	adaptive immune response	40	181	15	1.133e-05	20/+
GO:0051351	positive regulation of ligase activity	17	80	7	1.358e-05	2
GO:0072395	signal transduction involved in cell cycle checkpoint	14	64	7	1.528e-05	6
GO:0006805	xenobiotic metabolic process	30	142	4	1.581e-05	1
GO:0010647	positive regulation of cell communication	377	829	58	1.618e-05	72/+
GO:2000045	regulation of G1/S transition of mitotic cell cycle	20	96	9	1.667e-05	9/+
GO:0007088	regulation of mitosis	19	91	15	1.699e-05	11/+
GO:0051783	regulation of nuclear division	19	91	15	1.699e-05	11/+
GO:0010565	regulation of cellular ketone metabolic process	35	163	11	1.749e-05	9
GO:0042060	wound healing	212	588	52	2.066e-05	37/+
GO:0003002	regionalization	77	302	9	2.147e-05	33/+
GO:0071466	cellular response to xenobiotic stimulus	30	143	4	2.165e-05	1
GO:0051443	positive regulation of ubiquitin-protein ligase activity	16	76	7	2.231e-05	2
GO:0051028	mRNA transport	20	97	14	2.354e-05	8
GO:0030029	actin filament-based process	139	451	21	2.361e-05	30/+
GO:0006909	phagocytosis	19	92	14	2.411e-05	5
GO:0019752	carboxylic acid metabolic process	375	828	69	2.414e-05	22
GO:0072001	renal system development	54	233	12	2.519e-05	28/+
GO:0006749	glutathione metabolic process	10	42	4	2.62e-05	1
GO:0030155	regulation of cell adhesion	62	259	10	2.861e-05	28/+
GO:0000723	telomere maintenance	13	60	9	2.96e-05	8/+
GO:0031055	chromatin remodeling at centromere	7	24	8	3.027e-05	2
GO:0051340	regulation of ligase activity	19	93	8	3.403e-05	4
GO:0031398	positive regulation of protein ubiquitination	26	127	12	3.637e-05	7
GO:0009410	response to xenobiotic stimulus	30	145	4	4.017e-05	1
GO:0016126	sterol biosynthetic process	11	49	6	4.021e-05	1
GO:0006369	termination of RNA polymerase II transcription	10	43	5	4.102e-05	4
GO:0032200	telomere organization	13	61	9	4.374e-05	8/+
GO:0007409	axonogenesis	163	502	22	4.575e-05	49/+
GO:0051438	regulation of ubiquitin-protein ligase activity	18	89	8	5.111e-05	4
GO:0071276	cellular response to cadmium ion	5	13	5	5.139e-05	0
GO:0051336	regulation of hydrolase activity	295	722	56	5.381e-05	51/+
GO:0030030	cell projection organization	431	901	155	5.546e-05	63/+
GO:0002697	regulation of immune effector process	45	205	13	5.843e-05	19/+
GO:0030163	protein catabolic process	165	507	38	6.094e-05	28/+
GO:0048666	neuron development	333	776	134	6.1e-05	63/+
GO:0006695	cholesterol biosynthetic process	10	44	6	6.347e-05	1
GO:0051988	regulation of attachment of spindle microtubules to kinetochore	4	8	4	6.454e-05	1
GO:0010627	regulation of intracellular protein kinase cascade	261	672	60	6.625e-05	68/+
GO:0070647	protein modification by small protein conjugation or removal	224	613	45	6.969e-05	38/+
GO:0002526	acute inflammatory response	21	106	11	8.826e-05	7
GO:0061448	connective tissue development	40	188	11	8.844e-05	19/+
GO:0031571	mitotic cell cycle G1/S transition DNA damage checkpoint	14	69	7	0.0001006	6
GO:0043436	oxoacid metabolic process	453	930	132	0.0001114	28
GO:0031575	mitotic cell cycle G1/S transition checkpoint	15	75	7	0.0001126	8/+
GO:0071158	positive regulation of cell cycle arrest	15	75	7	0.0001126	8/+
GO:0008202	steroid metabolic process	63	268	14	0.0001423	6
GO:0048812	neuron projection morphogenesis	188	554	25	0.0001635	51/+
GO:0051346	negative regulation of hydrolase activity	51	230	17	0.0001764	10
GO:0042102	positive regulation of T cell proliferation	13	65	8	0.0001933	8/+
GO:0045088	regulation of innate immune response	46	213	8	0.0001977	20/+
GO:0048562	embryonic organ morphogenesis	46	213	8	0.0001977	21/+
GO:0006986	response to unfolded protein	25	128	5	0.0002022	8
GO:1901700	response to oxygen-containing compound	303	739	112	0.0002037	43/+
GO:0006520	cellular amino acid metabolic process	128	436	16	0.0002115	9
GO:0006082	organic acid metabolic process	465	947	135	0.0002277	28
GO:0035455	response to interferon-alpha	5	15	5	0.0002309	2
GO:0035637	multicellular organismal signaling	299	734	132	0.0002435	25
GO:0019320	hexose catabolic process	17	89	11	0.0002985	1
GO:0035966	response to topologically incorrect protein	26	134	5	0.0003009	8
GO:0043603	cellular amide metabolic process	26	134	5	0.0003009	3
GO:0000070	mitotic sister chromatid segregation	10	48	7	0.0003259	1
GO:0006336	DNA replication-independent nucleosome assembly	6	22	7	0.0003458	2
GO:0034080	CENP-A containing nucleosome assembly at centromere	6	22	7	0.0003458	2
GO:0034724	DNA replication-independent nucleosome organization	6	22	7	0.0003458	2
GO:0016568	chromatin modification	117	414	16	0.0004023	59/+
GO:0014706	striated muscle tissue development	73	302	12	0.0004233	27/+
GO:0048585	negative regulation of response to stimulus	305	745	82	0.0004265	66/+
GO:0006457	protein folding	43	205	9	0.000427	3
GO:0071779	G1/S transition checkpoint	15	79	7	0.0004328	9/+
GO:0001763	morphogenesis of a branching structure	39	190	8	0.0004382	26/+
GO:0055086	nucleobase-containing small molecule metabolic process	368	831	35	0.0004468	36
GO:0042178	xenobiotic catabolic process	4	10	4	0.0004646	0
GO:0007389	pattern specification process	115	410	12	0.0004651	41/+
GO:0046597	negative regulation of viral entry into host cell	3	5	3	0.0004791	0
GO:0051552	flavone metabolic process	3	5	3	0.0004791	0
GO:0060537	muscle tissue development	77	314	12	0.000496	27/+
GO:0000209	protein polyubiquitination	31	158	10	0.0004994	7
GO:0010648	negative regulation of cell communication	236	641	49	0.0005148	59/+
GO:0023057	negative regulation of signaling	234	638	49	0.0005457	59/+
GO:0022603	regulation of anatomical structure morphogenesis	202	584	39	0.0005472	53/+
GO:0070848	response to growth factor stimulus	123	429	13	0.0005673	50/+
GO:0010948	negative regulation of cell cycle process	20	107	11	0.0005705	17/+
GO:0006508	proteolysis	421	899	75	0.0006062	28
GO:0051094	positive regulation of developmental process	255	672	48	0.0006159	73/+
GO:0048858	cell projection morphogenesis	241	650	40	0.000639	52/+
GO:0002690	positive regulation of leukocyte chemotaxis	10	50	6	0.0006957	1
GO:0050729	positive regulation of inflammatory response	13	69	5	0.0007632	4

GO:0006518	peptide metabolic process	15	81	4	0.0008196	1
GO:0048667	cell morphogenesis involved in neuron differentiation	182	550	25	0.0008575	50/+
GO:0052548	regulation of endopeptidase activity	52	240	23	0.0009715	19/+

Table S2 GO biological process GPEA analysis of the Bead UC GRN. Shown are the GO identifiers (GOID), the corresponding GO term (Term), the number of interactions in the corresponding GRN subnetwork (edges), the number of genes, the size of the giant connected component of the corresponding subnetwork (gcc), bonferroni adjusted p-value and the number of cancer census genes in the corresponding subnetwork (census). A "+" sign in the census column indicates a significantly over-represented number of cancer census genes in the corresponding subnetwork.

GPEA analysis of the Oligo UC GRN for GO biological process

GOID	Term	edges	genes	gcc	padj	census
GO:0006415	translational termination	170	76	60	7.427e-223	2
GO:0006414	translational elongation	178	85	61	1.822e-219	2
GO:0006614	SRP-dependent cotranslational protein targeting to membrane	171	88	59	1.378e-202	3
GO:0045047	protein targeting to ER	172	90	59	7.524e-201	3
GO:0006613	cotranslational protein targeting to membrane	171	90	59	3.07e-199	3
GO:0072599	establishment of protein localization to endoplasmic reticulum	172	91	59	3.387e-199	3
GO:0000184	nuclear-transcribed mRNA catabolic process, nonsense-mediated decay	178	100	61	2.634e-194	3
GO:0070972	protein localization to endoplasmic reticulum	173	102	59	9.88e-184	3
GO:0006413	translational initiation	197	127	68	1.478e-183	4
GO:0022403	cell cycle phase	836	700	335	5.351e-165	66/+
GO:0006612	protein targeting to membrane	175	130	59	9.759e-151	5
GO:0000278	mitotic cell cycle	743	654	274	2.408e-150	61/+
GO:0019080	viral genome expression	177	133	60	5.997e-150	11
GO:0019083	viral transcription	177	133	60	5.997e-150	11
GO:0000956	nuclear-transcribed mRNA catabolic process	184	142	63	6.679e-149	8
GO:0043624	cellular protein complex disassembly	173	132	61	5.816e-146	3
GO:0006402	mRNA catabolic process	186	150	64	5.841e-143	8
GO:0016071	mRNA metabolic process	524	493	289	8.881e-141	28
GO:0043241	protein complex disassembly	173	137	61	1.285e-140	3
GO:0022402	cell cycle process	1031	874	422	1.049e-136	93/+
GO:0006955	immune response	1077	913	577	6.829e-131	71/+
GO:0006401	RNA catabolic process	192	171	65	1.214e-129	8
GO:0032984	macromolecular complex disassembly	177	154	61	1.479e-128	10
GO:0072594	establishment of protein localization to organelle	180	172	61	6.254e-116	5
GO:0000087	M phase of mitotic cell cycle	278	301	97	1.83e-107	22/+
GO:0000279	M phase	389	420	139	7.322e-107	34/+
GO:0019058	viral infectious cycle	194	202	62	3.02e-106	16
GO:0000280	nuclear division	263	292	92	4.625e-102	22/+
GO:0007067	mitosis	263	292	92	4.625e-102	22/+
GO:0006412	translation	323	364	142	3.299e-99	16
GO:0048285	organelle fission	265	312	92	4.063e-91	22
GO:0022904	respiratory electron transport chain	97	88	51	1.537e-90	4
GO:0006952	defense response	972	922	510	2.578e-89	52
GO:0022411	cellular component disassembly	208	258	61	2.658e-81	15
GO:0022900	electron transport chain	102	117	53	2.975e-73	4
GO:0045333	cellular respiration	113	135	58	5.894e-73	8
GO:0008380	RNA splicing	196	261	128	1.014e-70	16
GO:0016032	viral reproduction	521	618	167	2.27e-69	51/+
GO:0044764	multi-organism cellular process	523	620	167	2.761e-69	51/+
GO:0006396	RNA processing	399	508	266	3.08e-66	23
GO:0051301	cell division	280	380	95	8.126e-65	37/+
GO:0022415	viral reproductive process	387	500	116	2.56e-64	48/+
GO:0044265	cellular macromolecule catabolic process	456	571	207	1.858e-63	39/+
GO:0006259	DNA metabolic process	583	692	264	3.186e-61	78/+
GO:0034340	response to type I interferon	62	71	25	1.02e-56	5
GO:0060337	type I interferon-mediated signaling pathway	62	71	25	1.02e-56	5
GO:0071357	cellular response to type I interferon	62	71	25	1.02e-56	5
GO:0006397	mRNA processing	208	317	133	5.74e-52	19
GO:0051320	S phase	92	137	31	1.707e-50	9
GO:0045321	leukocyte activation	337	484	168	6.341e-49	65/+
GO:0010564	regulation of cell cycle process	245	376	95	4.858e-48	47/+
GO:0009611	response to wounding	879	960	543	2.069e-47	61/+
GO:0048002	antigen processing and presentation of peptide antigen	105	167	36	2.661e-47	3
GO:0002682	regulation of immune system process	607	751	314	8.773e-47	84/+
GO:0019882	antigen processing and presentation	118	193	41	6.918e-46	3
GO:0007267	cell-cell signaling	847	942	489	1.007e-45	40
GO:0000375	RNA splicing, via transesterification reactions	110	180	52	2.004e-45	12
GO:0001775	cell activation	510	669	267	2.084e-45	75/+
GO:0000377	RNA splicing, via transesterification reactions with bulged adenosine as nucleophile	107	175	52	2.899e-45	12
GO:0000398	mRNA splicing, via spliceosome	107	175	52	2.899e-45	12
GO:0046649	lymphocyte activation	265	409	112	3.673e-45	62/+
GO:0000084	S phase of mitotic cell cycle	83	131	26	6.239e-45	8
GO:0045087	innate immune response	303	456	150	7.604e-45	28
GO:0048610	cellular process involved in reproduction	285	436	68	2.7e-44	48/+
GO:0030198	extracellular matrix organization	107	178	58	6.345e-44	11
GO:0043062	extracellular structure organization	107	179	58	1.746e-43	11
GO:0002474	antigen processing and presentation of peptide antigen via MHC class I	63	94	18	2.231e-43	0
GO:0050776	regulation of immune response	311	475	147	1.902e-41	47/+
GO:0006271	DNA strand elongation involved in DNA replication	32	32	20	2.488e-41	0
GO:0002478	antigen processing and presentation of exogenous peptide antigen	89	151	21	5.281e-41	3
GO:0019884	antigen processing and presentation of exogenous antigen	90	153	21	5.666e-41	3
GO:0022616	DNA strand elongation	33	35	20	1.692e-40	2
GO:0002684	positive regulation of immune system process	311	478	160	2.024e-40	44/+
GO:0031145	anaphase-promoting complex-dependent proteasomal ubiquitin-dependent protein catabolic process	50	73	16	1.139e-39	2
GO:0051329	interphase of mitotic cell cycle	211	353	103	1.195e-39	38/+
GO:0051325	interphase	213	357	105	3.205e-39	39/+
GO:0006260	DNA replication	130	230	70	3.858e-39	25/+
GO:0044703	multi-organism reproductive process	493	675	152	3.568e-38	54/+
GO:0051276	chromosome organization	388	572	212	1.238e-37	74/+
GO:0007155	cell adhesion	616	791	300	2.747e-36	48/+
GO:0009057	macromolecule catabolic process	547	731	255	3.189e-36	50/+
GO:0007017	microtubule-based process	190	333	64	3.572e-36	26/+
GO:0000075	cell cycle checkpoint	118	217	57	3.939e-36	29/+
GO:0042590	antigen processing and presentation of exogenous peptide antigen via MHC class I	48	75	14	4.123e-36	0
GO:0022610	biological adhesion	617	793	301	6.11e-36	48/+
GO:0002479	antigen processing and presentation of exogenous peptide antigen via MHC class I, TAP-dependent	45	71	14	1.441e-34	0
GO:0051607	defense response to virus	83	157	39	1.539e-33	9
GO:0015980	energy derivation by oxidation of organic compounds	151	283	65	9.797e-33	15
GO:0006091	generation of precursor metabolites and energy	220	387	112	1.676e-32	19
GO:0051726	regulation of cell cycle	425	629	212	5.818e-32	85/+
GO:0002252	immune effector process	225	396	115	7.602e-32	32/+
GO:0007051	spindle organization	43	72	26	1.243e-31	6
GO:0042110	T cell activation	159	300	82	2.087e-31	49/+

GO:0000226	microtubule cytoskeleton organization	115	225	46	2.364e-31	21/+
GO:0071156	regulation of cell cycle arrest	122	238	58	3.053e-31	31/+
GO:0051707	response to other organism	282	478	150	1.692e-29	26
GO:0006605	protein targeting	231	414	64	6.481e-29	37/+
GO:0006261	DNA-dependent DNA replication	47	88	27	8.71e-29	7
GO:0033365	protein localization to organelle	247	439	64	6.914e-28	43/+
GO:0007268	synaptic transmission	341	554	199	9.488e-28	17
GO:0000216	M/G1 transition of mitotic cell cycle	41	76	12	1.92e-27	0
GO:0007059	chromosome segregation	55	111	32	2.202e-27	13/+
GO:0035637	multicellular organismal signaling	436	657	251	2.814e-27	27
GO:0009607	response to biotic stimulus	290	496	154	3.288e-27	28
GO:0002480	antigen processing and presentation of exogenous peptide antigen via MHC class I, TAP-independent	13	9	7	6.005e-26	0
GO:0051443	positive regulation of ubiquitin-protein ligase activity	38	72	12	8.838e-26	2
GO:0006954	inflammatory response	233	428	63	9.489e-26	22
GO:0051351	positive regulation of ligase activity	39	75	12	1.254e-25	2
GO:0019226	transmission of nerve impulse	408	636	223	2.808e-25	27
GO:0055114	oxidation-reduction process	265	473	124	5.474e-25	17
GO:0051249	regulation of lymphocyte activation	120	255	64	8.311e-25	36/+
GO:0050778	positive regulation of immune response	155	317	67	2.055e-24	30/+
GO:0016482	cytoplasmic transport	380	610	87	2.38e-24	51/+
GO:0030182	neuron differentiation	602	823	373	2.985e-24	76/+
GO:0050867	positive regulation of cell activation	97	213	45	3.421e-24	27/+
GO:0002696	positive regulation of leukocyte activation	92	203	34	3.794e-24	26/+
GO:0071103	DNA conformation change	61	135	18	5.677e-24	16/+
GO:0022613	ribonucleoprotein complex biogenesis	85	190	41	1.153e-23	9
GO:0048584	positive regulation of response to stimulus	815	996	532	1.611e-23	93/+
GO:0051438	regulation of ubiquitin-protein ligase activity	40	83	12	1.773e-23	4
GO:0007050	cell cycle arrest	159	327	65	1.818e-23	44/+
GO:0048699	generation of neurons	684	895	438	2.149e-23	90/+
GO:0051340	regulation of ligase activity	41	86	12	2.322e-23	4
GO:0006811	ion transport	606	831	331	3.821e-23	25
GO:0009615	response to virus	104	231	47	5.362e-23	14
GO:0051439	regulation of ubiquitin-protein ligase activity involved in mitotic cell cycle	34	68	12	6.928e-23	2
GO:0051251	positive regulation of lymphocyte activation	81	186	29	2.67e-22	26/+
GO:0002694	regulation of leukocyte activation	135	291	70	2.853e-22	37/+
GO:0051437	positive regulation of ubiquitin-protein ligase activity involved in mitotic cell cycle	32	64	12	3.284e-22	1
GO:0022008	neurogenesis	752	954	484	3.653e-22	96/+
GO:0006974	response to DNA damage stimulus	289	515	143	5.71e-22	64/+
GO:0051436	negative regulation of ubiquitin-protein ligase activity involved in mitotic cell cycle	30	59	11	6.628e-22	1
GO:0007600	sensory perception	187	377	92	7.329e-22	10
GO:0019221	cytokine-mediated signaling pathway	146	313	55	1.801e-21	29/+
GO:0000236	mitotic prometaphase	34	72	22	2.653e-21	5
GO:0006281	DNA repair	149	320	54	5.655e-21	43/+
GO:0050865	regulation of cell activation	146	316	72	1.056e-20	40/+
GO:0007346	regulation of mitotic cell cycle	120	270	39	1.104e-20	31/+
GO:0000082	G1/S transition of mitotic cell cycle	74	177	28	2.06e-20	16/+
GO:0008544	epidermis development	91	215	51	3.746e-20	22/+
GO:0051352	negative regulation of ligase activity	30	64	11	6.833e-20	2
GO:0051444	negative regulation of ubiquitin-protein ligase activity	30	64	11	6.833e-20	2
GO:0050870	positive regulation of T cell activation	61	149	10	1.223e-19	22/+
GO:0006323	DNA packaging	43	103	15	2.336e-19	9
GO:0034655	nucleobase-containing compound catabolic process	377	628	108	2.794e-19	45/+
GO:0071345	cellular response to cytokine stimulus	182	380	64	2.974e-19	30/+
GO:0006521	regulation of cellular amino acid metabolic process	26	54	11	4.615e-19	2
GO:0009410	response to xenobiotic stimulus	50	123	21	4.888e-19	1
GO:0006886	intracellular protein transport	350	600	69	7.184e-19	48/+
GO:0071466	cellular response to xenobiotic stimulus	49	121	21	7.687e-19	1
GO:0042254	ribosome biogenesis	49	122	16	1.521e-18	4
GO:0006977	DNA damage response, signal transduction by p53 class mediator resulting in cell cycle arrest	28	62	12	2.339e-18	5
GO:0072413	signal transduction involved in mitotic cell cycle checkpoint	28	62	12	2.339e-18	5
GO:0072431	signal transduction involved in mitotic cell cycle G1/S transition DNA damage checkpoint	28	62	12	2.339e-18	5
GO:0072474	signal transduction involved in mitotic cell cycle G1/S checkpoint	28	62	12	2.339e-18	5
GO:0006805	xenobiotic metabolic process	48	120	21	2.426e-18	1
GO:0007186	G-protein coupled receptor signaling pathway	266	502	121	3.036e-18	10
GO:0034660	ncRNA metabolic process	87	214	45	3.361e-18	6
GO:0050863	regulation of T cell activation	79	197	14	4.608e-18	29/+
GO:0060333	interferon-gamma-mediated signaling pathway	30	69	17	4.684e-18	9/+
GO:0072401	signal transduction involved in DNA integrity checkpoint	28	63	12	5.44e-18	6
GO:0072404	signal transduction involved in G1/S transition checkpoint	28	63	12	5.44e-18	5
GO:0072422	signal transduction involved in DNA damage checkpoint	28	63	12	5.44e-18	6
GO:0002764	immune response-regulating signaling pathway	90	222	17	8.489e-18	28/+
GO:0072395	signal transduction involved in cell cycle checkpoint	28	64	12	1.245e-17	6
GO:0019439	aromatic compound catabolic process	399	663	117	8.453e-17	45/+
GO:0007586	digestion	39	100	10	8.716e-17	0
GO:0031398	positive regulation of protein ubiquitination	43	113	18	2.278e-16	7
GO:0046700	heterocycle catabolic process	394	660	115	2.546e-16	45/+
GO:0031571	mitotic cell cycle G1/S transition DNA damage checkpoint	28	68	12	2.95e-16	6
GO:0043161	proteasomal ubiquitin-dependent protein catabolic process	72	189	40	4.775e-16	10
GO:0060326	cell chemotaxis	53	142	17	5.822e-16	8
GO:0002768	immune response-regulating cell surface receptor signaling pathway	46	123	10	6.027e-16	16/+
GO:0050851	antigen receptor-mediated signaling pathway	40	106	10	6.7e-16	15/+
GO:0002253	activation of immune response	104	259	27	7.51e-16	27/+
GO:0034097	response to cytokine stimulus	224	457	71	8.508e-16	36/+
GO:0001944	vasculature development	245	486	124	9.57e-16	40/+
GO:0045786	negative regulation of cell cycle	179	391	73	1.005e-15	52/+
GO:0044270	cellular nitrogen compound catabolic process	391	660	112	1.148e-15	45/+
GO:0006936	muscle contraction	91	233	18	1.149e-15	6
GO:0043632	modification-dependent macromolecule catabolic process	133	315	51	1.377e-15	19
GO:0051603	proteolysis involved in cellular protein catabolic process	142	331	52	1.471e-15	19
GO:0002757	immune response-activating signal transduction	81	212	16	1.765e-15	27/+
GO:0006511	ubiquitin-dependent protein catabolic process	128	307	51	2.32e-15	19
GO:0034341	response to interferon-gamma	36	96	17	2.383e-15	9
GO:0002429	immune response-activating cell surface receptor signaling pathway	42	114	10	2.652e-15	15/+
GO:0090068	positive regulation of cell cycle process	55	150	12	2.876e-15	14/+
GO:1901361	organic cyclic compound catabolic process	421	694	121	3.683e-15	45/+
GO:0031575	mitotic cell cycle G1/S transition checkpoint	29	75	12	4.688e-15	9/+

GO:0010498	proteasomal protein catabolic process	74	198	40	5.131e-15	10
GO:0019941	modification-dependent protein catabolic process	130	313	51	7.537e-15	19
GO:0071779	G1/S transition checkpoint	30	79	12	7.975e-15	10/+
GO:0031397	negative regulation of protein ubiquitination	32	86	12	1.189e-14	5
GO:0071822	protein complex subunit organization	604	869	246	1.446e-14	62/+
GO:0007093	mitotic cell cycle checkpoint	44	123	12	1.864e-14	15/+
GO:0016477	cell migration	481	758	280	2.403e-14	49/+
GO:0071346	cellular response to interferon-gamma	31	84	17	2.623e-14	9/+
GO:0071158	positive regulation of cell cycle arrest	28	75	12	4.507e-14	8
GO:0030595	leukocyte chemotaxis	41	116	16	5.224e-14	6
GO:0044257	cellular protein catabolic process	144	342	52	5.759e-14	20
GO:0006935	chemotaxis	271	529	45	6.198e-14	46/+
GO:0042330	taxis	271	529	45	6.198e-14	46/+
GO:0030030	cell projection organization	491	770	200	6.873e-14	64/+
GO:0019752	carboxylic acid metabolic process	446	726	217	7.034e-14	22
GO:0006310	DNA recombination	62	174	25	7.743e-14	24/+
GO:0050900	leukocyte migration	92	245	30	1.77e-13	18
GO:0030163	protein catabolic process	198	431	59	1.868e-13	29/+
GO:0003012	muscle system process	100	262	20	2.022e-13	8
GO:2000026	regulation of multicellular organismal development	703	958	452	2.64e-13	115/+
GO:0033238	regulation of cellular amine metabolic process	26	71	11	2.845e-13	3
GO:0034728	nucleosome organization	31	88	8	3.359e-13	10/+
GO:0050852	T cell receptor signaling pathway	30	85	8	4.018e-13	11/+
GO:0006082	organic acid metabolic process	552	831	288	4.425e-13	29
GO:0000904	cell morphogenesis involved in differentiation	328	603	71	4.432e-13	59/+
GO:0006812	cation transport	273	537	166	6.87e-13	17
GO:0001568	blood vessel development	217	462	115	8.197e-13	39/+
GO:0048666	neuron development	392	677	202	1.35e-12	62/+
GO:0006334	nucleosome assembly	25	70	8	1.439e-12	5
GO:0070727	cellular macromolecule localization	527	811	212	1.454e-12	62/+
GO:0034613	cellular protein localization	522	807	211	1.889e-12	62/+
GO:0043436	oxoacid metabolic process	532	817	284	2.585e-12	29
GO:0031497	chromatin assembly	27	78	8	2.798e-12	9/+
GO:0072358	cardiovascular system development	413	701	184	2.91e-12	60/+
GO:0072359	circulatory system development	413	701	184	2.91e-12	60/+
GO:0019886	antigen processing and presentation of exogenous peptide antigen via MHC class II	27	79	7	5.165e-12	3
GO:0006119	oxidative phosphorylation	20	54	14	5.558e-12	2
GO:0031175	neuron projection development	314	593	121	6.964e-12	58/+
GO:0019048	virus-host interaction	141	348	32	9.416e-12	37/+
GO:0051701	interaction with host	161	383	32	1.443e-11	37/+
GO:0045595	regulation of cell differentiation	582	867	372	1.97e-11	120/+
GO:0002495	antigen processing and presentation of peptide antigen via MHC class II	27	82	7	3.062e-11	3
GO:0006508	proteolysis	450	745	88	3.671e-11	28
GO:0071824	protein-DNA complex subunit organization	37	116	8	4.549e-11	11/+
GO:0000902	cell morphogenesis	485	780	191	4.767e-11	68/+
GO:0002504	antigen processing and presentation of peptide or polysaccharide antigen via MHC class II	27	83	7	5.439e-11	3
GO:0016192	vesicle-mediated transport	443	739	202	5.505e-11	33
GO:0042773	ATP synthesis coupled electron transport	16	42	13	5.613e-11	1
GO:0042775	mitochondrial ATP synthesis coupled electron transport	16	42	13	5.613e-11	1
GO:0006333	chromatin assembly or disassembly	31	97	8	6.167e-11	14/+
GO:0001501	skeletal system development	130	333	27	7.051e-11	33/+
GO:0031396	regulation of protein ubiquitination	49	153	19	7.923e-11	14/+
GO:0070647	protein modification by small protein conjugation or removal	231	493	136	9.54e-11	38/+
GO:0032989	cellular component morphogenesis	536	830	237	1.073e-10	72/+
GO:0071294	cellular response to zinc ion	7	9	7	1.21e-10	0
GO:0048858	cell projection morphogenesis	282	561	53	1.276e-10	52/+
GO:0006325	chromatin organization	184	425	35	1.295e-10	60/+
GO:2000045	regulation of G1/S transition of mitotic cell cycle	29	92	12	1.661e-10	10/+
GO:0045664	regulation of neuron differentiation	101	279	18	1.765e-10	26/+
GO:0007088	regulation of mitosis	25	78	16	1.842e-10	10/+
GO:0051783	regulation of nuclear division	25	78	16	1.842e-10	10/+
GO:0006629	lipid metabolic process	644	926	417	1.871e-10	39
GO:0002443	leukocyte mediated immunity	58	180	22	2.373e-10	18/+
GO:0051960	regulation of nervous system development	155	380	19	2.647e-10	40/+
GO:0006364	rRNA processing	27	86	8	2.889e-10	1
GO:0009617	response to bacterium	97	272	25	3.086e-10	13
GO:0048870	cell motility	508	808	288	4.992e-10	55/+
GO:0051674	localization of cell	508	808	288	4.992e-10	55/+
GO:0010628	positive regulation of gene expression	666	947	408	5.24e-10	152/+
GO:0032990	cell part morphogenesis	287	571	57	5.486e-10	52/+
GO:0007389	pattern specification process	130	338	37	5.573e-10	42/+
GO:0065004	protein-DNA complex assembly	30	98	8	6.33e-10	6
GO:0048812	neuron projection morphogenesis	226	491	49	6.809e-10	51/+
GO:0060284	regulation of cell development	183	428	29	7.954e-10	49/+
GO:0048878	chemical homeostasis	458	761	213	8.155e-10	43
GO:0016072	rRNA metabolic process	27	88	8	8.424e-10	1
GO:0048667	cell morphogenesis involved in neuron differentiation	222	486	49	8.708e-10	50/+
GO:0042060	wound healing	267	547	65	9.387e-10	37/+
GO:0000077	DNA damage checkpoint	36	119	12	1.015e-09	16/+
GO:0001816	cytokine production	149	374	81	1.413e-09	31/+
GO:0000819	sister chromatid segregation	15	43	10	1.902e-09	1
GO:0006007	glucose catabolic process	22	71	13	1.92e-09	1
GO:0031570	DNA integrity checkpoint	37	124	12	2.362e-09	16/+
GO:0000209	protein polyubiquitination	42	140	13	2.755e-09	7
GO:0044403	symbiosis, encompassing mutualism through parasitism	171	413	33	3.683e-09	40/+
GO:0044419	interspecies interaction between organisms	171	413	33	3.683e-09	40/+
GO:0050767	regulation of neurogenesis	129	341	19	3.802e-09	40/+
GO:0000070	mitotic sister chromatid segregation	14	40	10	4.201e-09	1
GO:0031581	hemidesmosome assembly	7	12	7	1.097e-08	0
GO:0030001	metal ion transport	176	424	93	1.147e-08	16
GO:0006520	cellular amino acid metabolic process	154	388	28	1.157e-08	9
GO:0031424	keratinization	8	16	5	1.237e-08	0
GO:0030216	keratinocyte differentiation	20	67	13	1.551e-08	3
GO:0006096	glycolysis	18	59	11	1.668e-08	1
GO:0007052	mitotic spindle organization	12	34	10	2.493e-08	2
GO:0030330	DNA damage response, signal transduction by p53 class mediator	29	102	12	2.498e-08	14/+
GO:0031295	T cell costimulation	18	60	6	2.896e-08	8/+
GO:0019320	hexose catabolic process	24	84	13	3.083e-08	1

GO:0032787	monocarboxylic acid metabolic process	127	343	27	3.318e-08	13
GO:0044093	positive regulation of molecular function	664	957	414	3.439e-08	89/+
GO:0016337	cell-cell adhesion	112	315	13	4.326e-08	19
GO:0002250	adaptive immune response	51	173	11	4.509e-08	22/+
GO:0031294	lymphocyte costimulation	18	61	6	4.973e-08	8/+
GO:0007417	central nervous system development	296	596	78	8.632e-08	64/+
GO:0042770	signal transduction in response to DNA damage	31	112	12	9.403e-08	16/+
GO:0006270	DNA replication initiation	10	27	7	9.733e-08	2
GO:0001525	angiogenesis	121	335	35	1.106e-07	25/+
GO:0006702	androgen biosynthetic process	7	14	6	1.113e-07	0
GO:0050878	regulation of body fluid levels	250	538	52	1.213e-07	35/+
GO:0046365	monosaccharide catabolic process	24	87	13	1.291e-07	1
GO:0001819	positive regulation of cytokine production	51	176	20	1.501e-07	14
GO:0048514	blood vessel morphogenesis	161	407	77	1.622e-07	31/+
GO:1901700	response to oxygen-containing compound	379	694	99	1.727e-07	44/+
GO:0007156	homophilic cell adhesion	24	88	6	2.049e-07	3
GO:0001817	regulation of cytokine production	120	335	63	2.193e-07	27/+
GO:0007409	axonogenesis	186	448	42	2.279e-07	49/+
GO:0006457	protein folding	50	175	18	3.107e-07	3
GO:0051094	positive regulation of developmental process	306	612	68	3.135e-07	72/+
GO:0072331	signal transduction by p53 class mediator	33	122	12	3.476e-07	17/+
GO:0048706	embryonic skeletal system development	26	97	9	3.769e-07	16/+
GO:0032446	protein modification by small protein conjugation	178	437	56	3.829e-07	34/+
GO:0030855	epithelial cell differentiation	78	248	21	3.839e-07	29/+
GO:0031347	regulation of defense response	142	377	45	3.871e-07	29/+
GO:0043085	positive regulation of catalytic activity	470	791	127	4.798e-07	70/+
GO:0044057	regulation of system process	159	407	50	5.144e-07	21
GO:0046651	lymphocyte proliferation	45	162	10	6.078e-07	25/+
GO:0007599	hemostasis	189	456	27	7.199e-07	31/+
GO:0046903	secretion	365	683	75	7.446e-07	24
GO:0010833	telomere maintenance via telomere lengthening	11	35	4	9.198e-07	3
GO:0043269	regulation of ion transport	74	241	21	9.52e-07	12
GO:0034470	ncRNA processing	42	154	8	9.668e-07	3
GO:0050801	ion homeostasis	291	597	92	9.669e-07	31
GO:0007010	cytoskeleton organization	347	664	58	1.116e-06	51/+
GO:0032943	mononuclear cell proliferation	45	164	10	1.303e-06	25/+
GO:0007596	blood coagulation	185	452	27	1.485e-06	30/+
GO:0006403	RNA localization	30	115	16	1.488e-06	10
GO:0030199	collagen fibril organization	10	31	8	1.493e-06	2
GO:0009913	epidermal cell differentiation	22	85	14	1.837e-06	8
GO:0000086	G2/M transition of mitotic cell cycle	35	133	13	1.867e-06	15/+
GO:0070661	leukocyte proliferation	47	171	10	1.893e-06	27/+
GO:0042446	hormone biosynthetic process	15	55	8	1.905e-06	1
GO:0044255	cellular lipid metabolic process	348	667	131	2.029e-06	28
GO:0033993	response to lipid	210	491	23	2.338e-06	40/+
GO:0006120	mitochondrial electron transport, NADH to ubiquinone	10	32	7	2.778e-06	0
GO:0060338	regulation of type I interferon-mediated signaling pathway	10	32	6	2.778e-06	4
GO:0050817	coagulation	185	454	27	2.855e-06	30/+
GO:0010565	regulation of cellular ketone metabolic process	40	151	14	3.389e-06	10
GO:0042178	xenobiotic catabolic process	5	9	4	4.504e-06	0
GO:0048598	embryonic morphogenesis	158	412	34	4.716e-06	50/+
GO:0016567	protein ubiquitination	155	407	42	4.804e-06	32/+
GO:0030071	regulation of mitotic metaphase/anaphase transition	10	33	7	5.055e-06	6/+
GO:0008202	steroid metabolic process	77	253	10	5.103e-06	6
GO:0006631	fatty acid metabolic process	72	241	18	5.19e-06	11
GO:0060429	epithelium development	211	495	37	5.275e-06	58/+
GO:0006694	steroid biosynthetic process	33	129	8	5.59e-06	3
GO:0045893	positive regulation of transcription, DNA-dependent	543	868	331	6.029e-06	141/+
GO:0009887	organ morphogenesis	358	682	148	6.476e-06	82/+
GO:0051254	positive regulation of RNA metabolic process	592	912	369	6.477e-06	144/+
GO:0002237	response to molecule of bacterial origin	54	195	8	7.418e-06	10
GO:0032940	secretion by cell	289	601	40	7.617e-06	20
GO:0045071	negative regulation of viral genome replication	9	29	3	9.223e-06	1
GO:0043588	skin development	12	44	5	9.368e-06	7/+
GO:0032201	telomere maintenance via semi-conservative replication	8	24	4	9.525e-06	0
GO:0030593	neutrophil chemotaxis	13	49	6	1.005e-05	3
GO:0018916	nitrobenzene metabolic process	3	3	3	1.013e-05	0
GO:0070458	cellular detoxification of nitrogen compound	3	3	3	1.013e-05	0
GO:0032101	regulation of response to external stimulus	118	343	19	1.196e-05	23
GO:2000602	regulation of interphase of mitotic cell cycle	35	138	12	1.226e-05	18/+
GO:0009790	embryo development	434	766	223	1.505e-05	99/+
GO:0007091	mitotic metaphase/anaphase transition	10	35	7	1.577e-05	7/+
GO:0048525	negative regulation of viral reproduction	9	30	3	1.678e-05	1
GO:0000722	telomere maintenance via recombination	8	25	4	1.828e-05	0
GO:0006468	protein phosphorylation	665	978	480	2.149e-05	100/+
GO:0034220	ion transmembrane transport	75	253	18	2.483e-05	7
GO:0030334	regulation of cell migration	127	363	31	2.96e-05	30/+
GO:0006959	humoral immune response	23	96	9	3.109e-05	10/+
GO:0072376	protein activation cascade	15	61	7	3.112e-05	0
GO:0010043	response to zinc ion	8	26	7	3.409e-05	1
GO:0006695	cholesterol biosynthetic process	11	42	8	4.222e-05	1
GO:0002460	adaptive immune response based on somatic recombination of immune receptors built from immunoglobulin superfamily domains	39	155	8	4.473e-05	18/+
GO:0031399	regulation of protein modification process	558	889	374	5.407e-05	90/+
GO:0042113	B cell activation	37	149	12	5.497e-05	32/+
GO:0055065	metal ion homeostasis	100	312	28	6.199e-05	12
GO:0016126	sterol biosynthetic process	12	48	8	6.512e-05	1
GO:0022617	extracellular matrix disassembly	9	33	6	8.883e-05	1
GO:0042180	cellular ketone metabolic process	51	194	14	9.238e-05	12
GO:0034754	cellular hormone metabolic process	19	82	8	9.529e-05	2
GO:0006956	complement activation	11	44	6	0.0001098	0
GO:0044702	single organism reproductive process	239	545	125	0.0001229	41/+
GO:0016568	chromatin modification	126	366	34	0.0001289	57/+
GO:0045088	regulation of innate immune response	54	204	9	0.0001455	20/+
GO:0002449	lymphocyte mediated immunity	34	142	6	0.0001634	15/+
GO:0006873	cellular ion homeostasis	231	535	57	0.0001665	29
GO:0007243	intracellular protein kinase cascade	422	762	106	0.0001677	81/+
GO:0055085	transmembrane transport	249	560	131	0.0001841	16
GO:0032496	response to lipopolysaccharide	47	185	8	0.000225	8

GO:0003002	regionalization	72	253	17	0.0002417	34/+
GO:0097237	cellular response to toxin	5	13	3	0.0002427	1
GO:0080134	regulation of response to stress	324	656	148	0.0002985	58/+
GO:0032103	positive regulation of response to external stimulus	34	144	7	0.0003144	10
GO:0009308	amine metabolic process	43	174	12	0.000338	3
GO:0055082	cellular chemical homeostasis	267	586	65	0.0003421	30
GO:0042742	defense response to bacterium	20	90	10	0.0003607	2
GO:0048704	embryonic skeletal system morphogenesis	16	72	5	0.0003912	12/+
GO:0044106	cellular amine metabolic process	37	155	12	0.0003951	3
GO:0007610	behavior	179	461	23	0.0003996	23
GO:0043900	regulation of multi-organism process	47	187	9	0.0004182	18/+
GO:0051049	regulation of transport	525	867	241	0.0005225	65/+
GO:0050832	defense response to fungus	5	14	3	0.0005282	0
GO:0023056	positive regulation of signaling	398	741	118	0.0005306	70/+
GO:0045597	positive regulation of cell differentiation	167	443	20	0.0005758	60/+
GO:0034330	cell junction organization	42	173	13	0.0006579	13
GO:0042325	regulation of phosphorylation	414	759	252	0.0007146	83/+
GO:0050657	nucleic acid transport	24	109	14	0.0007432	10
GO:0050658	RNA transport	24	109	14	0.0007432	10
GO:0051236	establishment of RNA localization	24	109	14	0.0007432	10
GO:0032200	telomere organization	13	59	4	0.0007802	9/+
GO:0032963	collagen metabolic process	12	54	10	0.0008441	3
GO:0006458	’de novo’ protein folding	11	49	5	0.0009667	1

Table S3 GO biological process GPEA analysis of the Oligo UC GRN. Shown are the GO identifiers (GOID), the corresponding GO term (Term), the number of interactions in the corresponding GRN subnetwork (edges), the number of genes, the size of the giant connected component of the corresponding subnetwork (gcc), bonferroni adjusted p-value and the number of cancer census genes in the corresponding subnetwork (census). A "+" sign in the census column indicates a significantly over-represented number of cancer census genes in the corresponding subnetwork.

GPEA analysis of the RNAseq UC GRN for gene sets of co-located genes

chr	locus	start	size	edges	pvalue	census
chr5	q31.3	140000001	74	159	3.6673e-222	
chr17	q21.2	39000001	61	136	7.6570e-204	
chr17	q21.2	38500001	49	119	1.1284e-194	RARA, SMARCE1
chr6	p22.2	25500001	50	105	1.0881e-163	
chr6	p22.2/p22.1	26000001	48	98	2.9016e-153	
chr5	q31.3	140500001	54	103	1.6926e-152	
chr19	q13.43	57500001	41	90	3.9091e-150	
chr8	q24.3	145000001	43	81	1.5314e-127	RECQL4
chr19	q13.43	58000001	46	75	8.4630e-111	
chr16	p11.2	30000001	54	80	4.0300e-109	
chr21	q22.11	31500001	38	67	5.2390e-107	
chr1	q21.3	152500001	43	69	2.0553e-103	
chr9	q34.3	139500001	67	83	7.6570e-99	
chr17	q25.3	79500001	47	67	3.9091e-94	ASPSCR1
chr19	q13.31	44000001	37	60	4.0300e-94	
chr16	p11.2	30500001	49	68	1.7329e-93	FUS
chr8	q24.3	145500001	37	58	4.8360e-90	RECQL4
chr11	q13.1/q13.2	65000001	46	64	8.8660e-90	
chr3	p25.3	9500001	34	55	3.1837e-88	FANCD2, VHL
chr5	q31.3	139500001	42	60	3.1434e-87	
chr6	p22.1	27000001	32	51	8.8660e-83	HIST1H4I
chr1	q21.3	152000001	34	52	5.2390e-82	
chr16	p11.2	29500001	45	59	1.1687e-81	
chrX	p11.23	48500001	50	62	1.8941e-81	GATA1, TFE3, WAS
chr6	p21.33/p21.32	31500001	79	78	1.2896e-79	
chr19	p13.3	500001	51	60	7.6570e-77	FSTL3, STK11
chr1	p36.33	1000001	48	58	1.7732e-76	
chr19	p13.3	1000001	43	54	1.1687e-74	STK11, TCF3
chr1	q21.3	150500001	35	48	1.5314e-72	MLLT11, ARNT
chr1	q22	155000001	42	51	3.3852e-70	MUC1
chr8	q24.3	144500001	39	49	9.2690e-70	
chr19	q13.41/q13.42	53000001	31	44	1.6926e-69	
chr19	p13.11/p12	19000001	33	45	5.2390e-69	
chr16	p13.3	1	52	56	5.6420e-69	AXIN1
chrX	p11.23/p11.22	49000001	44	51	4.4330e-68	
chr11	q13.1/q13.2	65500001	46	52	7.6570e-68	
chr6	p22.1	27500001	36	46	2.1359e-67	
chr19	q13.33	49500001	70	64	2.9822e-66	
chr1	q23.3	161000001	36	45	1.8941e-65	FCGR2B, SDHC
chr19	q13.31/q13.32	44500001	36	45	1.8941e-65	CBLC, BCL3
chr3	p21.31	48500001	42	48	1.1284e-64	NCKIPSD
chr19	q13.43	58500001	30	41	1.4911e-64	
chr16	p13.3	1500001	59	57	2.1762e-64	TSC2, TRAF7
chr1	q21.3	151000001	37	45	2.4583e-64	MLLT11
chr11	p15.5	1	50	52	5.2390e-64	HRAS
chr1	q21.3/q22	154500001	42	47	7.6570e-63	MUC1
chr19	q13.12	36000001	48	50	1.9747e-62	
chr11	q13.1	64500001	56	54	4.4330e-62	MEN1
chr19	p13.2	11500001	30	39	1.9747e-60	
chr11	q13.2	67000001	36	42	1.1284e-59	

Table S4 Chromosomal GPEA analysis of the RNAseq UC GRN. Shown are the chromosomal location of the 1 Mb window by the chromosome (chr), chromosomal band (locus) and start nucleotide position (start), the size of the GRN subnetwork corresponding to the genomic region, the number of interactions of the subnetwrok, the bonferroni adjusted p-value and the list of cancer census genes which are co-located in the respective genomic 1 Mb window.

GPEA analysis of the Bead UC GRN for gene sets of co-located genes

chr	locus	start	size	edges	pvalue	census
chr8	q24.3	145000001	43	60	2.08131e-90	RECQL4
chr6	p22.2/p22.1	26000001	48	54	2.23839e-73	
chr6	p22.2	25500001	50	54	2.00277e-71	
chr1	q21.3	152500001	43	46	1.76715e-63	
chr17	q25.3	79500001	47	41	4.31970e-51	ASPSCR1
chr6	p22.1	27000001	32	34	7.06860e-51	HIST1H4I
chr8	q24.3	145500001	37	35	2.51328e-48	RECQL4
chr8	q24.3	144500001	39	30	1.02102e-37	
chr8	p11.23/p11.22	37500001	20	21	3.49503e-35	WHSC1L1
chr1	q23.3	161000001	36	27	2.04204e-34	FCGR2B, SDHC
chr11	q13.2	67000001	36	25	7.06860e-31	
chr17	q21.32	46000001	31	23	1.80642e-30	
chr17	q21.32/q21.33	46500001	31	23	1.80642e-30	
chr1	q21.3	153000001	43	27	3.29868e-30	
chr11	p15.5	1	50	29	1.06029e-29	HRAS
chr16	p13.3	1	52	29	1.02102e-28	AXIN1
chr19	q13.43	57500001	41	25	5.10510e-28	
chr1	q23.3	160500001	34	22	7.85400e-27	SDHC
chr1	p34.3/p34.2	40000001	23	18	2.74890e-26	MYCL1
chr6	p22.1	27500001	36	22	1.02102e-25	
chr1	p34.2	40500001	22	17	5.89050e-25	
chr1	q21.3	150500001	35	20	7.46130e-23	MLLT11, ARNT
chr1	q21.3/q22	154500001	42	22	9.42480e-23	MUC1
chr6	p21.32/p21.31	32500001	42	22	9.42480e-23	DAXX
chr9	q34.3	139500001	67	28	2.98452e-21	
chr19	q13.43	58000001	46	22	4.71240e-21	
chr1	q21.3	151000001	37	19	2.82744e-20	MLLT11
chr16	p13.3	500001	48	22	3.10233e-20	
chr3	p21.31	49500001	38	19	7.85400e-20	
chr1	q22	155000001	42	20	1.09956e-19	MUC1
chr11	q13.2	66500001	35	18	1.49226e-19	
chr5	q31.3	140000001	74	28	5.89050e-19	
chr22	q13.33	50000001	30	16	1.68861e-18	
chr4	q13.2/q13.3	69500001	9	9	2.67036e-17	
chr6	p21.1	42500001	33	16	3.69138e-17	
chr12	q15	69000001	15	11	3.76992e-17	MDM2
chr12	q13.3/q14.1	57500001	37	17	3.92700e-17	CDK4, DDIT3
chr17	q11.2	26500001	49	20	4.71240e-17	
chr19	p13.2	7500001	42	18	1.02102e-16	
chr9	q34.3	140000001	35	16	2.43474e-16	
chr20	q13.12	43500001	39	17	2.43474e-16	SDC4
chr22	q13.33	50500001	32	15	5.89050e-16	
chr5	q31.3	140500001	54	20	2.04204e-15	
chr1	q21.3	152000001	34	15	3.76992e-15	
chr7	p15.2/p15.1	27000001	22	12	3.76992e-15	JAZF1, HOXA11, HOXA13
chr16	q12.2/q13/q21	56500001	34	15	3.76992e-15	HERPUD1
chr2	q35	219500001	47	18	5.49780e-15	FEV
chr17	p13.1	7000001	68	23	5.89050e-15	TP53
chr11	q13.1	64500001	56	20	8.24670e-15	MEN1
chr8	p11.21	41500001	12	9	1.02102e-14	KAT6A

Table S5 Chromosomal GPEA analysis of the Bead UC GRN. Shown are the chromosomal location of the 1 Mb window by the chromosome (chr), chromosomal band (locus) and start nucleotide position (start), the size of the GRN subnetwork corresponding to the genomic region, the number of interactions of the subnetwrok, the bonferroni adjusted p-value and the list of cancer census genes which are co-located in the respective genomic 1 Mb window.

GPEA analysis of the Oligo UC GRN for gene sets of co-located genes

chr	locus	start	size	edges	pvalue	census
chr1	q23.3	161000001	36	27	4.82850e-30	FCGR2B, SDHC
chr17	q25.3	79500001	47	30	5.15040e-28	ASPSCR1
chr1	q21.3	150500001	35	25	1.80264e-27	MLLT11, ARNT
chr11	p15.5	1	50	25	8.36940e-20	HRAS
chr8	q24.3	145000001	43	22	8.04750e-19	RECQL4
chr1	p34.3/p34.2	40000001	23	15	6.11610e-18	MYCL1
chr1	q23.3	160500001	34	18	3.86280e-17	SDHC
chr19	q13.2/q13.31	43000001	20	13	4.18470e-16	
chr9	p21.1/p13.3	32500001	17	12	4.50660e-16	
chr16	p13.2	8000001	7	8	1.41636e-15	
chr6	p21.1	42500001	33	16	1.25541e-14	
chr1	q21.2/q21.3	150000001	27	14	3.54090e-14	ARNT
chr8	p11.21	42000001	17	11	4.18470e-14	
chr16	p13.2	8500001	9	8	2.06016e-13	
chr16	q21/q22.1	66500001	42	17	1.31979e-12	CBFB
chr17	q12/q21.1/q21.2	37500001	31	14	1.67388e-12	ERBB2, CDK12, RARA
chr4	q13.3	74500001	14	9	5.47230e-12	
chr6	p21.1	43000001	26	12	1.80264e-11	
chr1	q21.3/q22	154500001	42	16	2.44644e-11	MUC1
chr4	q13.3	74000001	16	9	7.40370e-11	
chr8	p11.23/p11.22	37500001	20	10	9.65700e-11	FGFR1, WHSC1L1
chr11	q13.2	67000001	36	14	1.03008e-10	
chr9	p21.3	20500001	24	11	1.06227e-10	MLLT3
chr1	p34.2	40500001	22	10	6.75990e-10	
chr11	p11.2	47000001	22	10	6.75990e-10	DDB2
chr13	q34	113500001	18	9	6.75990e-10	
chr2	p22.1	39000001	11	7	1.22322e-09	
chr13	q14.2	48500001	11	7	1.22322e-09	RB1
chr19	p13.12	15000001	27	11	1.41636e-09	BRD4
chr17	q25.3	80000001	23	10	1.67388e-09	
chr17	p13.3	500001	19	9	1.83483e-09	YWHAE
chr1	p34.3	37500001	20	9	4.82850e-09	
chr1	q22	155000001	42	14	6.75990e-09	MUC1
chr9	p21.3	21000001	25	10	9.01320e-09	CDKN2A
chr3	p25.3	9500001	34	12	1.06227e-08	VHL
chr16	q12.2/q13/q21	56500001	34	12	1.06227e-08	HERPUD1
chr19	q13.33/q13.41	51000001	49	15	3.21900e-08	KLK2
chr1	p34.3	38000001	18	8	3.86280e-08	
chr11	q13.1/q13.2	65000001	46	14	7.40370e-08	
chr11	q13.1/q13.2	65500001	46	14	7.40370e-08	
chr19	q13.31	44000001	37	12	7.72560e-08	
chr20	p13	3000001	28	10	8.69130e-08	
chr6	p21.32/p21.31	32500001	42	13	9.97890e-08	DAXX
chr12	q15	69000001	15	7	1.31979e-07	MDM2
chr20	q11.22/q11.23	33500001	24	9	1.35198e-07	
chr1	q21.3	153000001	43	13	1.80264e-07	
chr9	p21.1/p13.3	33000001	20	8	2.12454e-07	
chr22	q11.21	20500001	20	8	2.12454e-07	
chr8	q24.3	144500001	39	12	2.60739e-07	
chr11	q13.2	66500001	35	11	3.86280e-07	

Table S6 Chromosomal GPEA analysis of the Oligo UC GRN. Shown are the chromosomal location of the 1 Mb window by the chromosome (chr), chromosomal band (locus) and start nucleotide position (start), the size of the GRN subnetwork corresponding to the genomic region, the number of interactions of the subnetwrok, the bonferroni adjusted p-value and the list of cancer census genes which are co-located in the respective genomic 1 Mb window.

GPEA analysis of the RNAseq UC GRN for gene families

tag	name	genes	edges	bonf
CD	CD molecules	380	591	0.0e+00
HIST	Histones / Replication-dependent	67	236	0.0e+00
KRTAP	Keratin associated proteins	75	237	0.0e+00
SNORA	ncRNAs / Small nucleolar RNAs : H/ACA box containing	85	254	0.0e+00
ZKRAB	-	338	764	0.0e+00
ZNF	Zinc fingers, C2H2-type	697	1307	0.0e+00
PCDHC	Cadherins / Protocadherins : Clustered	57	150	7.8e-242
RPL	L ribosomal proteins	59	119	1.2e-175
KRT	-	55	87	1.4e-121
HLA	Histocompatibility complex	24	46	1.1e-85
IGD	Immunoglobulin superfamily / Immunoglobulin-like domain containing	233	175	3.6e-85
LNCRNA	Long non-coding RNAs	548	407	1.2e-78
C1SET	Immunoglobulin superfamily / C1-set domain containing	38	51	1.0e-75
HOXL	Homeoboxes / ANTP class : HOXL subclass	52	54	1.6e-66
LCE	Late cornified envelopes	17	30	1.5e-59
RPS	S ribosomal proteins	34	40	7.2e-59
OR2	GPCR / Class A : Olfactory receptors	61	50	6.0e-53
IFF1	Intermediate filaments type I, keratins (acidic)	28	33	2.3e-51
VSET	Immunoglobulin superfamily / V-set domain containing	161	95	1.4e-50
PRAME	-	15	25	2.3e-50
KLK	Kallikreins	17	26	1.2e-49
RBM	RNA binding motif (RRM) containing	209	114	2.6e-46
COLLAGEN	Collagens	46	37	3.4e-43
KIR	Killer cell immunoglobulin-like receptors	7	14	1.1e-35
CYP	Cytochrome P450s	62	37	1.1e-33
IFF2	Intermediate filaments type II, keratins (basic)	26	23	2.6e-33
ZSCAN	-	54	33	3.2e-32
PSM	Proteasome (prosome, macropain) subunits	45	25	4.1e-25
PAR1	Pseudoautosomal regions / PAR1	18	15	1.7e-23
SERPIN	Serine (or cysteine) peptidase inhibitors	36	21	2.1e-23
GIMAP	GTPases, IMAP	7	10	5.3e-23
UGT	UDP glucuronosyltransferases	20	15	4.8e-22
ISET	Immunoglobulin superfamily / I-set domain containing	160	59	6.4e-21
comIV	Mitochondrial respiratory chain complex / Complex IV	16	13	8.5e-21
NBPF	neuroblastoma breakpoint family	11	11	1.6e-20
MT	Metallothioneins	14	12	3.5e-20
APOLIPO	Apolipoproteins	21	14	1.9e-19
complement	Complement system	35	18	3.8e-19
OR4	GPCR / Class A : Olfactory receptors	39	19	6.2e-19
SCARNA	ncRNAs / Small nucleolar RNAs : Small cajal body-specific	22	14	7.5e-19
IL	Interleukins and interleukin receptors	85	32	8.4e-19
OR51	GPCR / Class A : Olfactory receptors	18	12	2.4e-17
OR5	GPCR / Class A : Olfactory receptors	36	17	3.5e-17
CCL	Chemokine ligands	26	14	9.2e-17
OR52	GPCR / Class A : Olfactory receptors	24	13	5.6e-16
ENDOLIG	Endogenous ligands	230	77	7.9e-16
S100	S100 calcium binding proteins	21	12	1.1e-15
PLEKH	Pleckstrin homology (PH) domain containing	204	66	3.4e-15
SNORD	ncRNAs / Small nucleolar RNAs : C/D box containing	15	10	4.1e-15
LILR	Leukocyte immunoglobulin-like receptors	12	8	1.1e-12
IFN	Interferons	24	11	1.6e-12
WASH	WAS protein homologs	4	5	1.7e-12
KIF	Kinesins	42	15	3.0e-12
comI	Mitochondrial respiratory chain complex / Complex I	38	14	3.9e-12
OR1	GPCR / Class A : Olfactory receptors	25	11	3.9e-12
BTN	Butyrophilins	14	8	1.5e-11
CLEC	C-type lectin domain containing	46	15	4.2e-11
APOBEC	Apolipoprotein B mRNA editing enzymes	11	7	4.3e-11
TAS2R	Taste receptors / Type 2	24	10	7.4e-11
MRPL	Mitochondrial ribosomal proteins / large subunits	49	15	2.5e-10
SPDY	Speedy homologs	9	6	4.7e-10
TASNR	GPCR / Unclassified : Taste receptors	27	10	8.0e-10
WFDC	WAP four-disulfide core domain containing	18	8	1.1e-09
GABRA	Ligand-gated ion channels / GABA(A) receptors	19	8	2.6e-09
LCN	Lipocalins	20	8	6.1e-09
KLR	Killer cell lectin-like receptors	11	6	6.9e-09
GABR	GABA receptors	21	8	1.4e-08
PHF	Zinc fingers, PHD-type	76	19	1.6e-08
SLRR	Proteoglycans / Extracellular Matrix : Small leucine-rich repeats	12	6	2.1e-08
SRSF	Serine/arginine-rich splicing factors	12	6	2.1e-08
ADH	Alcohol dehydrogenases	8	5	2.7e-08
POTE	POTE ankyrin domain containing	8	5	2.7e-08
OR8	GPCR / Class A : Olfactory receptors	17	7	2.9e-08
OR10	GPCR / Class A : Olfactory receptors	33	10	4.3e-08
NKL	Homeoboxes / ANTP class : NKL subclass	50	13	8.3e-08
BPIF	BPI fold containing	14	6	1.5e-07
EFHAND	EF-hand domain containing	223	57	1.7e-07
TUB	Tubulins	25	8	2.3e-07
SH2D	SH2 domain containing	101	23	2.5e-07
ACOT	Acyl CoA thioesterases	10	5	3.3e-07
MYHII	Myosins / Myosin superfamily : Class II	15	6	3.7e-07
OR56	GPCR / Class A : Olfactory receptors	6	4	4.2e-07
ITG	Integrins	26	8	4.3e-07
MCDH	Cadherins / Major cadherins	32	9	5.4e-07
BTBD	BTB/POZ domain containing	134	30	7.9e-07
SIGLEC	Sialic acid binding Ig-like lectins	16	6	8.2e-07
TMPRSS	Serine peptidases / Transmembrane	17	6	1.8e-06
GGT	Gamma-glutamyltransferases	8	4	6.3e-06
bHLH	Basic helix-loop-helix proteins	106	22	7.2e-06
CACN	Calcium channel subunits	26	7	1.2e-05
SDRC1	Short chain dehydrogenase/reductase superfamily / Classical SDR fold cluster 1	20	6	1.3e-05
FN3	Fibronectin type III domain containing	153	32	2.4e-05

SIRP	Signal-regulatory proteins	5	3	4.2e-05
TTC	Tetratricopeptide (TTC) repeat domain containing	107	21	4.4e-05
IFT	Intraflagellar transport homologs	23	6	7.0e-05
SIX	Homeoboxes / SINE class	6	3	1.6e-04
ZMYM	Zinc fingers, MYM type	6	3	1.6e-04
DEFB	Defensins, beta	18	5	1.6e-04
FIBC	Fibrinogen C domain containing	25	6	1.9e-04
ADAMTS	ADAM metallopeptidases with thrombospondin type 1 motif	19	5	2.8e-04
OR6	GPCR / Class A : Olfactory receptors	27	6	4.7e-04
AQP	Ion channels / Aquaporins	14	4	7.8e-04
LGALS	Lectins, galactoside-binding	14	4	7.8e-04

Table S7 GPEA analysis of the RNAseq UC GRN for gene family gene sets. Shown is the protein family name abbreviation (tag), gene family name (name), the number of genes in the corresponding GRN subnetwork (genes), the number of interactions in the corresponding GRN subnetwork (edges) and the corresponding Bonferroni adjusted p-value (bonf).

GPEA analysis of the Bead UC GRN for gene families

tag	name	genes	edges	bonf
HIST	Histones / Replication-dependent	61	154	5.7e-252
ZKRAB	-	306	289	5.3e-155
CD	CD molecules	365	319	1.1e-140
SNORD	ncRNAs / Small nucleolar RNAs : C/D box containing	36	73	1.3e-129
ZNF	Zinc fingers, C2H2-type	641	515	4.0e-114
RPL	L ribosomal proteins	55	49	2.2e-59
HLA	Histocompatibility complex	25	33	2.9e-57
C1SET	Immunoglobulin superfamily / C1-set domain containing	38	34	1.5e-46
HOXL	Homeoboxes / ANTP class : HOXL subclass	50	39	1.8e-46
UGT	UDP glucuronosyltransferases	20	17	2.3e-27
KLK	Kallikreins	16	15	2.8e-26
MT	Metallothioneins	12	12	7.1e-23
IGD	Immunoglobulin superfamily / Immunoglobulin-like domain containing	221	77	5.1e-22
COLLAGEN	Collagens	43	21	1.1e-21
PCDHC	Cadherins / Protocadherins : Clustered	56	24	6.6e-21
KRT	-	51	20	2.8e-17
RPS	S ribosomal proteins	32	14	3.1e-15
LNCRNA	Long non-coding RNAs	468	168	2.0e-14
VSET	Immunoglobulin superfamily / V-set domain containing	150	42	5.2e-14
ENDOLIG	Endogenous ligands	221	62	3.3e-13
PSM	Proteasome (prosome, macropain) subunits	43	15	5.0e-13
KRTAP	Keratin associated proteins	75	22	5.3e-13
RBM	RNA binding motif (RRM) containing	186	50	1.6e-12
comI	Mitochondrial respiratory chain complex / Complex I	38	12	2.6e-10
CCL	Chemokine ligands	21	8	3.4e-09
SGST	Glutathione S-transferases / Soluble	17	7	8.6e-09
IFF2	Intermediate filaments type II, keratins (basic)	24	8	3.0e-08
comIV	Mitochondrial respiratory chain complex / Complex IV	15	6	1.3e-07
S100	S100 calcium binding proteins	21	7	1.8e-07
SERPIN	Serine (or cysteine) peptidase inhibitors	35	8	1.2e-05
MRPL	Mitochondrial ribosomal proteins / large subunits	49	10	1.6e-05
OR4	GPCR / Class A : Olfactory receptors	43	9	2.1e-05
SPDY	Speedy homologs	5	3	2.5e-05
PLEKH	Pleckstrin homology (PH) domain containing	195	38	5.5e-05
GIMAP	GTPases, IMAP	6	3	9.3e-05
complement	Complement system	33	7	1.0e-04
SLRR	Proteoglycans / Extracellular Matrix : Small leucine-rich repeats	12	4	1.1e-04
IFF1	Intermediate filaments type I, keratins (acidic)	26	6	1.1e-04
CYP	Cytochrome P450s	56	10	1.9e-04
ZSCAN	-	52	9	5.1e-04

Table S8 GPEA analysis of the Bead UC GRN for gene family gene sets. Shown is the protein family name abbreviation (tag), gene family name (name), the number of genes in the corresponding GRN subnetwork (genes), the number of interactions in the corresponding GRN subnetwork (edges) and the corresponding Bonferroni adjusted p-value (bonf).

GPEA analysis of the Oligo UC GRN for gene families

tag	name	genes	edges	bonf	
CD	CD molecules		351	319	8.9e-106
RPL	L ribosomal proteins		44	72	3.4e-102
ZKRAB	-		143	108	5.6e-64
ZNF	Zinc fingers, C2H2-type		362	242	2.3e-53
HIST	Histones / Replication-dependent		26	32	1.3e-48
RPS	S ribosomal proteins		29	28	6.1e-38
HLA	Histocompatibility complex		19	22	9.1e-36
C1SET	Immunoglobulin superfamily / C1-set domain containing		37	26	1.1e-28
RBM	RNA binding motif (RRM) containing		151	72	2.3e-28
PSM	Proteasome (prosome, macropain) subunits		42	26	7.7e-26
KLK	Kallikreins		14	14	1.7e-23
KRT	-		35	20	1.5e-20
MT	Metallothioneins		10	10	3.5e-18
CYP	Cytochrome P450s		48	22	1.2e-17
PCDHC	Cadherins / Protocadherins : Clustered		22	14	1.3e-17
IGD	Immunoglobulin superfamily / Immunoglobulin-like domain containing		177	67	2.6e-17
HOXL	Homeoboxes / ANTP class : HOXL subclass		43	20	5.2e-17
COLLAGEN	Collagens		33	17	5.5e-17
comI	Mitochondrial respiratory chain complex / Complex I		31	15	7.3e-15
IGL	Immunoglobulins / IGL locus		4	5	4.2e-12
ENDOLIG	Endogenous ligands		192	62	2.8e-11
VSET	Immunoglobulin superfamily / V-set domain containing		110	33	9.0e-11
comIV	Mitochondrial respiratory chain complex / Complex IV		12	7	6.3e-10
IFN	Interferons		20	9	6.3e-10
PAR1	Pseudoautosomal regions / PAR1		14	7	6.4e-09
UGT	UDP glucuronosyltransferases		7	5	1.4e-08
IFF2	Intermediate filaments type II, keratins (basic)		15	7	1.8e-08
S100	S100 calcium binding proteins		17	7	1.1e-07
complement	Complement system		30	9	9.6e-07
FATP	Mitochondrial respiratory chain complex / Complex V		16	6	2.5e-06
LNCRNA	Long non-coding RNAs		107	25	5.4e-06
SGST	Glutathione S-transferases / Soluble		14	5	3.0e-05
F1ATP	ATPases / F-type		15	5	6.2e-05
PRSS	Serine peptidases / Serine peptidases		22	6	1.2e-04
SERPIN	Serine (or cysteine) peptidase inhibitors		30	7	3.1e-04
CHAP	Heat Shock Proteins / Chaperonins		12	4	4.3e-04
IL	Interleukins and interleukin receptors		71	14	5.1e-04
bZIP	basic leucine zipper proteins		38	8	6.8e-04
KLR	Killer cell lectin-like receptors		7	3	7.5e-04

Table S9 GPEA analysis of the Oligo UC GRN for gene family gene sets. Shown is the protein family name abbreviation (tag), gene family name (name), the number of genes in the corresponding GRN subnetwork (genes), the number of interactions in the corresponding GRN subnetwork (edges) and the corresponding Bonferroni adjusted p-value (bonf).

GRN comparison to Protein-Protein Interaction databases

	F-score	TP	p-value	precision	recall	GRN	REF	genes
cspdb (pathways)								
RNAseq	0.006994	1,975	0	0.01839	0.004318	107,400	457,400	15,270
Bead	0.005064	1,267	0	0.01486	0.003052	85,290	415,100	14,430
Oligo	0.007169	1,411	0	0.021	0.004322	67,180	326,500	11,130
signaling (pathways)								
RNAseq	0.01468	560	0	0.02758	0.01	20,310	55,980	6,118
Bead	0.009161	309	6.17e-143	0.0202	0.005924	1,530	52,160	5,874
Oligo	0.01143	368	2.87e-167	0.02228	0.007682	16,510	47,910	5,300
graphite (pathways)								
RNAseq	0.01831	876	0	0.04561	0.01145	19,210	76,490	6,111
Bead	0.01191	512	3.04e-281	0.03386	0.007226	15,120	70,860	5,919
Oligo	0.01538	564	4.49e-305	0.03825	0.009623	14,740	58,610	5,003
hprd (ppi)								
RNAseq	0.006366	263	5.493e-128	0.005732	0.007157	45,880	36,750	9,581
Bead	0.004072	144	1.063e-49	0.003931	0.004223	36,630	3,410	9,147
Oligo	0.005564	184	3.574e-70	0.005177	0.006013	35,540	3,060	7,956
intact (ppi)								
RNAseq	0.003903	167	7.619e-50	0.003591	0.004274	4,650	39,080	9,571
Bead	0.002743	103	1.767e-20	0.002649	0.002845	38,890	36,210	9,136
Oligo	0.004339	138	1.187e-38	0.004133	0.004567	33,390	30,220	7,621
mint (ppi)								
RNAseq	0.004488	81	5.817e-28	0.003968	0.005164	20,410	15,690	6,153
Bead	0.002382	38	5.376e-06	0.002166	0.002646	17,540	14,360	5,881
Oligo	0.004089	59	2.113e-16	0.00361	0.004715	16,340	12,510	5,203
biogrid (ppi)								
RNAseq	0.005955	694	4.35e-277	0.00689	0.005243	100,700	132,400	14,760
Bead	0.003816	388	3.542e-102	0.004831	0.003153	80,310	123,100	13,920
Oligo	0.006354	527	1.183e-176	0.008346	0.00513	63,140	102,700	10,780
stringDB (ppi)								
RNAseq	0.01609	18,290	0	0.1277	0.008585	143,200	2,130,000	17,790
Bead	0.01217	12,650	0	0.1151	0.006426	110,000	1,969,000	16,760
Oligo	0.01652	12,880	0	0.1659	0.008693	77,630	1,482,000	11,910

Table S10 GRN comparison to popular ppi and pathway databases. Shown are the estimated F-scores, precision, recall and the number of overlapping edges (TP) for the subnetworks of shared genes between the GRN and the reference networks. The total number of interactions of shared genes between two networks is shown in column GRN for the gene regulatory network and REF for the corresponding protein network. In addition, we performed a hypergeometric test with the alternative hypothesis that the number of overlapping edges is larger than expected by random chance. Shown are Bonferroni adjusted p-values.

	median 1	median 2	p-value
GO			
**RNAseq-Bead**	0.044151	0.029482	0.000007
RNAseq-Oligo	0.044151	0.039358	0.169625
**Bead-Oligo**	0.029482	0.039358	0.002390
GE			
RNAseq-Bead	0.255194	0.185957	0.000675
RNAseq-Oligo	0.255194	0.256114	0.968368
Bead-Oligo	0.185957	0.256114	0.001697
GF			
RNAseq-Bead	0.150451	0.122684	0.382322
RNAseq-Oligo	0.150451	0.172519	0.592284
Bead-Oligo	0.122684	0.172519	0.255325

Table S11 Pairwise comparison of the F-score distributions between the RNAseq, Bead and Oligo GRN. Shown are the median F-scores and nominal p-values from a pairwise t-test.
